# Immune signatures underlying post-acute COVID-19 lung sequelae

**DOI:** 10.1126/sciimmunol.abk1741

**Published:** 2021-11-12

**Authors:** I. S. Cheon, C. Li, Y. M. Son, N. P. Goplen, Y. Wu, T. Cassmann, Z. Wang, X. Wei, J. Tang, Y. Li, H. Marlow, S. Hughes, L. Hammel, T. M. Cox, E. Goddery, K. Ayasoufi, D. Weiskopf, J. Boonyaratanakornkit, H. Dong, H. Li, R. Chakraborty, A. J. Johnson, E. Edell, J. J. Taylor, M. H. Kaplan, A. Sette, B. J. Bartholmai, R. Kern, R. Vassallo, J. Sun

**Affiliations:** 1 Division of Pulmonary and Critical Medicine, Department of Medicine, Mayo Clinic, Rochester, MN 55905, USA.; 2 Department of Immunology, Mayo Clinic, Rochester, MN 55905, USA.; 3 Division of Biomedical Statistics and Informatics, Mayo Clinic, Rochester, MN 55905, USA.; 4 Center for Infectious Disease and Vaccine Research, La Jolla Institute for Immunology (LJI), La Jolla, CA 92037, USA.; 5 Vaccine and Infectious Disease Division, Fred Hutchinson Cancer Research Center, Seattle, WA 98109, USA.; 6 Department of Molecular Pharmacology and Experimental Therapeutics, Mayo Clinic, Rochester, MN 55905, USA.; 7 Department of Pediatrics and Adolescent Medicine, Mayo Clinic, Rochester, MN 55905, USA.; 8 Department of Microbiology and Immunology, Indiana University of School of Medicine, Indianapolis, IN 46202, USA.; 9 Department of Medicine, Division of Infectious Diseases and Global Public Health, University of California San Diego (UCSD), La Jolla, CA 92037, USA.; 10 Department of Radiology, Mayo Clinic, Rochester, MN 5590, USA.; 11 Department of Physiology and Biomedical Engineering, Mayo Clinic, Rochester, MN 55905, USA.; 12 Robert and Arlene Kogod Center on Aging, Mayo Clinic, Rochester, MN 55905, USA.; 13 Carter Immunology Center, University of Virginia, Charlottesville, VA 22908, USA.; 14 Division of Infectious Disease and International Health, Department of Medicine, University of Virginia, Charlottesville, VA 22908, USA.

## Abstract

Severe coronavirus disease 2019 (COVID-19) pneumonia survivors often exhibit long-term pulmonary sequelae, but the underlying mechanisms or associated local and systemic immune correlates are not known. Here, we have performed high-dimensional characterization of the pathophysiological and immune traits of aged COVID-19 convalescents, and correlated the local and systemic immune profiles with pulmonary function and lung imaging. We found that chronic lung impairment was accompanied by persistent respiratory immune alterations. We showed that functional severe acute respiratory syndrome coronavirus 2 (SARS-CoV-2)–specific memory T and B cells were enriched at the site of infection compared with those of blood. Detailed evaluation of the lung immune compartment revealed that dysregulated respiratory CD8^+^ T cell responses were associated with the impaired lung function after acute COVID-19. Single-cell transcriptomic analysis identified the potential pathogenic subsets of respiratory CD8^+^ T cells contributing to persistent tissue conditions after COVID-19. Our results have revealed pathophysiological and immune traits that may support the development of lung sequelae after SARS-CoV-2 pneumonia in older individuals, with implications for the treatment of chronic COVID-19 symptoms.

## Teaser

Respiratory tissue resident–like CD8^+^ T cells are associated with chronic lung sequelae post-acute COVID-19.

## INTRODUCTION

The main determinant of clinical outcomes in individuals with severe acute respiratory syndrome coronavirus 2 (SARS-CoV-2) infection is age, with the majority of morbidity and mortality occurring in the elderly (*1*, *2*). Beyond the acute morbidity induced by SARS-CoV-2 infection, data have shown that patients with severe acute coronavirus disease 2019 (COVID-19) illness are at highest risk of developing chronic pulmonary sequelae and persistent chest imaging abnormalities (*3*–*6*). However, the mechanisms underlying the development of chronic lung sequelae after acute SARS-CoV-2 infection remain elusive. As the number of patients with symptoms persisting after resolution of acute illness rises (so-called chronic COVID-19), there is an urgent need to identify the cellular, molecular, and pathophysiological characteristics of chronic lung sequelae in COVID-19 convalescents. Elucidation of specific mechanisms by which chronic lung injury develops is essential for the development of future preventive and/or therapeutic interventions for chronic COVID-19 symptoms.

T cells and B cells can form long-lived immunological memory after the clearance of primary viral infection to protect the host from reinfection of the same or related viruses (*7*). Memory lymphocytes are generally divided into circulating memory cells that patrol the body and tissue-resident memory cells that reside in the peripheral non-lymphoid tissue (*7*, *8*). Tissue-resident T (T_RM_) and B (B_RM_) cells that reside within the respiratory tract provide immediate and superior immunity against viral reinfections (*9*), but dysregulated lung-resident T cell responses have also been linked with chronic lung inflammation, pathology, and fibrosis after respiratory viral infection, particularly in older hosts (*10*, *11*). Evidence has suggested that antigen-specific B cell and T cell memory is formed and can persist in the circulation for more than 6 months after SARS-CoV-2 infection (*12*, *13*). However, tissue-specific memory responses in the respiratory tract have not been previously characterized after SARS-CoV-2 infection. The current report describes detailed characterization of circulating and respiratory immune profiles and quantitative lung functional and pathological parameters in a cohort of aged COVID-19 convalescents. Our results suggest that dysregulated pulmonary immune responses, particularly exuberant responses of certain respiratory CD8^+^ T cell subsets, may contribute to the development of chronic lung sequelae after resolution of acute COVID-19 infection in aged individuals.

## RESULTS

### Pathophysiological characterization of lung sequelae after COVID-19

**Fig. 1.**
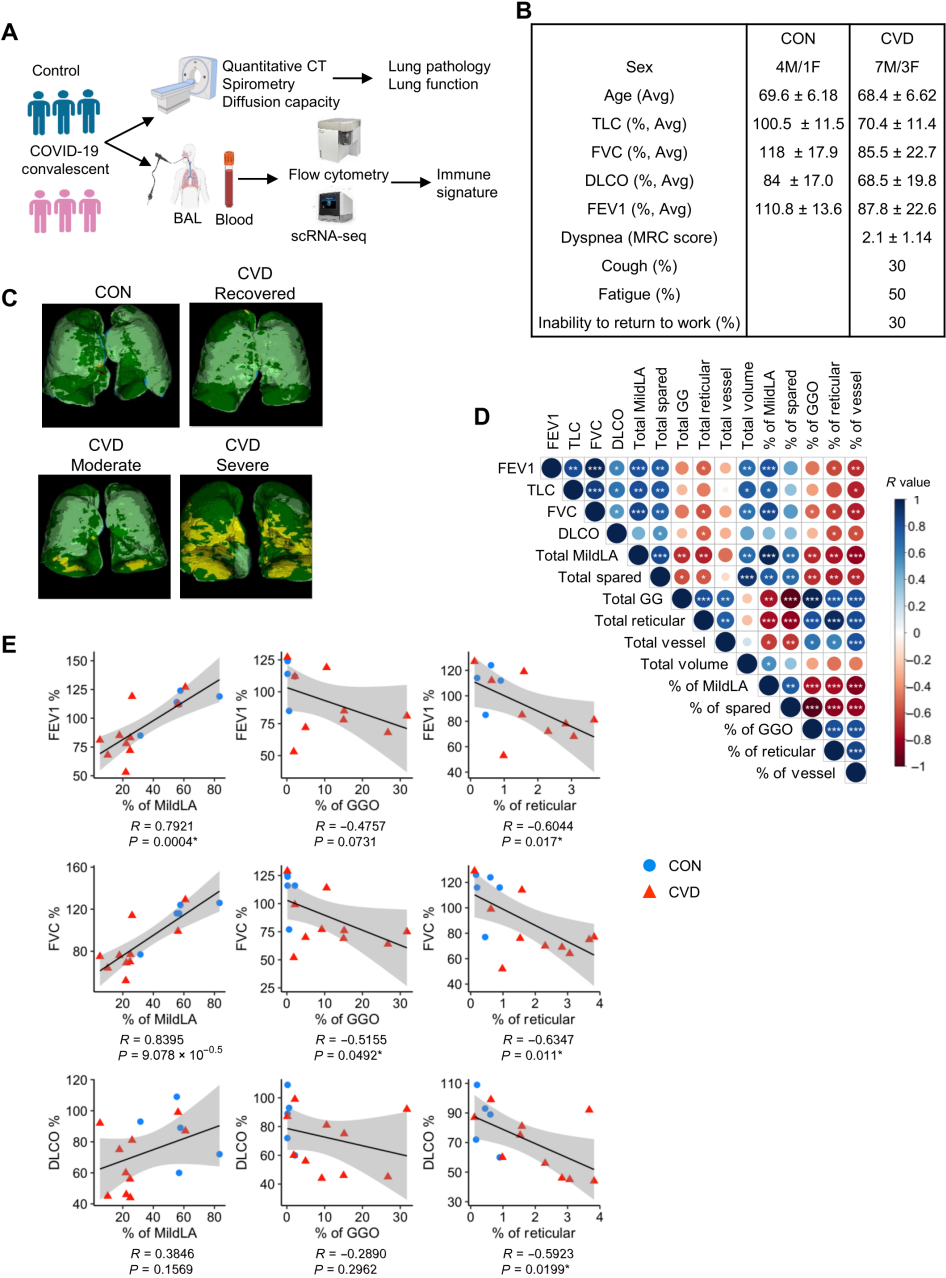
Functional and pathological characterization of lung sequelae in aged COVID-19 convalescents. (**A**) Schematics of experimental procedure. (**B**) Summary of control (CON) and COVID-19 convalescent age, sex, lung function, and condition data. (**C**) Lung regions of pathology revealed by quantitative CT analysis by CALIPER (green/dark green, “spared/relatively spared”; yellow, “GGO”; orange, “reticular/consolidation”). (**D**) Correlation of lung functional parameters with lung pathological features. Significant correlations were indicated by white asterisks. (**E**) Correlation of FEV1, FVC, and DLCO with % of mild lung area (MildLA), % of GGO region, and % of reticular regions in the lung. Significant correlations were indicated by asterisks. *P < 0.05.

Aged hosts develop chronic lung inflammatory and fibrotic sequelae after viral pneumonia in an animal model (*11*). We hypothesized that aged individuals who survived moderate to severe acute COVID-19 infection may experience persistent lung inflammation and fibrosis and impaired lung function. To this end, we recruited a cohort of aged healthy control and COVID-19 convalescents (all >60 years of age) discharged from hospital and evaluated 60 to 90 days after onset of SARS-CoV-2 infection (Fig. 1, A and B, and fig. S1A). Most of these individuals were unvaccinated (fig. S1A and data file S1). There were two exceptions: one in control (CON) who completed two doses of mRNA vaccination 4 weeks before the bronchoscopy procedure, and one in the COVID-19 convalescent group (CVD) who completed two doses of mRNA vaccination 1 week before the bronchoscopy procedure. The COVID-19 convalescent who had completed vaccination remained SARS-CoV-2 polymerase chain reaction positive (PCR^+^) but showed no signs of active infection at the time of enrollment in the study. However, this was not confirmed by SARS-CoV-2 N1 gene reverse transcription PCR (RT-PCR) in the laboratory because we found that none of the COVID-19 convalescents harbored SARS-CoV-2 N1 gene in the bronchoalveolar lavage fluid (BAL) (fig. S1B). There were no preexisting lung conditions before SARS-CoV-2 infection in these individuals, and so all abnormalities detected on imaging or lung function testing were likely attributable to SARS-CoV-2 infection. All COVID-19 convalescents had persistent pulmonary and extrapulmonary symptoms at the time of study enrollment (Fig. 1, A and B, and data file S1), suggesting that they were experiencing post-acute sequelae of COVID-19 (PASC). We obtained BAL and blood from these individuals and performed high-dimensional spectral flow cytometry and single-cell RNA sequencing (scRNA-seq) on BAL and blood immune cells (Fig. 1A). We also conducted pulmonary function tests (spirometry and lung diffusion capacity) and quantitative computerized tomography (CT) using the automated computer-aided lung informatics for pathology evaluation and rating (CALIPER) technique (Fig. 1C and fig. S1, C to E) (*14*).

Lungs of aged COVID-19 convalescents showed heterogeneous imaging features suggestive of ongoing inflammation and fibrosis. Whereas a couple of individuals had almost full lung recovery from the infection (CVD Recovered), most patients (>70%) exhibited moderate (CVD Moderate) to severe (CVD Severe) CT abnormalities (yellow and orange regions; Fig. 1C and fig. S1, D and E) and impaired pulmonary function more than 2 months after hospital discharge (Fig. 1B, fig. S1C, and data file S1). Lung function parameters were abnormal in several of the COVID-19 cohort, with physiologic evidence of a pulmonary parenchymal restrictive abnormality [reduced TLC (total lung capacity) and FVC (forced vital capacity)] evident in several individuals. Pulmonary function parameters including FEV1 (forced expiratory volume in 1 s), FVC, and DLCO (diffusion capacity for carbon monoxide) were inversely correlated with the extent of lung ground glass opacification (GGO; yellow regions), reticular densities and consolidation (orange regions), and/or vessel-related structures in the CT image (Fig. 1, D and E) and positively correlated with residual normal or relatively spared lung regions (total volume or percentage of light and dark green regions). These data suggest that the extent of tissue pathology after acute COVID-19 determines the pulmonary gas exchange function of aged COVID-19 convalescents. Most of the aged COVID-19 convalescents showed decreased normal lung regions, and increased tissue exhibiting GGO-, reticular-, and vascular-related structures, accompanied by decreased FEV1, FVC, DLCO, and other lung functional parameters (Fig. 1E; fig. S1, C to E; and data files S1 and S2). Together, our detailed characterization of lung pathophysiological parameters demonstrates that acute SARS-CoV-2 infection resulted in long-term lung imaging and functional sequelae in a cohort of aged individuals.

### Respiratory and systemic immune traits of aged COVID-19 convalescents

**Fig. 2.**
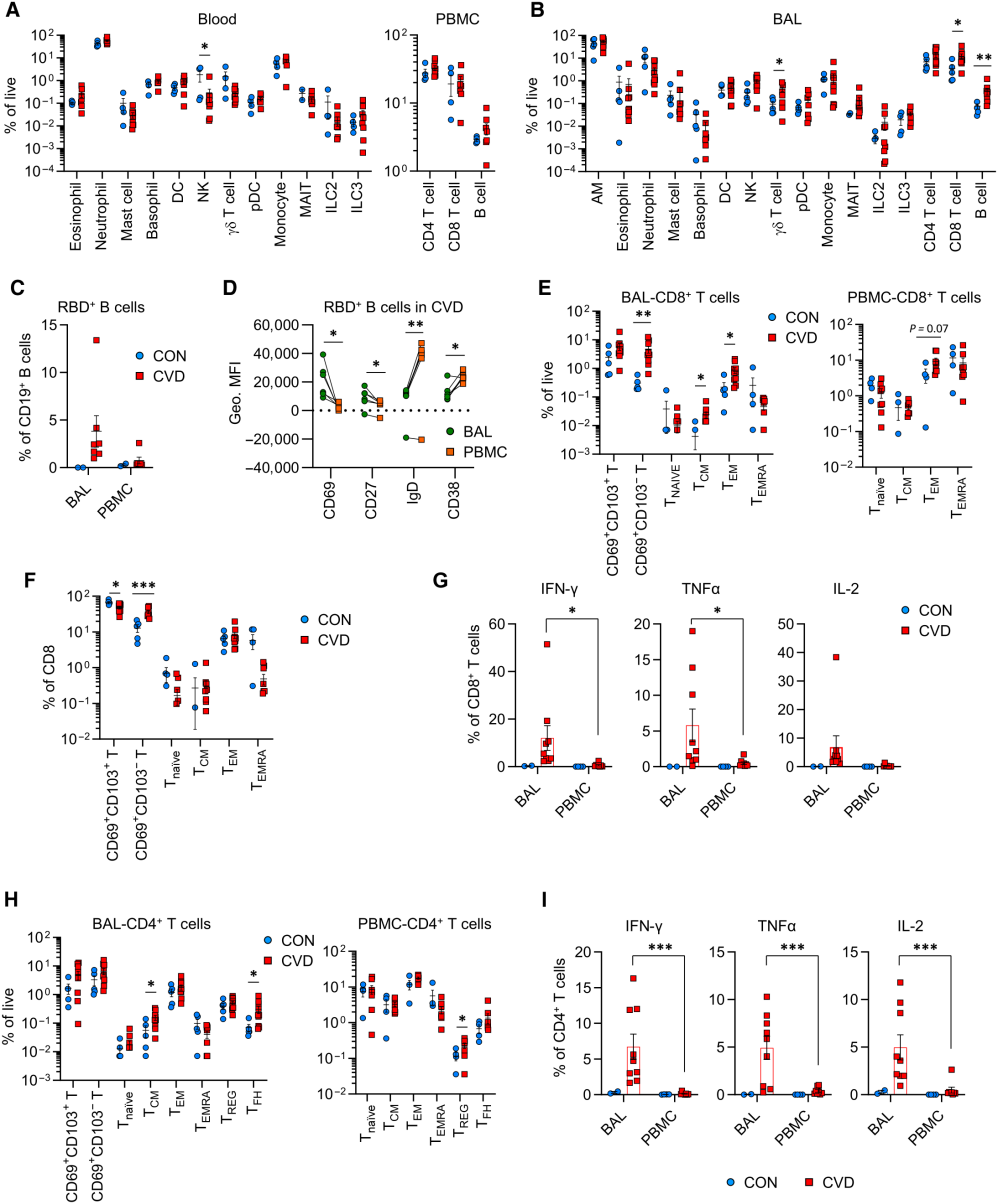
Characterization of respiratory and circulating immune memory in COVID-19 convalescents. (**A**) Percentage of indicated innate immune cell types in the whole blood (left) or adaptive immune cells in PBMCs (right). (**B**) Percentage of indicated immune cell types in the BAL. (**C**) Percentage of RBD-specific B cells within B cell compartment of BAL or PBMC. (**D**) CD69, CD27, IgD, and CD38 expression levels of RBD-specific B cells in BAL or PBMC. (**E**) Percentage of indicated CD8^+^ T cell subsets based on live cells in the BAL or PBMC. (**F**) Percentage of indicated CD8^+^ T cell subsets in total CD8^+^ T cells. (**G**) Percentage of IFN-γ, TNF, or interleukin (IL)–2^+^ CD8^+^ T cells in the BAL or PBMC after stimulation with SARS-CoV-2 peptide pools. (**H**) Percentage of indicated CD4^+^ T cell subsets based on live cells in the BAL or PBMC. (**I**) Percentage of IFN-γ, TNF, or IL-2^+^ CD4^+^ T cells in the BAL or PBMC after stimulation with SARS-CoV-2 peptide pools. (A, B, E, F, and H) Statistical significance was calculated using Mann-Whitney test. *P < 0.05. (C) Statistical difference was performed using two-way ANOVA. (D) Statistical significance was calculated using paired *t* test. (G and I) Statistical significance was calculated using two-way ANOVA following Fisher’s least significant difference (LSD) test. *P < 0.05.

The levels of most proinflammatory cytokines were comparable in the blood or respiratory tract of the control and COVID-19 convalescents (fig. S2), which is in contrast to what was reported during the acute stage of COVID-19 (*15*, *16*) and indicates that the acute inflammatory responses had largely resolved in the convalescent stage. To determine the systemic and pulmonary immune cell profile, high-dimensional flow cytometric analysis was performed on circulating and respiratory immune cells in control and aged COVID-19 convalescents (figs. S3 and S4). Although most of immune cell types (except natural killer cells) were comparable in the circulation between control and COVID-19 convalescents (Fig. 2A), there was an increase in the frequency of γ/δ T cells, B cells, and CD8^+^ T cells within the respiratory compartment of the COVID-19 convalescent cohort compared with those of control (Fig. 2B). Among the subtypes of increased BAL immune cells, CD8^+^ T cells appeared to be the most abundant population (3.54 to 12.29%) (Fig. 2B). Thus, SARS-CoV-2 infection leads to persistent alterations of respiratory immune profiles at the convalescent stage.

### Characterization of respiratory immune memory after SARS-CoV-2 infection

Sustained circulating antibody and memory lymphocyte responses have been detected months after acute SARS-CoV-2 infection (*17*). However, the characteristics of antibody and memory lymphocyte responses in the lower respiratory tract have not been reported. As expected, elevated immunoglobulin G (IgG) and IgA antibody responses against SARS-CoV-2 protein N, S1, and RBD (S receptor binding domain), were detected in both plasma and BAL of COVID-19 convalescents compared with those of control; evidence of humoral S1 and RBD IgG responses were detected in one control individual that had received COVID-19 vaccination (fig. S5A). SARS-CoV-2–specific IgM responses were less distinct between healthy controls and the COVID-19 convalescent group (fig. S5B). RBD-specific B cells were detected in both peripheral blood mononuclear cell (PBMC) and BAL, and there was a trend of increase in the percentages of RBD-specific B cells within the total BAL B cell population, suggesting that mucosal B cell pool is likely concentrated with antigen-specific memory B cells at the site of infection (Fig. 2C). Compared with circulating RBD-specific B cells, BAL RBD-specific B cells had increased CD69 and CD27 (Fig. 2D and fig. S4) but diminished IgD and CD38, indicating that these SARS-CoV-2–specific B cells have a resident memory phenotype. The levels of BAL SARS-CoV-2–specific IgG responses were correlated with local CD4^+^ T cell levels, particularly the abundance of tissue resident–like CD69^+^ CD4^+^ T cells (fig. S5, C and D), indicating that CD4^+^ T cells may be required for sustaining respiratory antibody responses at the memory stage after the recovery of primary respiratory viral infection, as was observed in animal models (*18*, *19*).

We next analyzed the presence of different CD8^+^ T cell population, including naïve, effector memory (T_EM_), central memory (T_CM_), effector memory T cells reexpressing CD45RA (T_EMRA_), and T_RM_ cells in the BAL and PBMC of control and COVID-19 convalescents (fig. S6, A to D). CD69^+^ T_RM_-like cell (including CD69^+^CD103^+^ conventional T_RM_ and CD69^+^CD103^−^ T cells) presence was increased in the respiratory tract of COVID-19 convalescents compared with those of aged controls (Fig. 2E and fig. S6E). Within the BAL CD8^+^ T cell population, there appeared to be an enrichment of CD69^+^CD103^−^ T cells in COVID-19 convalescents (Fig. 2F). Compared with CD103^+^ T_RM_ cells, CD69^+^CD103^−^ T cells expressed higher levels of EOMES, granzyme B, granzyme K, CXCR4, and KLRG1 (fig. S6F). After stimulation with a pool of predicted overlapping SARS-CoV-2 peptides (*20*), there appeared to be higher percentages of cytokine [interferon-γ (IFN-γ) or tumor necrosis factor (TNF)]–producing cells in the BAL CD8^+^ T cell population compared with those of peripheral blood CD8^+^ T cells (Fig. 2G and fig. S6G), and the cytokine production between CD69^+^CD103^+^ T_RM_ and CD69^+^CD103^−^ T cells was comparable (fig. S6H). There were higher percentages of CD8^+^ T cells capable of producing multiple cytokines in the respiratory tract than those of cells in the circulation (fig. S6I), suggesting that SARS-CoV-2 infection results in polyfunctional memory CD8^+^ T cell generation in the respiratory mucosa.

Examination of CD4^+^ T cells demonstrated that percentages of CD4^+^ T_CM_ and follicular helper–like cells were elevated in the respiratory tract of COVID-19 convalescents compared with those of healthy control (Fig. 2H and fig. S7, A to D). In COVID-19 convalescents, there was an enrichment of SARS-CoV-2–specific CD4^+^ T cell presence in the respiratory tract, and BAL memory CD4^+^ T cells were more polyfunctional than those of blood memory CD4^+^ T cells (Fig. 2I and fig. S7, E to G). Together, these data suggest that functional B, CD4^+^, and CD8^+^ memory cells are present in the respiratory tract after recovery from acute COVID-19 pneumonia, and these memory T and B lymphocytes showed enhanced tissue residency characteristics compared with those of circulating memory counterparts.

### Dysregulated CD8^+^ T cell responses are associated with impaired lung function after viral pneumonia

**Fig. 3.**
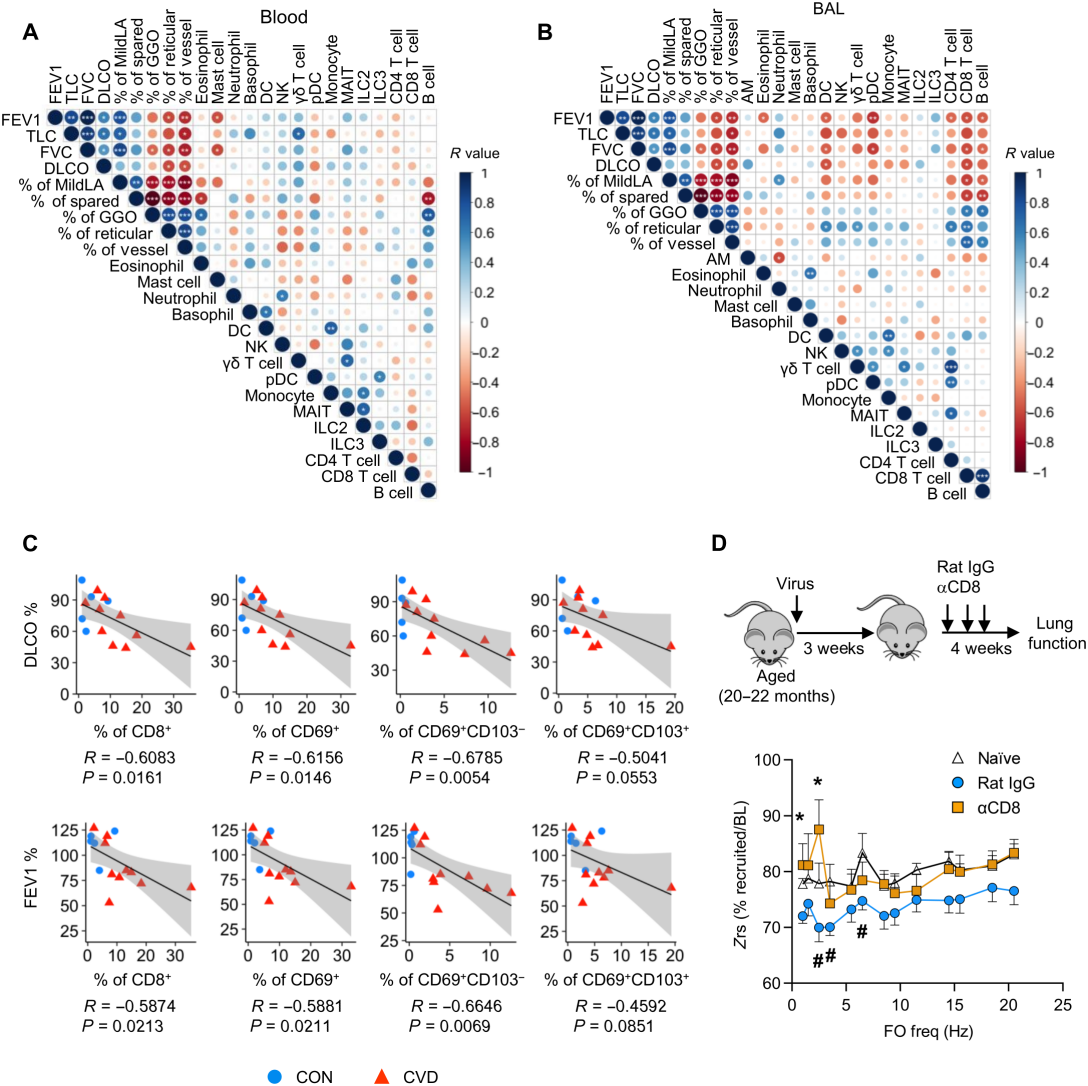
Dysregulated CD8^+^ T cell responses as a potential driver of chronic lung sequelae. (**A**) Correlation of blood immune cell types with lung functional parameters and quantitative lung pathology parameters. (**B**) Correlation of BAL immune cell types with lung functional parameters and quantitative lung pathology parameters. (**C**) Correlation of DLCO and FEV1 with % of total CD8^+^, CD69^+^ CD8^+^, CD69^+^CD103^−^ CD8^+^, or CD69^+^CD103^+^ CD8^+^ T cells in the total BAL cells of healthy control and COVID-19 convalescents. (**D**) Aged mice were infected or not (naïve) with influenza virus. Mice receiving CD8^+^ T cell–depleting antibody (αCD8) or control antibody (rat IgG) starting at 21 days after infection. Lung function was measured at 50 days after infection. Input impedance (*Z*_rs_) was measured during tidal breathing and after recruitment of closed airways and displayed as the percent utilization at baseline for each frequency in the repeated forced oscillation (FO) waveform. (A and B) *R* values are indicated by color and circle size. Significant correlations were indicated by white asterisks. (D) Lung function data were cumulative from three independent experiments, and raw data can be found in fig. S9. *P < 0.05 for rat IgG (*n* = 16) versus CD8-depleted group (*n* = 8) or naïve (*n* = 7) versus rat IgG group (#) for two-way ANOVA following Fisher’s LSD test.

We examined the association of lung function and imaging abnormalities with respiratory and circulating immune profiles. The levels of circulating mast cells were negatively, whereas γ/δ T cells were positively, correlated with better lung function in COVID-19 convalescents (Fig. 3A). Circulating B cell levels appeared to be positively correlated with lung pathological parameters (Fig. 3A). In the respiratory tract, the levels of eosinophils, dendritic cells (DCs), plasmacytoid DCs, CD4^+^ T cells, and B cells were negatively correlated with lung function (FEV1 and FVC) or percentages of normal or relatively preserved parenchymal imaging lung characteristics (percentage of mild low attenuation and spared lung on imaging; Fig. 3B). Respiratory CD8^+^ T cells were negatively correlated with lung function parameters and positively correlated with lung pathological parameters, suggesting a potential role of respiratory CD8^+^ T cells in contributing to the development of lung sequelae after acute COVID-19 (Fig. 3B).

CD8^+^ T_RM_-like cells, particularly CD69^+^CD103^−^ T cells, were negatively correlated with lung function (DLCO and FEV1) in combined healthy control and COVID-19 convalescent groups (Fig. 3C) or in COVID-19 convalescent group alone (fig. S8). Our cohort contained relatively fewer female individuals because most severe COVID-19 cases occur in male sex. Because sex differences and/or vaccine administration may alter the dynamics of T cell responses and tissue recovery, we have also performed correlation analysis of respiratory CD8^+^ T cell levels with lung function after the exclusion of vaccinated and/or female individuals (fig. S9). BAL CD69^+^CD103^−^ T cell levels remained negatively correlated with lung functional parameters in this setting (fig. S9). Together, these data indicate that dysregulated CD8^+^ T_RM_ cell composition or responses in aged COVID-19 convalescents may contribute to the impaired lung function and/or development of chronic lung sequelae.

Our data are in line with the findings that the depletion of CD8^+^ T cells led to decreased chronic inflammation and lung pathology after influenza viral pneumonia during aging (*11*), although the roles of CD8^+^ T cells in lung function after acute pneumonia were not studied. To this end, we used the reported murine model of chronic lung sequelae after influenza pneumonia during aging (*11*). We infected aged mice (20 to 22 months old) with influenza A virus (PR8 strain) and then depleted CD8^+^ T cells at 3 weeks after infection, which mice have recovered from the primary infection. Four to 5 weeks later, we measured lung function using forced oscillation technique (FOT) by flexiVent. FOT allows for the distinction of mechanics arising from small (*G*_ti_) and large (*R*_n_) airways. With respect to maximum lung capacity, infection increased the resistance to airflow in the small, but not large, airways at baseline. This dysfunction in the small airways was rescued by the depletion of CD8^+^ T cells in aged mice after the recovery of primary viral pneumonia (fig. S10). To ascertain whether terminal airways were affected, the extent of dysfunction throughout the airway tree was analyzed from input impedances (*Z*_rs_) of the FOT data. Under baseline breathing conditions, aged naïve mice used 75 to 80% of their lung capacity in both small and large airways. Infection reduced this utilization by a further 5 to 10% primarily in smaller airways. However, in CD8^+^ T cell–depleted animals, the smallest airways showed significant functional recovery after infection (Fig. 3D and fig. S10), indicating increased alveoli recruitment during tidal breathing. Together, this indicates that there is long-term infection-induced dysfunction in the alveoli driven by the presence of CD8^+^ T cells in aged hosts after viral pneumonia.

### Identification of potentially pathogenic respiratory CD8^+^ T cell subsets in aged COVID-19 convalescents

To gain further insights into the cellular and molecular characteristics of the respiratory and circulating memory T cell responses after SARS-CoV-2 infection, we performed scRNA-seq and profiled 27,167 BAL and blood T cells from six COVID-19 convalescents. Nine major T cell clusters across BAL and PBMC (fig. S11, A and B) and 12 subclusters of PBMC and BAL CD4^+^ T cells were identified (fig. S11, C and D). BAL CD4^+^ T cells showed distinct features of gene expression and tissue-resident gene programs with four major subclusters identified (fig. S11, E to J). Compared with age-matched or unmatched healthy controls, aged COVID-19 convalescents had increased presence of cluster 2 CD4^+^ T cells, which expressed genes associated with myeloid cell inflammation including Lyz, S100 A8, and S100 A9 (fig. S11, K to M) (*21*). T cell receptor (TCR) sequencing also revealed the presence of clonally expanded T cells, which potentially represented those SARS-CoV-2–specific T cells, within the circulation and BAL CD4^+^ T cell populations (fig. S12).

**Fig. 4.**
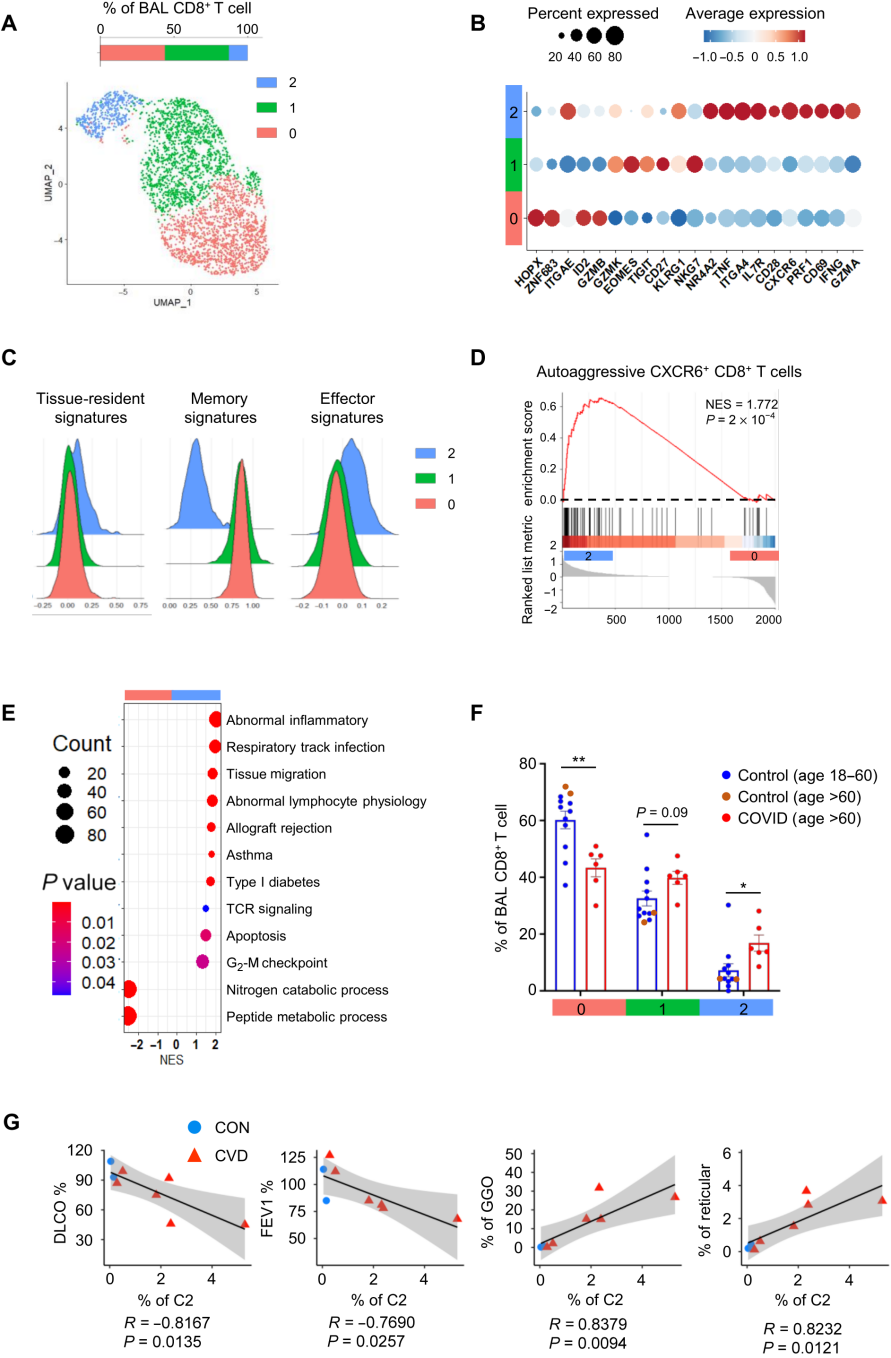
scRNA-seq analysis of respiratory CD8^+^ T cells from COVID-19 convalescents. (**A**) BAL CD8^+^ T cell subclusters and relative abundance revealed by scRNA-seq. (**B**) Signature gene expression by different BAL CD8^+^ T cell clusters. (**C**) Relative levels of tissue residency, memory, and effector gene signatures in BAL CD8^+^ T cell clusters. (**D**) GSEA of autoaggressive CXCR6^+^ CD8^+^ T cells between clusters 0 and 2. (**E**) Different pathways enriched in cluster 2 compared with cluster 0 of BAL CD8^+^ T cells. (**F**) Relative abundance of BAL CD8^+^ T cell subclusters from control [aged-matched control recruited in this study and age-unmatched control from GSE151928 (*42*)] or COVID-19 convalescent donors. (**G**) Correlation of DLCO, FEV1, GGO, and reticular region with % of cluster 2 CXCR6^hi^ CD8^+^ T cells in the BAL cells of age-matched control and COVID-19 convalescents.

Fourteen subclusters of blood and respiratory CD8^+^ T cell population were identified after scRNA-seq (fig. S13A). TCR sequencing identified the presence of clonally expanded T cells within the circulating and BAL CD8^+^ T cell populations (fig. S13, B to E). Furthermore, those clonally expanded T cells had higher levels of effector or cytotoxic molecules, supporting the view that those were likely antigen-experienced T cells (fig. S13, F and G). After excluding the cycling cell population, 3254 BAL CD8 T cells were further subclustered into three major clusters: cluster 0 [ITGAE (CD103)^hi^ ZNF683 (HOBIT)^hi^], composed of conventional CD103^+^ T_RM_ cells; cluster 1 (EOMES^hi^), composed of CD69^+^CD103^−/low^ T_RM_ cells; and cluster 2, CD8^+^ T cells expressing high levels CXCR6, PRF1, and TNF (Fig. 4, A and B, and fig. S14). Compared with circulating CD8^+^ T cells, respiratory CD8^+^ T cells had distinct features of gene expression and tissue-resident gene programs (fig. S14, A to C). Pseudotime analysis by Monocle 3 suggested that cluster 2 was less differentiated, whereas the conventional CD103^+^ cells were most differentiated clusters (fig. S14G). BAL CD103^low^ T_RM_ cells appeared to have the highest level of shared TCR sequences with PBMC CD8^+^ T cells (fig. S15). Further analysis identified that, although all three clusters exhibited a similar tissue residency gene program and down-regulation of genes associated with circulation (Fig. 4C, left, and fig. S14H), cluster 2 showed a lower T memory cell, but higher T effector cell, gene signature (Fig. 4C, middle and right). Clusters 1 and 0 shared comparable T memory cell signatures, but the TCR signaling gene set was enriched in cluster 1 (fig. S14I), indicating that CD103^+^ T_RM_ cells (cluster 0) had a more quiescent phenotype than CD69^+^CD103^−^ T cells (cluster 1). Cluster 1 CD103^−/low^ T_RM_ cells also expressed higher levels of NKG7, a cytotoxic molecule that can promote lethal inflammation after infection (*22*), and granzyme K, an inflammatory granzyme that promotes fibroblast activation (Fig. 4B and figs. S6F and S14F) (*23*) . Together, these data suggest that the cluster 1 CD103^−/low^ T_RM_ cells have higher pathogenic potential than those of cluster 0, the conventional T_RM_ cells.

A population of CXCR6^hi^ pathogenic tissue-resident CD8^+^ T cells was recently identified as a driver of tissue damage during nonalcoholic steatohepatitis (NASH) (*24*). Cluster 2 CXCR6^hi^ respiratory CD8^+^ T cells in COVID-19 convalescents were enriched with the liver pathogenic CXCR6^hi^ gene signature compared with those of cluster 0 (Fig. 4D and fig. S14J). Furthermore, compared with clusters 0 and 1, cluster 2 CXCR6^hi^ CD8^+^ T cells showed enriched gene programs associated with tissue-aggressive features, which include allograph rejection, asthma, and type I diabetes–related genes (Fig. 4E). Compared with both age-matched or unmatched healthy controls, aged COVID-19 convalescents had increased presence of cluster 2 CXCR6^hi^ CD8^+^ T cells within the BAL CD8^+^ T cell population (Fig. 4F and fig. S14K). In addition, the abundance of cluster 2 CXCR6^hi^ CD8^+^ T cells in BAL was negatively correlated with lung gas exchange function and positively associated with the extent of lung GGO and reticular consolidation (Fig. 4G). Together, our flow cytometric and scRNA-seq analysis have revealed marked heterogeneity in the respiratory CD8^+^ T cells after SARS-CoV-2 infection at the convalescent stage. Our data further support the view that conventional pulmonary CD103^+^ T_RM_ cells are less pathogenic in vivo, whereas the CD69^+^CD103^−^ T cells and/or CXCR6^hi^ effector-like tissue-resident cells exhibited more inflammatory and cytopathic features, potentially contributing to the persistent tissue pathology and impaired lung function observed in aged COVID-19 convalescents (fig. S16).

## DISCUSSION

A substantial proportion of patients with COVID-19 develop persistent symptoms after resolution of acute illness, which may be accompanied by abnormal chest imaging and impaired lung function testing (*25*). Using automated quantitative analysis, we found that areas of abnormal lung imaging directly correlated with abnormal lung function tests, suggesting that either incomplete tissue recovery even 60 days after the onset of COVID-19 pneumonia or persistent tissue remodeling with fibrosis results in the impaired respiratory function observed in some COVID-19 convalescents. We did not detect notable SARS-CoV-2 RNA in the BAL of COVID-19 convalescents in our cohorts, which argues against the hypothesis that persistent SARS-CoV-2 infection in the lung causes chronic pulmonary sequelae.

CD8^+^ T cells mainly provide protective function during acute respiratory viral infection including SARS-CoV-2 infection (*17*). However, because of their potent tissue injurious ability, prolonged and/or dysregulated CD8^+^ T cell activities can contribute substantially to lung injury and/or pathology development after respiratory viral infection (*26*). Consistent with our previous findings reported in an animal model that CD103^−^ T_RM_ cells have potent pathogenic potential (*10*), respiratory CD69^+^CD103^−^ T cells from aged COVID-19 convalescents were found to express higher levels of cytotoxic or inflammatory molecules and correlated with decreased lung function and worse lung pathology. Whether these human T cells are truly tissue resident or not in the respiratory tract needs further investigation. CD103^−^ T cells were enriched with genes associated with the TCR signaling pathway, indicating that they may be sustained by tonic or antigenic signaling within the injured lung. Persistent TCR signaling caused by residual antigen deposition in the lung has been shown to drive the maintenance of CD69^+^CD103^−^ T cells after murine influenza virus infection (*10*). It is thus possible that SARS-CoV-2 antigen may also be persistent for a period of time after infectious virus clearance, thereby sustaining and/or stimulating CD103^−^ T_RM_ cells to cause tissue injury. Alternatively, a portion of CD69^+^CD103^−^ T cells may be restricted and stimulated by self-antigen as those of autoantibodies reported to develop after SARS-CoV-2 infection (*27*–*29*). Circulating CD69^+^CD103^−^ T cells, which exhibited lung homing potential, have been implicated in the acute pathogenesis of COVID-19 (*30*). It is thus possible that the same subset of respiratory T cells may play a role in lung pathology during both acute infection and PASC. In addition, recent reports have found that a portion of non–SARS-CoV-2–exposed individuals have preexisting SARS-CoV-2 cross-reactive T cells, which likely arose after the exposure of human endemic coronavirus (HCoV) (*31*, *32*). Although we did not detect significant levels of SARS-CoV-2–reactive T cells in aged healthy controls, it remains possible that aged individuals may exhibit increased accumulation of SARS-CoV-2 cross-reactive HCoV-specific T cells due to previous HCoV infections compared with those of young adults. Whether those cross-reactive T cells participate in the immune protection against SARS-CoV-2 reinfection and/or contribute to the development of chronic lung sequelae in COVID-19 convalescents require future investigation.

Besides antigen-dependent killing of CD8^+^ T cells, a CXCR6^hi^ tissue–resident CD8^+^ T cell population has been suggested to cause liver damage through adenosine 5′-triphosphate (ATP)–dependent autoaggression during NASH (*24*). The current study similarly identified a CXCR6^hi^ tissue-resident effector-like T cell subset at relatively high levels in the respiratory tract of COVID-19 convalescents. These cells express abundant effector, inflammatory, and tissue-destructing gene programs, supporting their potential as contributors to the persistent respiratory tissue pathology and fibrosis after resolution of acute COVID-19 infection. Whether their pathogenic function is independent of antigenic signals in the respiratory tract, as those of the cells in the liver (*24*), warrants future investigation. Nevertheless, we suggest that distinct CD8^+^ T cell populations with differential pathogenic potential are present in the respiratory tract after SARS-CoV-2 infection. Thus, it would be extremely important to engage the right subset of respiratory CD8^+^ T cells during potential mucosal vaccination to harness the power of T_RM_ cells in protective immunity but avoiding the unwanted pathology.

Because of the highly invasive nature of the bronchoscopy procedure, the study was limited to a single time point in a selected small cohort of high-risk individuals after acute COVID-19 infection and a matched limited cohort of control aged healthy individuals. In addition, the recovery from pulmonary injury often requires an extended period of time in aged individuals. Therefore, the findings reported here may not be generalizable to all patients with COVID-19, such as those young convalescents experiencing PASC. Additional future studies are warranted to determine broader applicability of these observations to younger survivors of mild or severe SARS-CoV-2 infection. Furthermore, because of practical limitations, we were unable to obtain BAL samples from people who recovered from other bacterial or viral pneumonia. Therefore, our study cannot address whether the contribution of exuberant immune responses (particularly dysregulated respiratory CD8^+^ T cell responses) in pulmonary sequelae is SARS-CoV-2 infection specific or not. Nevertheless, our results have provided the basis for the identification of the etiology of chronic lung diseases induced by acute SARS-CoV-2 infection during aging, which will help in the design of future strategies to avoid immune pathology without compromising the protective local immunity against SARS-CoV-2 in the elderly.

## MATERIALS AND METHODS

### Study design

The goal of the study was to delineate the immune mechanisms underlying the development of post-acute COVID-19 lung sequelae. We have recruited a cohort of aged (>60 years old) healthy control individuals (*n* = 5) and aged individuals (*n* = 10) who have recovered from acute COVID-19 for 2 to 3 months. We performed pulmonary function test and quantitative CT analyses to determine lung physiological function and tissue pathology in the recruited cohort. We then obtained blood samples and BAL fluid from the individuals. Enzyme-linked immunosorbent assay (ELISA) and multiplex cytokine analyses were performed to determine SARS-CoV-2–specific antibody and cytokine levels in the circulation or in the respiratory tract. High-dimensional spectral flow cytometry was performed with PBMCs and BAL cells for the characterization of circulating and respiratory immune profiles in healthy controls and COVID-19 convalescents. We correlated the circulating or respiratory immune profiles with lung functional and pathological parameters to identify the potential involvement of different immune cells in post–COVID-19 lung sequelae. Last, we performed scRNA-seq to identify potential T cell populations that may contribute to the development of chronic lung sequelae after acute COVID-19. No outliers were removed.

### Ethics statement

This study was approved by Mayo Clinic Institutional Review Boards (protocol ID 20-004911). Informed consent was obtained from all enrolled individuals.

### Study cohorts

Survivors of severe COVID-19 pneumonia, defined as patients who required hospitalizations for PCR-proven COVID-19 infection with pneumonia and need at least 2 liters of supplemental oxygen to manage hypoxemic respiratory failure, and a cohort of age-matched normal controls were recruited to the study. The use of a high-flow nasal cannula (HFNC) oxygen delivery system was undertaken when hospitalized patients required a level of supplemental oxygen requirement >6 liters. The HFNC allowed delivery of oxygen at flows of 40 to 70 liters, enabling approximate FiO_2_ ranging from 0.45 to a maximum of 0.70 at the highest flow rate. Flow rate was adjusted by the bedside to attain an oxygen saturation of 88 to 92%.

The COVID-19 convalescent cohort consists of patients between the ages of 60 and 85 that had no evidence of preexisting interstitial lung disease or chronic lung disease. For both the COVID-19 and control cohort, previous lung disease was excluded through evaluation of the electronic medical records and clinical evaluation before performing bronchoscopy. Patients with a history of <10 pack-years of smoking and mild chronic obstructive pulmonary disease (COPD) with FEV1 > 80% predicted and FEV1/FVC < 0.7 were still eligible for enrollment. At the time of bronchoscopy, control individuals had to have absence of lung infiltrate, fever, or any signs or infection. Most of the controls underwent bronchoscopy for evaluation of lung nodule or focal adenopathy of indeterminate cause. The exclusion criteria for both the COVID-19 and control cohorts included the inability to provide consent to participate in the study; patient under guardianship or curatorship; preexisting chronic lung disease including interstitial lung disease, pulmonary fibrosis, or any other chronic lung disease with the exception of mild COPD as outlined in “inclusion criteria”; active cigarette smoking, vaping, or other inhalation use (former smoker providing quit >90 days before admission acceptable); and immunocompromised host status due to ongoing therapy with methotrexate, CellCept, azathioprine, prednisone dose >15 mg daily, rituximab, cyclophosphamide, or other immunosuppressive or other biologic agents. All patients with COVID-19 were enrolled for the study with bronchoscopy and BAL, acquisition of peripheral blood for PBMC, and RNA research sample as well as peripheral blood for clinical preoperative clearance laboratories, pulmonary function testing, ECG, and imaging of the chest with a noncontrast high-resolution CT scan. The COVID-19 patient cohort was enrolled within a 60- to 90-day window after onset of acute COVID-19 infection, the onset of which was defined as the day when the PCR COVID-19 swab was recorded as positive.

### CALIPER analysis of CT image

The analysis of CT image by CALIPER was performed as previously reported (*14*). Initial data processing steps involved extraction of the lung from the surrounding thoracic structures and segmentation into upper, middle, and lower zones. Lung segmentation was performed with an adaptive density-based morphologic approach, whereas airway segmentation involved iterative three-dimensional region growing, density thresholding, and connected components analysis. Parenchymal tissue type classification was applied to 15 × 15 × 15 voxel volume units using texture analysis, computer vision–based image understanding of volumetric histogram signature mapping features, and three-dimensional morphology. The CALIPER tool was trained by subspecialty thoracic radiologist consensus assessment of pathologically confirmed datasets (*14*, *33*).

### Human pulmonary function testing

Pulmonary function testing was performed on COVID-19 and control cohort. All individuals underwent measurement of FVC and FEV1 as well as measurement of DLCO. In addition, when possible and tolerated, lung volumes were also determined including TLC. In all individuals, spirometry was performed in the institutional pulmonary function laboratory at Mayo Clinic in accordance with American Thoracic Society (ATS) guidelines (*34*).

### Bronchoscopy and BAL collection

Fiber-optic bronchoscopy and BAL were performed using moderate conscious sedation using standard clinical procedural guidelines in an outpatient bronchoscopy suite. Conscious sedation was administered in accordance with hospital policies, and a suitably trained registered nurse provided monitoring throughout the procedure. The scope was wedged into an affected segment predetermined by review of CT scan. About 100 to 200 ml of saline were instilled in 20-ml aliquots until 60 ml of lavage fluid was obtained. The specimen was placed on ice and immediately hand carried to laboratory for analysis. The fluid collected was placed on ice and transferred immediately to the laboratory for processing.

### Mouse infection and lung function measurement

Female aged mice were received at 20 to 21 months of age from the National Institute of Aging and maintained in the same specific pathogen–free conditions for at least 1 month before infection. All mice were used under conditions fully reviewed and approved by the Institutional Animal Care and Use Committee guidelines at the Mayo Clinic (Rochester, MN). For primary influenza virus infection, influenza A/PR8/34 strain was diluted in fetal bovine serum (FBS)–free Dulbecco’s modified Eagle’s medium (DMEM) (Corning) on ice and inoculated in anesthetized mice through intranasal route as described before (*11*).

Lung function measurements using FOT and the resulting parameters have been previously described (*35*). In brief, animals were anesthetized with an overdose of ketamine/xylazine and tracheostomized with a blunt 18-gauge canula. Animals were connected to the computer-controlled piston (flexiVent), and forced oscillation mechanics were performed under tidal breathing conditions described in (*35*) with a positive-end expiratory pressure of 3 cm H_2_O. The measurements were repeated following thorough recruitment of closed airways (two maneuvers rapidly delivering TLC of air and sustaining the required pressure for several seconds, mimicking holding of a deep breath). Each animal’s basal conditions were normalized to their own maximal capacity. Measurement of these parameters before and after lung inflation allows for determination of large and small airway dysfunction under tidal (baseline) breathing conditions. Only measurements that satisfied the constant-phase model fits were used (>90% threshold determined by software). After this procedure, mice had a heart rate of ~60 beats per minute, indicating that measurements were done on live individuals. Constant-phase model equation was described as before (*36*)

$$Z(f)=\text{Raw}+i2\pi \mathrm{fI}+(G_{\text{ti}}–{\mathrm{iH}}_{\text{ti}})/{\left(2\pi f\right)}^{\alpha }$$ (1)

for Raw equal to the Newtonian or airway resistance (*R*_N_), and *G* and *H* are frequency-independent measures of tissue (ti) resistance and elastance, respectively. *I* is airway inertance, and *i* is the square root of −1, whereas *a* is determined by *G* and *H* [*a* = (2/π) arctan(*H*_ti_/*G*_ti_)].

Forced oscillatory lung mechanics can be further analyzed to fit a single-compartment lung model with constant-phase tissue impedance at each frequency applied to the lung. When this pulmonary input impedance (*Z*_in_) data are solved, we are left with a real part of *Z*_in_ indicative of the resistance of airways of increasing size across the broadband signal and a summed imaginary part of *Z*_in_ called the reactance. Input impedance equation was calculated as reported (*37*) and displayed as mean percentage ± SEM as either raw data (fig. S9) or a percentage of maximal lung capacity under tidal breathing conditions (Fig. 3D).

Input impedance equation

$$Z_{\text{in}}(ƒ)=R_{N}(ƒ)+i2\pi ƒI+(G_{\text{ti}}–{\mathrm{iH}}_{\text{ti}})/{\left(2\pi ƒ\right)}^{a}$$ (2)

### Single-cell purification

Before the PBMC isolation, 500 μl of blood was saved for innate cell analysis. Plasma was isolated from whole blood by centrifuging at 1600 rpm, room temperature (RT), for 10 min. After plasma isolation, leftover blood was mixed with phosphate-buffered saline (PBS) and then gently put over on Ficoll-Paque (Cytiva) in a 50-ml tube. Buffy coat generated by centrifuging at 400*g* for 40 min at RT was collected. For single-cell purification from BAL, BAL was filtered with a 70-μm cell strainer (Falcon) and then centrifuged at 300*g* for 10 min at 4°C. Supernatant was collected for multiplex assay and ELISA. The cells were collected for flow cytometry analysis and scRNA-seq.

### Cytokine and chemokine measurements

BAL and plasma were isolated as above, and aliquots were stored at −80°C. BAL was further concentrated for 20× using 3-kDa Amicon Ultra-15 Centrifugal Filter Unit (Merck). BAL and plasma were shipped to Eve Technologies (Calgary, Alberta, Canada) on dry ice, and levels of cytokines and chemokines were measured using the Human Cytokine Array/Chemokine Array 71-403 Plex Panel (HD71).

### Flow cytometry analysis

BAL cells or PBMCs were stained with antibodies as listed in data file S3. The high-parameter analysis was performed using Cytek Aurora (Cytek Biosciences). Briefly, cells were washed with fluorescence-activated cell sorting (FACS) buffer (2% of FBS and 0.1% of NaN_3_ in PBS) and incubated with antibodies of surface markers in the dark for 30 min at 4°C. After wash with PBS, cells were resuspended with Zombie dye (BioLegend) and incubated at RT for 15 min. For staining transcriptional factors, cells were fixed, permeabilized, and stained with a Foxp3 transcription factor staining kit (Tonbo) following the manufacturer’s manual. For cytokine staining, cells were restimulated with predicted overlapping peptide pools against all SARS-CoV-2 proteins. BAL cells or PBMCs were stimulated by peptide pools for 6 hours. The cells were washed with FACS buffer and then stained with surface markers. After incubation with viability dye, cells were fixed with fix buffer (BioLegend) at RT for 30 min. The cells were permeabilized with intracellular staining kit (BioLegend) and then stained with cytokine antibodies at RT for 1 hour. To detect RBD-specific B cells, recombinant RBD proteins, which were generated from J.T.’s laboratory, were incubated with the cells at 4°C for 30 min. RBD-PE (phycoerythrin) and RBD-APC (allophycocyanin) double-positive B cells were identified as RBD^+^ B cells. The same cell number of clean samples, which excluded debris, doublets, and dead cells, was exported and then merged as a concatenated file. Uniform manifold approximation and projection (UMAP) was generated with the concatenated file and then analyzed for further parameters. Generation of UMAP was performed by FlowJo (BD) plug-in packages.

### Antibody production against SARS-CoV-2

General ELISA method has been previously described (*38*). Briefly, recombinant SARS-CoV-2 proteins including RBD (Sino Biological), spike S1 (S1) (Sino Biological), or nucleocapsid protein (N) (GenScript) were precoated to 96-well plates overnight at 4°C. The following day, plates were washed with wash buffer (0.05% Tween 20 in PBS) and then blocked with 3% of milk in 0.05% PBS–Tween 20 for 1 hour at RT. Plasma or 20× concentrated BAL from control or convalescent patients was diluted in 1% of milk in PBS–Tween 20 starting at a 1:1 or 1:3 dilution, respectively, and then serially diluted by 3. The diluted BAL or plasma was added to the plate and incubated for 2 hours at RT. After washing with wash buffer, secondary antibodies diluted in 1% of milk in 0.05% PBS–Tween 20 were added to the plate and then incubated for 1.5 hours at RT. Secondary antibodies including anti-human IgG (Sigma-Aldrich), IgM (Sigma-Aldrich), or IgA (Hybridoma Reagent Laboratory) were diluted at 1:5000, 1:10,000, or 1:1000, respectively. Plates were intensively washed and then developed with 3,3’,5,5’ tetramethyl benzidine (TMB) buffer (BioLegend) for 10 to 15 min at RT. Sulfuric acid (2 M) was used as STOP buffer. Plates were read on a microplate reader (Molecular Devices) at 450 nm with SoftMax Pro Software. The endpoint titers were displayed as a dot for each patient. For correlation matrix analysis, the endpoint titers were cut off at an optical density (OD) of 0.2.

### T cell purification and scRNA-seq/TCR-seq assay

CD3^+^ T cells were purified from PBMC and BAL cells with the StraightFrom Whole Blood CD3 MicroBeads (Miltenyi Biotec). The purified T cells were stained with PE-Cy7 anti-human CD3 antibody for the purification efficiency check. The purified PBMC or BAL T cells were labeled with TotalSeq-C0080 anti-human CD8a antibody, TotalSeq-C0072 anti-human CD4 antibody, TotalSeq-C0090 mouse IgG1, κ isotype control antibody, and unique TotalSeq-C anti-human hashtag antibodies (BioLegend) and then mixed at a ratio of 1:1.

To facilitate the single-cell gene expression (GEX), Feature Barcode technology for cell surface protein, and TCR-seq profiling from the purified T cells, 10× 5′ Library & Gel Bead Kit v1.1 was used. A total of 10,000 cells were targeted for single-cell libraries preparation as per the manufacturer’s instructions (10x Genomics).

### Single-cell transcriptome analysis

scRNA-seq and TCR-seq data were aligned and quantified using 10x Genomics Cell Ranger Software Suite (v4.0.0) against their corresponding human reference genome (GRCh38) downloaded from 10x Genomics website. Using default settings in Seurat 4.0.1 package, the filtered transcriptome data were then normalized (RNA expression by a factor of 10,000 with log-transformed, cell surface protein of Feature Barcode by a centered log ratio). Then, PBMC T cells and BAL T cells were separated on the basis of the unique hashtag antibodies, and the threshold for classification was used as 0.99. The threshold of percent.MT is 10 to exclude dead cells. The RNA expression data were then further scaled based on regressing the number of unique molecular identifiers (UMIs) detected and the percentage of mitochondrial counts per cell. Principal components analysis (PCA) was performed using the top variable genes. FindNeighbors and FindClusters functions were applied for cell clustering in Seurat for either dataset. The CD8^+^ T cell and CD4^+^ T cell clusters were verified by the cell surface protein expression level based on feature barcoding antibodies. Differential gene expression analysis was performed by the function of FindAllMarkers from Seurat with model-based analysis of single-cell transcriptomics (MAST) test, and gene set enrichment analysis (GSEA) analysis is based on the results of FindAllMarkers with the package of clusterProfiler (*39*). AddModuleScore function was applied for analyzing cell population signatures, and the tissue-resident, effector, and memory gene sets used were based on published datasets described before (*24*, *40*, *41*). Pseudotime analysis was performed using SeuratWrappers and Monocle 3 combination, based on the Seurat processed analysis at the single-cell level. Age-unmatched BAL T cells were extracted from published dataset GSE151928 (*42*), and recovery T cells were set as reference dataset during integrating T cells from healthy donor.

### Single-cell TCR-seq analysis

Joint analysis of single-cell transcriptomes and TCR repertoires was performed by the function of combineExpression from package of scRepertoire v1.14.0 (*43*). The abundance of cloneTypes was defined on the basis of its frequency (0 < Rare ≤ 1, 1 < Small ≤ 5, 5 < Medium ≤ 20, 20 < Large ≤ 100, 100 < Hyperexpanded ≤ 1000). Clone size was depicted on UMAP (per cell) by the function of occupiedscRepertoire from package of scRepertoire v1.14.0. Indicated clonotypes were depicted on UMAP (per cell) by the function of highlightClonotypes. Volcano plots of differentially expressed genes (DEG) were generated by comparing cells with cloneTypes frequency ≤ 5 and cloneTypes frequency > 5 by the function of FindAllMarkers from Seurat with MAST test. Gene Expression Omnibus accession number of this study is GSE176201.

### SARS-CoV-2 gene detection

Total RNA was extracted from whole BAL cells or PBMCs with a Total RNA Purification kit (Sigma-Aldrich) according to the manufacturer’s instructions. Complementary DNA (cDNA) was synthesized using random primers (Invitrogen) and M-MLV reverse transcriptase (Invitrogen). RT-PCR was performed using Fast SYBR Green PCR Master Mix (Applied Biosystems) with primers nCoV_N1 (forward, GACCCCAAAATCAGCGAAAT; reverse, TCTGGTTACTGCCAGTTGAATCTG) and human HPRT (forward, GACCAGTCAACAGGGGACAT; reverse, CTGCATTGTTTTGCCAGTGT) in QuantStudio 3 (Applied Bioscience). Data were generated by the comparative threshold cycle (Δ*C*_T_) method by normalizing to HPRT. For positive or negative control of SARS-CoV-2 gene detection, RNAs were extracted from noninfected healthy human alveolar macrophages (AMs) or SARS-CoV-2–infected AMs [one multiplicity of infection (MOI)].

### Statistical analysis

To compare between two sample groups (CON and CVD), Mann-Whitney test was applied for unpaired comparisons. For analysis between several groups, one- or two-way analysis of variance (ANOVA) was performed. Correlations were assessed by the Pearson correlation coefficient running under R in RStudio (1.4). All statistical tests were performed using GraphPad Prism version 9 (GraphPad Software) or R (version 4.0.3).

## Supplementary Material

20210930-1

## Notes

View/request a protocol for this paper from *Bio-protocol*.
