# Supplementary material for: Immune signatures underlying post-acute COVID-19 lung sequelae
Source: Sci Immunol. 2021 Nov 12;6(65):eabk1741. doi: 10.1126/sciimmunol.abk1741 (PMC8763087; doi:10.1126/sciimmunol.abk1741)
Supplement: 20210930-1 [file sciimmunol.abk1741.v1.pdf]

Science Immunology

Cite as: I. S. Cheon *et al.*, *Sci. Immunol.* 10.1126/sciimmunol.abk1741 (2021).

CORONAVIRUS

Immune signatures underlying post-acute COVID-19 lung sequelae

Cheon IS[1,2*], Li C[1,2*], Son YM[1,2*], Goplen NP[1,2], Wu Y[1,2], Cassmann T[1,2], Wang Z[1,2], Wei X[1,2], Tang J[1,2], Li Y[3], Marlow H[1], Hughes S[1], Hammel L[1], Cox TM[1], Goddery E[2], Ayasoufi K[2], Weiskopf D[4], Boonyaratanakornkit J[5], Dong H[2], Li H[6], Chakraborty R[2,7], Johnson AJ[2], Edell E[1], Taylor JJ[5], Kaplan MH[8], Sette A[4,9], Bartholmai BJ[10], Kern R[1], Vassallo R[1, 11#] and Sun J[1,2,11-14 #†]

1. Division of Pulmonary and Critical Medicine, Department of Medicine, Mayo Clinic, Rochester, MN 55905, USA 2. Department of Immunology, Mayo Clinic, Rochester, MN 55905, USA 3. Division of Biomedical Statistics and Informatics, Mayo Clinic, Rochester, MN 55905, USA 4. Center for Infectious Disease and Vaccine Research, La Jolla Institute for Immunology (LJI), La Jolla, CA 92037, USA 5. Vaccine and Infectious Disease Division, Fred Hutchinson Cancer Research Center, Seattle, WA 98109, USA 6. Department of Molecular Pharmacology & Experimental Therapeutics. Mayo Clinic, Rochester, MN 55905, USA 7. Department of Pediatrics and Adolescent Medicine, Mayo Clinic, Rochester, MN 55905, USA 8. Department of Microbiology and Immunology, Indiana University of School of Medicine, Indianapolis, IN 46202, USA 9. Department of Medicine, Division of Infectious Diseases and Global Public Health, University of California, San Diego (UCSD), La Jolla, CA 92037, USA 10. Department of Radiology, Mayo Clinic, Rochester, MN 5590, USA 11. Department of Physiology and Biomedical Engineering, Mayo Clinic, Rochester, MN 55905, USA 12. The Robert and Arlene Kogod Center on Aging, Mayo Clinic, Rochester, MN 55905, USA 13. Carter Immunology Center, University of Virginia, Charlottesville, VA 22908, USA 14. Division of Infectious Disease and International Health, Department of Medicine, University of Virginia, Charlottesville, VA 22908, USA.

*, Co-first authors

#, Co-senior authors

†**Corresponding author. Email: sun.jie@mayo.edu, js6re@virginia.edu**

Severe COVID-19 pneumonia survivors often exhibit long-term pulmonary sequalae, but the underlying mechanisms or associated local and systemic immune correlates are not known. Here, we have performed high-dimensional characterization of the pathophysiological and immune traits of aged COVID-19 convalescents, and correlated the local and systemic immune profiles with pulmonary function and lung imaging. We found that chronic lung impairment was accompanied by persistent respiratory immune alterations. We showed that functional SARS-CoV-2-specific memory T and B cells were enriched at the site of infection compared to those of blood. Detailed evaluation of the lung immune compartment revealed dysregulated respiratory CD8$^+$ T cell responses were associated with the impaired lung function following acute COVID-19. Single cell transcriptomic analysis identified the potential pathogenic subsets of respiratory CD8$^+$ T cells contributing to persistent tissue conditions following COVID-19. Our results have revealed pathophysiological and immune traits that may support the development of lung sequelae following SARS-CoV-2 pneumonia in older individuals, with implications for the treatment of chronic COVID-19 symptoms.

INTRODUCTION

The main determinant of clinical outcomes in individuals with Severe Acute Respiratory Syndrome Coronavirus 2 (SARS-CoV-2) infection is age, with the majority of morbidity and mortality occurring in the elderly (*1*, *2*). Beyond the acute morbidity induced by SARS-CoV-2 infection, data have shown that patients with severe acute COVID-19 illness are at highest risk of developing chronic pulmonary sequelae and persistent chest imaging abnormalities (*3*–*6*). However, the mechanisms underlying the development of chronic lung sequelae following acute SARS-CoV-2 infection remain elusive. As the numbers of patients with symptoms persisting after resolution of acute illness rises (so-called chronic COVID-19), there is an urgent need to identify the cellular, molecular and pathophysiological characteristics of chronic lung sequelae in COVID-19 convalescents. Elucidation of specific mechanisms by which chronic lung injury develops is essential for the development of future preventive and/or therapeutic interventions for chronic COVID-19 symptoms.

T cells and B cells can form long-lived immunological memory after the clearance of primary viral infection to protect the host from reinfection of the same or related viruses (*7*). Memory lymphocytes are generally divided into circulating memory cells that patrol the body and tissue-resident memory cells that reside in the peripheral non-lymphoid tissue (*7*, *8*). Tissue-resident T (T_{RM}) and B (B_{RM}) cells that reside within the respiratory tract provide immediate and superior immunity against viral re-infections (*9*), but dysregulated lung-resident T cell responses have also been linked with chronic lung inflammation, pathology and fibrosis following

respiratory viral infection, particularly in older hosts (*10*, *11*). Of note, evidence has suggested that antigen-specific B cell and T cell memory is formed and can persist in the circulation for more than 6 months following SARS-CoV-2 infection (*12*, *13*). However, tissue-specific memory responses in the respiratory tract have not been previously characterized following SARS-CoV-2 infection. The current report describes detailed characterization of circulating and respiratory immune profiles and quantitative lung functional and pathological parameters in a cohort of aged COVID-19 convalescents. Our results suggest that dysregulated pulmonary immune responses, particularly exuberant responses of certain respiratory CD8$^+$ T cell subsets, may contribute to the development of chronic lung sequelae following resolution of acute COVID-19 infection in aged individuals.

RESULTS

Pathophysiological characterization of lung sequelae post-COVID-19

Aged hosts develop chronic lung inflammatory and fibrotic sequelae following viral pneumonia in an animal model (*11*). We hypothesized that aged individuals who survived moderate to severe acute COVID-19 infection may experience persistent lung inflammation and fibrosis, and impaired lung function. To this end, we recruited a cohort of aged healthy control and COVID-19 convalescents (all >60 years of age) discharged from hospital and evaluated 60 to 90 days following onset of SARS-CoV-2 infection (Fig. 1A, B and Fig. S1 A). Most of these subjects were unvaccinated (Fig. S1A and Data file S1). There were two exceptions: one in control (CON) who completed two doses mRNA vaccination 4 weeks before the bronchoscopy procedure, and one in the COVID-19 convalescent group (CVD) who completed two doses of mRNA vaccination one week before the bronchoscopy procedure. The COVID-19 convalescent who had completed vaccination remained SARS-CoV-2 PCR$^+$ but showed no signs of active infection at the time of enrollment in the study. However, this was not confirmed by SARS-CoV-2 N1 gene RT-PCR in the laboratory as we found that none of the COVID-19 convalescents harbored SARS-CoV-2 N1 gene in the bronchoalveolar lavage fluid (BAL) (Fig. S1 B). There were no pre-existing lung conditions prior to SARS-CoV-2 infection in these individuals, and so all abnormalities detected on imaging or lung function testing were likely attributable to SARS-CoV-2 infection. All COVID-19 convalescents had persistent pulmonary and extrapulmonary symptoms at the time of study enrollment (Fig. 1A, B and Data file S1), suggesting that they were experiencing post-acute sequelae of COVID-19 (PASC). We obtained BAL and blood from these individuals and performed high dimensional spectral flow cytometry and single cell RNA sequencing (scRNA-seq) on BAL and blood immune cells (Fig. 1A). We also conducted pulmonary function tests (Spirometry and lung diffusion capacity) and quantitative computerized tomography (CT) using the automated Computer-Aided Lung Informatics for Pathology Evaluation and Rating (CALIPER) technique (*14*) (Fig. 1C and Fig. S1 C-E).

Lungs of aged COVID-19 convalescents showed heterogeneous imaging features suggestive of ongoing inflammation and fibrosis. While a couple of individuals had almost full lung recovery from the infection (CVD Recovered), most patients (>70%) exhibited moderate (CVD Moderate) to severe (CVD Severe) CT abnormalities (yellow and orange regions, Fig. 1C and Fig. S1 D, E) and impaired pulmonary function more than two months after hospital discharge (Fig. 1B, Fig. S1 C, and Data file S1). Lung function parameters were abnormal in several of the COVID-19 cohort, with physiologic evidence of a pulmonary parenchymal restrictive abnormality (reduced TLC (total lung capacity) and FVC (forced vital capacity) evident in several individuals. Pulmonary function parameters including FEV1 (forced expiratory volume in 1 s), FVC and DLCO (diffusion capacity for carbon monoxide) were inversely correlated with the extent of lung ground glass opacification (GGO, yellow regions), reticular densities and consolidation (orange regions) and/or vessel related structures in the CT image (Fig. 1D, E), and positively correlated with residual normal or relatively spared lung regions (total volume or % of light and dark green regions). These data suggest that the extent of tissue pathology following acute COVID-19 determines the pulmonary gas-exchange function of aged COVID-19 convalescents. Notably, the majority of the aged COVID-19 convalescents showed decreased normal lung regions, and increased tissue exhibiting GGO, reticular and vascular related structures, accompanied by decreased FEV1, FVC, DLCO and other lung functional parameters (Fig. 1E, Fig. S1 C-E and Data files S1, S2). Taken together, our detailed characterization of lung pathophysiological parameters demonstrates that acute SARS-CoV-2 infection resulted in long-term lung imaging and functional sequelae in a cohort of aged individuals.

Respiratory and systemic immune traits of aged COVID-19 convalescents

The levels of most proinflammatory cytokines were comparable in the blood or respiratory tract of the control and COVID-19 convalescents (Figure S2), which is in contrast to what was reported during the acute stage of COVID-19 (*15*, *16*) and indicates that the acute inflammatory responses had largely resolved in the convalescent stage. To determine the systemic and pulmonary immune cell profile, high dimensional flow cytometric analysis was performed on circulating and respiratory immune cells in control and aged COVID-19 convalescents (Fig. S3, S4). While most of immune cell types (except NK cells) were comparable in the circulation between control and COVID-19 convalescents (Fig. 2A), there was an increase in the frequency of γ/δ T cells, B cells and CD8$^+$ T cells within the respiratory compartment of the COVID-19

convalescent cohort compared to those of control (Fig. 2B). Of note, among the subtypes of increased BAL immune cells, $CD8^+$ T cells appeared to be the most abundant population (3.54 to 12.29%) (Fig. 2B). Thus, SARS-CoV-2 infection leads to persistent alterations of respiratory immune profiles at the convalescent stage.

Characterization of respiratory immune memory following SARS-CoV-2 infection

Sustained circulating antibody and memory lymphocyte responses have been detected months after acute SARS-CoV-2 infection (*17*). However, the characteristics of antibody and memory lymphocyte responses in the lower respiratory tract have not been reported. As expected, elevated IgG and IgA antibody responses against SARS-CoV-2 protein N, S1 and RBD (S receptor binding domain) were detected in both plasma and BAL of COVID-19 convalescents compared to those of control; evidence of humoral S1 and RBD IgG responses were detected in the one control subject that had received COVID-19 vaccination (Fig. S5 A). SARS-CoV-2-specific IgM responses were less distinct between healthy controls and the COVID-19 convalescent group (Fig. S5 B). RBD-specific B cells were detected in both PBMC and BAL, and there was a trend of increase in the percentages of RBD-specific B cells within the total BAL B cell population, suggesting that mucosal B cell pool is likely concentrated with antigen-specific memory B cells at the site of infection (Fig. 2C). Compared to circulating RBD-specific B cells, BAL RBD-specific B cells had increased CD69 and CD27 (Fig. 2D & Fig. S4), but diminished IgD and CD38, indicating that these SARS-CoV-2-specific B cells have a resident memory phenotype. The levels of BAL SARS-CoV-2-specific IgG responses were correlated with local $CD4^+$ T cells levels, particularly the abundance of tissue resident-like $CD69^+$ $CD4^+$ T cells (Fig. S5 C, D), indicating that $CD4^+$ T cells may be required for sustaining respiratory Ab responses at the memory stage following the recovery of primary respiratory viral infection, as was observed in animal models (*18*, *19*).

We next analyzed the presence of different $CD8^+$ T cell population, including naïve, effector memory (T_{EM}), central memory (T_{CM}), effector memory T cells re-expressing CD45RA (T_{EMRA}) and T_{RM} cells in the BAL and PBMC of control and COVID-19 convalescents (Fig. S6 A-D). $CD69^+$ T_{RM}-like cell (including $CD69^+CD103^+$ conventional T_{RM} and $CD69^+CD103^-$ T cells) presence was increased in the respiratory tract of COVID-19 convalescents compared to those of aged controls (Fig. 2E and Fig. S6 E). Within the BAL $CD8^+$ T cell population, there appeared to be an enrichment of $CD69^+CD103^-$ T cells in COVID-19 convalescents (Fig. 2F). Compared to $CD103^+$ T_{RM} cells, $CD69^+CD103^-$ T cells expressed higher levels of EOMES, Granzyme B, Granzyme K, CXCR4 and KLRG1 (Fig. S6 F). Following stimulation with a pool of predicted overlapping SARS-CoV-2 peptides (*20*), there appeared to be higher percentages of cytokine (IFN-γ or TNF)-producing cells in the BAL $CD8^+$ T cell population compared to those of peripheral blood $CD8^+$ T cells (Fig. 2G and Fig. S6 G), and the cytokine production between $CD69^+CD103^+$ T_{RM} and $CD69^+CD103^-$ T cells were comparable (Fig. S6 H). Notably, there were higher percentages of $CD8^+$ T cells capable of producing multiple cytokines in the respiratory tract than those of cells in the circulation (Fig. S6 I), suggesting that SARS-CoV-2 infection results in polyfunctional memory $CD8^+$ T cell generation in the respiratory mucosa.

Examination of $CD4^+$ T cells demonstrated that percentages of $CD4^+$ T_{CM} and follicular helper (T_{FH})-like cells were elevated in the respiratory tract of COVID-19 convalescents compared to those of healthy control (Fig. 2 H and Fig. S7 A-D). In COVID-19 convalescents, there was an enrichment of SARS-CoV-2-specific $CD4^+$ T cell presence in the respiratory tract, and BAL memory $CD4^+$ T cells were more polyfunctional than those of blood memory $CD4^+$ T cells (Fig. 2I and Fig. S7 E-G). Together, these data suggest that functional B, $CD4^+$ and $CD8^+$ memory cells are present in the respiratory tract following recovery from acute COVID-19 pneumonia, and these memory T and B lymphocytes showed enhanced tissue-residency characteristics compared to those of circulating memory counterparts.

Dysregulated $CD8^+$ T cell responses are associated with impaired lung function following viral pneumonia

We examined the association of lung function and imaging abnormalities with respiratory and circulating immune profiles. The levels of circulating mast cells were negatively, while γ/δ T cells were positively, correlated with better lung function in COVID-19 convalescents (Fig. 3A). Circulating B cell levels appeared to be positively correlated with lung pathological parameters (Fig. 3A). In the respiratory tract, the levels of eosinophils, DCs, pDCs, $CD4^+$ T cells and B cells were negatively correlated with lung function (FEV1, FVC) or percentages of normal or relatively preserved parenchymal imaging lung characteristics (percentage of mild low attenuation and spared lung on imaging, Fig. 3B). Notably, respiratory $CD8^+$ T cells were negatively correlated with lung function parameters and positively correlated with lung pathological parameters, suggesting a potential role of respiratory $CD8^+$ T cells in contributing to the development of lung sequelae following acute COVID-19 (Fig. 3B).

$CD8^+$ T_{RM}-like cells, particularly $CD69^+CD103^-$ T cells were negatively correlated with lung function (DLCO and FEV1) in combined healthy control and COVID-19 convalescent groups (Fig. 3C) or in COVID-19 convalescent group alone (Fig. S8). Notably, our cohort contained relatively fewer female individuals as majority of severe COVID-19 cases occur in male sex. Since sex differences and/or vaccine administration may alter the dynamics of T cell responses and tissue recovery, we

have also performed correlation analysis of respiratory CD8⁺ T cell levels with lung function after the exclusion of vaccinated and/or female subjects (Fig. S9). Notably, BAL CD69⁺CD103⁻ T cell levels remained negatively correlated with lung functional parameters in this setting (Fig. S9). Taken together, these data indicate that dysregulated CD8⁺ T_{RM} cell composition or responses in aged COVID-19 convalescents may contribute to the impaired lung function and/or development of chronic lung sequelae.

Our data are in line with the findings that the depletion of CD8⁺ T cells led to decreased chronic inflammation and lung pathology following influenza viral pneumonia during aging (*11*), although the roles of CD8⁺ T cells in lung function following acute pneumonia were not studied. To this end, we utilized the reported murine model of chronic lung sequalae following influenza pneumonia during aging (*11*). We infected aged mice (20-22 months old) with influenza A virus (PR8 strain) and then depleted CD8⁺ T cells at 3 weeks following infection, which mice have recovered from the primary infection. 4 - 5 weeks later, we measured lung function using forced oscillation technique (FOT) by flexiVent. FOT allows for the distinction of mechanics arising from small (G_{ti}) and large (Rn) airways. With respect to maximum lung capacity, infection increased the resistance to airflow in the small, but not large airways at baseline. This dysfunction in the small airways was rescued by the depletion of CD8⁺ T cells in aged mice following the recovery of primary viral pneumonia (Fig. S10). To ascertain whether terminal airways were affected, the extent of dysfunction throughout the airway tree was analyzed from input impedances (Z_{rs}) of the FOT data. Under baseline breathing conditions, aged naïve mice utilized 75-80% of their lung capacity in both small and large airways. Infection reduced this utilization by a further 5-10% primarily in smaller airways. However, in CD8⁺ T cell-depleted animals, the smallest airways showed significant functional recovery following infection (Fig. 3D, S10), indicating increased alveoli recruitment during tidal breathing. Together, this indicates there is long-term infection-induced dysfunction in the alveoli driven by the presence of CD8⁺ T cells in aged hosts following viral pneumonia.

Identification of potentially pathogenic respiratory CD8⁺ T cell subsets in aged COVID-19 convalescents

To gain further insights on the cellular and molecular characteristics of the respiratory and circulating memory T cell responses following SARS-CoV-2 infection, we performed scRNA-seq and profiled 27,167 BAL and blood T cells from six COVID-19 convalescents. Nine major T cell clusters across BAL and PBMC (Fig.S11 A, B), and twelve subclusters of PBMC and BAL CD4⁺ T cells were identified (Fig. S11 C, D). BAL CD4⁺ T cells showed distinct features of gene expression and tissue resident gene programs with four major sub-clusters identified (Fig. S11 E-J). Compared with age-matched or unmatched healthy controls, aged COVID-19 convalescents had increased presence of cluster 2 CD4⁺ T cells, which expressed genes associated with myeloid cell inflammation including Lyz, S100 A8 and S100 A9 (*21*) (Fig. S11 K-M). TCR sequencing also revealed the presence of clonally expanded T cells, which potentially represented those SARS-CoV-2-specific T cells, within the circulation and BAL CD4⁺ T cell populations (Fig. S12).

14 sub-clusters of blood and respiratory CD8⁺ T cell population were identified following scRNAseq (Fig. S13A). TCR sequencing identified the presence of clonally expanded T cells within the circulating and BAL CD8⁺ T cell populations (Fig. S13 B-E). Furthermore, those clonally expanded T cells had higher levels of effector or cytotoxic molecules, supporting the view that those were likely antigen-experienced T cells (Fig. S13 F, G). After excluding the cycling cell population, 3,254 BAL CD8 T cells were further subclustered into three major clusters: cluster 0 (ITGAE (CD103)^{hi} ZNF683 (HOBIT)^{hi}), comprised of conventional CD103⁺ T_{RM} cells; cluster 1(EOMES^{hi}), comprised of CD69⁺CD103$^{-/low}$ T_{RM} cells; and cluster 2 CD8⁺ T cells expressing high levels CXCR6, PRF1 and TNF (Fig. 4A, B, S14). Compared with circulating CD8⁺ T cells, respiratory CD8⁺ T cells had distinct features of gene expression and tissue-resident gene programs (Fig. S14 A-C). Pseudotime analysis by Monocle 3 suggested that cluster 2 was less differentiated, while the conventional CD103⁺ cells were most differentiated clusters (Fig. S14 G). Notably, BAL CD103^{low} T_{RM} cells appeared to have highest level of shared TCR sequences with PBMC CD8⁺ T cells (Fig. S15). Further analysis identified that even though all three clusters exhibited a similar tissue residency gene program and down-regulation of genes associated with circulation (Fig. 4C left, S14 H), cluster 2 showed a lower T memory cell, but higher T effector cell, gene signature (Fig. 4 C middle and right). Clusters 1 and 0 shared comparable T memory cell signatures, but the TCR signaling gene set was enriched in cluster 1 (Fig. S14 I), indicating that CD103⁺ T_{RM} cells (cluster 0) had a more quiescent phenotype than CD69⁺CD103⁻ T cells (cluster 1). Cluster 1 CD103$^{-/low}$ T_{RM} cells also expressed higher levels of NKG7, a cytotoxic molecule that can promote lethal inflammation following infection (*22*); and Granzyme K, an inflammatory granzyme that promotes fibroblast activation (*23*) (Fig. 4B, S6 F, S14 F). Together, these data suggest that the cluster 1 CD103$^{-/low}$ T_{RM} cells possess higher pathogenic potential than those of cluster 0, the conventional T_{RM} cells.

A population of CXCR6^{hi} pathogenic tissue-resident CD8⁺ T cells were recently identified as a driver of tissue damage during nonalcoholic steatohepatitis (NASH) (*24*). Indeed, cluster 2 CXCR6^{hi} respiratory CD8⁺ T cells in COVID-19 convalescents were enriched with the liver pathogenic CXCR6^{hi} gene signature compared to those of cluster 0 (Fig. 4D, S14 J). Furthermore, compared with cluster 0 and 1, cluster 2

CXCR6^{hi} CD8$^+$ T cells showed enriched gene programs associated with tissue-aggressive features, which include allograph rejection, asthma and type I diabetes related genes (Fig. 4E). Notably, compared with both age-matched or unmatched healthy controls, aged COVID-19 convalescents had increased presence of cluster 2 CXCR6^{hi} CD8$^+$ T cells within the BAL CD8$^+$ T cell population (Fig. 4F, S14 K). Additionally, the abundance of cluster 2 CXCR6^{hi} CD8$^+$ T cells in BAL was negatively correlated with lung gas exchange function and positively associated with the extent of lung GGO and reticular consolidation (Fig. 4G). Taken together, our flow cytometric and scRNA-seq analysis have revealed marked heterogeneity in the respiratory CD8$^+$ T cells following SARS-CoV-2 infection at the convalescent stage. Our data further support the view that conventional pulmonary CD103$^+$ T_{RM} cells are less pathogenic in vivo, while the CD69$^+$CD103$^-$ T cells and/or CXCR6^{hi} effector-like tissue-resident cells exhibited more inflammatory and cytopathic features, potentially contributing to the persistent tissue pathology and impaired lung function observed in aged COVID-19 convalescents (Fig. S16).

DISCUSSION

A substantial proportion of COVID-19 patients develop persistent symptoms following resolution of acute illness, that may be accompanied by abnormal chest imaging and impaired lung function testing (*25*). Using automated quantitative analysis, we found that areas of abnormal lung imaging directly correlated with abnormal lung function tests, suggesting that either incomplete tissue recovery even 60 days following the onset of COVID-19 pneumonia and/or persistent tissue remodeling with fibrosis, result in the impaired respiratory function observed in some COVID-19 convalescents. Notably, we did not detect notable SARS-CoV-2 RNA in the BAL of COVID-19 convalescents in our cohorts, which argues against the hypothesis that persistent SRAS-CoV-2 infection in the lung causes chronic pulmonary sequelae.

CD8$^+$ T cells mainly provide protective function during acute respiratory viral infection including SARS-CoV-2 infection (*17*). However, owing to their potent tissue-injurious ability, prolonged and/or dysregulated CD8$^+$ T cell activities can contribute substantially to lung injury and/or pathology development following respiratory viral infection (*26*). Consistent with our prior findings reported in an animal model that CD103$^-$ T_{RM} cells have potent pathogenic potential (*10*), respiratory CD69$^+$CD103$^-$ T cells from aged COVID-19 convalescents were found to express higher levels of cytotoxic or inflammatory molecules and correlated with decreased lung function and worse lung pathology. Whether these human T cells are truly tissue-resident or not in the respiratory tract needs further investigation. CD103$^-$ T cells were enriched with genes associated with the TCR signaling pathway, indicating that they may be sustained by tonic or antigenic signaling within the injured lung. Of note, persistent TCR signaling caused by residual antigen deposition in the lung has been shown to drive the maintenance of CD69$^+$CD103$^-$ T cells following murine influenza virus infection (*10*). It is thus possible that SARS-CoV-2 antigen may also be persistent for a period of time after infectious virus clearance, thereby sustaining and/or stimulating CD103$^-$ T_{RM} cells to cause tissue injury. Alternatively, a portion of CD69$^+$CD103$^-$ T cells may be restricted and stimulated by self-antigen, as those of autoantibodies reported to develop following SARS-CoV-2 infection (*27*–*29*). Notably, circulating CD69$^+$CD103$^-$ T cells, which exhibited lung homing potential, have been implicated in the acute pathogenesis of COVID-19 (*30*). It is thus possible that the same subset of respiratory T cells may play a role in lung pathology during both acute infection and PASC. Additionally, recent reports have found that a portion of non-SARS-CoV-2-exposed individuals have pre-existing SARS-CoV-2 cross-reactive T cells, which likely arose following the exposure of human endemic coronavirus (HCoV) (*31*, *32*). Although we did not detect significant levels of SARS-CoV-2-reactive T cells in aged healthy controls, it remains possible that aged individuals may exhibit increased accumulation of SARS-CoV-2 cross-reactive HCoV-specific T cells due to prior HCoV infections compared to those of young adults. Whether those cross-reactive T cells participate in the immune protection against SARS-CoV-2 re-infection and/or contribute to the development of chronic lung sequelae in COVID-19 convalescents require future investigation.

Besides antigen-dependent killing of CD8$^+$ T cells, a CXCR6^{hi} tissue-resident CD8$^+$ T cell population has been suggested to cause liver damage through ATP-dependent autoaggression during NASH (*24*). The current study similarly identified a CXCR6^{hi} tissue-resident effector-like T cell subset at relatively high levels in the respiratory tract of COVID-19 convalescents. These cells express abundant effector, inflammatory and tissue-destructing gene programs, supporting their potential as contributors to the persistent respiratory tissue pathology and fibrosis following resolution of acute COVID-19 infection. Whether their pathogenic function is independent of antigenic signals in the respiratory tract, as those of the cells in the liver (*24*), warrants future investigation. Nevertheless, we suggest that distinct CD8$^+$ T cell populations with differential pathogenic potential are present in the respiratory tract following SARS-CoV-2 infection. Thus, it would be extremely important to engage the right subset of respiratory CD8$^+$ T cells during potential mucosal vaccination to harness the power of T_{RM} cells in protective immunity but avoiding the unwanted pathology.

Due to the highly invasive nature of the bronchoscopy procedure, the study was limited to a single time point in a selected small cohort of high-risk individuals following acute COVID-19 infection and a matched limited cohort of control

aged healthy individuals. Additionally, the recovery from pulmonary injury often requires an extended period of time in aged individuals. Therefore, the findings reported here may not be generalizable to all COVID-19 patients, such as those young convalescents experiencing PASC. Additional future studies are warranted to determine broader applicability of these observations to younger survivors of mild or severe SARS-CoV-2 infection. Furthermore, due to practical limitations, we were unable to obtain BAL samples from people who recovered from other bacterial or viral pneumonia. Therefore, our study cannot address whether the contribution of exuberant immune responses (particularly dysregulated respiratory $CD8^+$ T cell responses) in pulmonary sequelae is SARS-CoV-2 infection specific or not. Nevertheless, our results have provided the basis for the identification of the etiology of chronic lung diseases induced by acute SARS-CoV-2 infection during aging, which will help in the design of future strategies to avoid immune pathology without compromising the protective local immunity against SARS-CoV-2 in the elderly.

MATERIALS AND METHODS

Study design

The goal of the study was to delineate the immune mechanisms underlying the development of post-acute COVID-19 lung sequelae. We have recruited a cohort of aged (>60 years old) healthy control subjects (n=5) and aged individuals (n=10) who have recovered from acute COVID-19 for two to three months. We performed pulmonary function test and quantitative CT analyses to determine lung physiological function and tissue pathology in the recruited cohort. We then obtained blood samples and BAL fluid from the subjects. Enzyme-linked immunosorbent assay (ELISA) and multiplex cytokine analyses were performed to determine SARS-CoV-2-specific antibody and cytokine levels in the circulation or in the respiratory tract. High-dimensional spectral flow cytometry was performed with PBMCs and BAL cells for the characterization of circulating and respiratory immune profiles in healthy controls and COVID-19 convalescents. We correlated the circulating or respiratory immune profiles with lung functional and pathological parameters to identify the potential involvement of different immune cells in post COVID-19 lung sequelae. Lastly, we performed scRNA-seq to identify potential T cells populations that may contribute to the development of chronic lung sequelae post-acute COVID-19. No outliers were removed.

Ethics statement

This study was approved by Mayo Clinic Institutional Review Boards (protocol ID 20-004911). Informed consent was obtained from all enrolled individuals.

Study cohorts

Survivors of severe COVID-19 pneumonia, defined as patients the required hospitalizations for PCR-proven COVID-19 infection with pneumonia and a need for at least 2L of supplemental oxygen to manage hypoxemic respiratory failure, and a cohort of age-matched normal controls were recruited to the study. The use of a high flow nasal cannula (HFNC) oxygen delivery system was undertaken when hospitalized patients required a level of supplemental oxygen requirement >6L. The HFNC allowed delivery of oxygen at flows of 40-70L enabling approximate Fi02 ranging from 0.45 to a maximum of 0.70 at the highest flow rate. Flow rate was adjusted by the bedside to attain an oxygen saturation of 88-92%.

The COVID-19 convalescent cohort consist of patients between the ages of 60-85 that had no evidence of pre-existing interstitial lung disease or chronic lung disease. For both the COVID-19 and control cohort, prior lung disease was excluded through evaluation of the electronic medical records as well as clinical evaluation prior to performing bronchoscopy. Patients with a history of <10 pack years of smoking and mild COPD with FEV1>80% predicted and FEV1/FVC<0.7 were still eligible for enrollment. At the time of bronchoscopy, control subjects had to have absence of lung infiltrate, fever or any signs or infection. Most of the controls underwent bronchoscopy for evaluation of lung nodule or focal adenopathy of indeterminate cause. The exclusion criteria for both the COVID-19 and CONTROL cohorts included the inability to provide consent to participate in the study; patient under guardianship or curatorship; pre-existing chronic lung disease including interstitial lung disease, pulmonary fibrosis or any other chronic lung disease with the exception of mild COPD as outlined in “inclusion criteria”; active cigarette smoking, vaping or other inhalation use (former smoker providing quit >90 days prior to admission acceptable) and immunocompromised host status due to ongoing therapy with methotrexate, cellcept, azathioprine, prednisone dose >15 mg daily, rituximab, cyclophosphamide or other immunosuppressive or other biologic agents. All COVID-19 patients were enrolled for the study with bronchoscopy and BAL, acquisition of peripheral blood for PBMC and RNA research sample as well as peripheral blood for clinical pre-operative clearance labs, pulmonary function testing, ECG, and imaging of the chest with a non-contrast high resolution CT scan. The COVID-19 patient cohort was enrolled within a 60-90 day window following onset of acute COVID-19 infection, the onset of which was defined as the day when the PCR COVID-19 swab was recorded as positive.

CALIPER analysis of CT image

The analysis of CT image by CALIPER was performed as previously reported (*14*). Initial data processing steps involved extraction of the lung from the surrounding thoracic structures and segmentation into upper, middle, and lower zones. Lung segmentation was performed with an adaptive density-based morphologic approach, whereas airway

segmentation involved iterative 3-dimensional region growing, density thresholding, and connected components analysis. Parenchymal tissue type classification was applied to 15×15×15 voxel volume units using texture analysis, computer vision-based image understanding of volumetric histogram signature mapping features, and 3-dimensional morphology. The CALIPER tool was trained by subspecialty thoracic radiologist consensus assessment of pathologically confirmed data sets (*14*, *34*).

Human pulmonary function testing

Pulmonary function testing was performed on COVID-19 as well as control cohort. All subjects underwent measurement of the forced expiratory vital capacity (FVC) and forced expiratory volume in one second (FEV1) as well as measurement of the Diffusing Capacity of Carbon Monoxide (DLCO). In addition, when possible and tolerated, lung volumes were also determined including total lung capacity (TLC). In all subjects, spirometry was performed in the institutional pulmonary function laboratory at Mayo Clinic in accordance with American Thoracic Society (ATS) guidelines (*35*).

Bronchoscopy and BAL collection

Fiberoptic bronchoscopy and BAL was performed using moderate conscious sedation using standard clinical procedural guidelines in an outpatient bronchoscopy suite. Conscious sedation was administered in accordance with hospital policies and a suitably trained registered nurse provided monitoring throughout the procedure. The scope was wedged into an affected segment pre-determined by review of CT scan. Approximately 100-200 mL of saline was instilled in 20 mL aliquots until 60 mL of lavage fluid was obtained. The specimen was placed on ice and immediately hand carried to lab for analysis. The fluid collected was placed on ice and transferred immediately to the laboratory for processing.

Mouse infection and lung function measurement

Female aged mice were received at 20 to 21 months of age from the National Institutes of Aging and maintained in the same specific pathogen–free conditions for at least 1 month before infection. All mice were used under conditions fully reviewed and approved by the institutional animal care and use committee guidelines at the Mayo Clinic (Rochester, MN). For primary influenza virus infection, influenza A/PR8/34 strain was diluted in fetal bovine serum (FBS)–free Dulbecco's modified Eagle's medium (DMEM) (Corning) on ice and inoculated in anesthetized mice through intranasal route as described before (*11*).

Lung function measurements using forced oscillation technique and the resulting parameters have been previously described (*36*). In brief, animals were anesthetized with an overdose of ketamine/xylazine and tracheostimized with a blunt 18G canula. Animals were connected to the computer-controlled piston (flexiVent) and forced oscillation mechanics were performed under tidal breathing conditions described in (*36*) with a positive-end expiratory pressure of 3 cm H_2O. The measurements were repeated following thorough recruitment of closed airways (2 maneuvers rapidly delivering total lung capacity of air and sustaining the required pressure for several seconds, mimicking holding of a deep breath). Each animal's basal conditions were normalized to their own maximal capacity. Measurement of these parameters before and after lung inflation allows for determination of large and small airway dysfunction under tidal (baseline) breathing conditions. Only measurements that satisfied the constant-phase model fits were used (>90% threshold determined by software). Following this procedure, mice had a heart rate of ~ 60 bpm indicating measurements were done on live subjects. Constant phase model equation was described as before (*37*).

$Z(f) = Raw + i2\pi fI + (Gti - iHti)/(2\pi f)^{\alpha}$

for Raw equal to the Newtonian or airway resistance (R_N) and G & H are frequency independent measures of tissue (ti) resistance and elastance respectively. I is airway inertance and i is the square root of -1, while a is determined by G and H ($a = (2/\pi) \arctan(H_{ti}/G_{ti})$).

Forced oscillatory lung mechanics can be further analyzed to fit a single compartment lung model with constant-phase tissue impedance at each frequency applied to the lung. When this pulmonary input impedence (Z_{in}) data are solved we are left with a real part of Z_{in} indicative of the resistance of airways of increasing size across the broadband signal and a summed imaginary part of Z_{in} called the reactance. Input impedance equation was calculated as reported (*38*) and displayed as mean percentage ± SEM as either raw data (Fig S9) or as a percentage of maximal lung capacity under tidal breathing conditions (Fig. 3D).

Input impedance equation

$Z_{in}(f) = R_N(f) + i2\,\pi\, fI + (G_{ti} - iH_{ti}) / (2\,\pi\, f)^{a}$

Single cell purification

Before the peripheral blood mononuclear cells (PBMC) isolation, 500 μl of blood was saved for innate cell analysis. Plasma was isolated from whole blood by centrifuging at 1,600 rpm, room temperature (RT) for 10 min. After plasma isolation, leftover blood was mixed with PBS then gently put over on Ficoll-Paque (Cytiva) in 50 ml tube. Buffy coat generated by centrifuging at 400xg 40 min RT was collected. For single cell purification from Bronchoalveolar lavage fluid (BAL), BAL was filtered by 70 μm cell strainer (Falcon) then centrifuged 300xg 10 min at 4°C. Supernatant was collected for multiplex assay and ELISA. The cells were collected for flow cytometry analysis and single cell RNA sequencing.

Cytokine and chemokine measurements

BAL and Plasma was isolated as above and aliquots were stored at −80 °C. BAL was further concentrated for 20X using 3 kDa Amicon Ultra-15 Centrifugal Filter Unit (Merck). BAL and plasma were shipped to Eve Technologies (Calgary,

Alberta, Canada) on dry ice, and levels of cytokines and chemokines were measured using the Human Cytokine Array/Chemokine Array 71-403 Plex Panel (HD71).

Flow cytometry analysis

BAL cells or PBMC were stained with antibodies as listed in Data file S3. The high parameter analysis was performed using the Cytek Aurora (Cytek Biosciences). Briefly, cells were washed with FACS buffer (2% of FBS and 0.1% of NaN3 in PBS) and incubated with antibodies of surface markers in the dark for 30 min at 4°C. After wash with PBS, cells were resuspended with Zombie-dye (Biolegend) and incubated at RT for 15 min. For staining transcriptional factors, cells were fixed, permeabilized and stained with Foxp3 transcription factor staining kit (Tonbo) as following manufacturer's manual. For cytokine staining, cells were re-stimulated with predicted overlapping peptide pools against all SARS-CoV-2 proteins. BAL cells or PBMCs were stimulated by peptide pools for 6 hours. The cells were washed with FACS buffer then stained with surface markers. After incubation with viability dye, cells were fixed with fix buffer (Biolegend) at RT for 30 min. The cells were permeabilized with intracellular staining kit (Biolegend) then stained with cytokine antibodies at RT for 1 hour. To detecting Receptor Binding Domain (RBD)-specific B cells, recombinant RBD proteins which were generated from Dr. Taylor's laboratory were incubated with the cells at 4°C for 30 min. RBD-PE and RBD-APC double positive B cells were identified as RBD$^+$ B cells. The same cell number of clean samples which excluded debris, doublets and dead cells were exported then merged as a concatenated file. Uniform Manifold Approximation and Projection (UMAP) was generated with the concatenated file then analyzed for further parameters. Generation of UMAP was performed by FlowJo (BD) plug-in packages.

Antibody production against SARS-CoV-2

General ELISA method has been previously described (*39*). Briefly, Recombinant SARS-CoV-2 proteins including RBD (SinoBiological), Spike S1 (S1) (SinoBiological) or Nucleocapsid protein (N) (GenScript) were pre-coated to 96-well plates overnight at 4°C. The following day, plates were washed with wash buffer (0.05% Tween-20 in PBS) then blocked with 3% of milk in 0.05% PBS-Tween 20 for 1 hour at RT. Plasma or 20X concentrated BAL from control or convalescent patients were diluted in 1% of milk in PBS-Tween 20 starting at a 1:1 or 1:3 dilution respectively then serially diluted by 3. The diluted BAL or plasma were added to the plate and incubated 2 hours at RT. After washing with wash buffer, secondary antibodies diluted in 1% of milk in 0.05% PBS-Tween 20 were added to the plate then incubated for 1.5 hours at RT. Secondary antibodies including anti-human IgG (Sigma), IgM (Sigma) or IgA (Hybridoma Reagent Laboratory) were diluted at 1:5,000, 1:10,000 or 1:1,000 respectively. Plates were intensively washed then developed with TMB buffer (Biolegend) for 10 – 15 min at RT. 2M of sulfuric acid was used as STOP buffer. Plates were read on Microplate reader (Molecular Devices) at 450 nm with SoftMax pro software. The endpoint titers were displayed as a dot for each patient. For correlation matrix analysis, the endpoint titers were cut off at O.D. 0.2.

T cell purification and single-cell RNA-seq / TCR-seq assay

CD3$^+$ T cells were purified from PBMC and BAL cells with the StraightFrom® Whole Blood CD3 MicroBeads (Miltenyi Biotech). The purified T cells were stained with PE-Cy7 anti-human CD3 antibody for the purification efficiency check. The purified PBMC or BAL T cells were labeled with TotalSeq-C0080 anti-human CD8a Antibody, TotalSeq-C0072 anti-human CD4 Antibody, TotalSeq-C0090 Mouse IgG1, κ isotype Ctrl Antibody, and unique TotalSeq-C anti-human Hashtag Antibodies (Biolegend), then were mixed at a ratio of 1:1.

To facilitate the single-cell GEX, Feature Barcode technology for Cell Surface Protein and TCR-seq profiling from the purified T cells, 10X 5′ Library & Gel Bead Kit v1.1 was utilized. 10,000 cells were targeted for single-cell libraries preparation as per the manufacturer's instructions (10x Genomics).

Single cell transcriptome analysis

Single-cell RNA and TCR sequencing data were aligned and quantified using 10X Genomics Cell Ranger Software Suite (v4.0.0) against their corresponding human reference genome (GRCh38) downloaded from 10x Genomics website. Using default settings in Seurat 4.0.1 package, the filtered transcriptome data were then normalized (RNA expression by a factor of 10,000 with log-transformed, Cell Surface Protein of Feature Barcode by a centered log-ratio (CLR)). Then PBMC T cells and BAL T cells were separated based on the unique Hashtag antibodies, the threshold for classification was used as 0.99. The threshold of percent.MT is 10 to exclude dead cells. The RNA expression data was then further scaled based on regressing the number of UMIs detected and the percentage of mitochondrial counts per cell. Principal component analysis (PCA) was performed using the top variable genes. FindNeighbors and FindClusters functions were applied for cell clustering in Seurat for either dataset. The CD8$^+$ T cell and CD4$^+$ T cell cluster were verified by the cell surface protein expression level based on feature barcoding antibodies. Differential gene expression analysis were performed by the function of FindAllMarkers from Seurat with MAST test, GSEA analysis is based on the results of FindAllMarkers with the package of clusterProfiler (*40*). AddModuleScore function was applied for analyzing cell population signatures, the tissue-resident, effector and memory gene sets used were based on published data sets described before (*41*) (*42*, *43*). Pseudotime analysis was performed using SeuratWrappers and Monocle 3 combination, based on the Seurat processed

analysis at the single-cell level. Age unmatched BALT cell were extracted from published dataset GSE151928 (*33*), recovery T cells were set as reference dataset during integrating T cells from healthy donor.

scTCR-seq analysis

Joint analysis of single-cell transcriptomes and TCR repertoires was performed by the function of combineExpression from package of scRepertoire v1.14.0 (*44*). The abundance of cloneTypes were defined based on its frequency (0< Rare <= 1, 1< Small <= 5, 5< Medium <= 20, 20< Large <= 100, 100< Hyperexpanded <= 1,000). Clone size was depicted on UMAP (per cell) by the function of occupiedscRepertoire from package of scRepertoire v1.14.0. Indicated clonotypes were depicted on UMAP (per cell) by the function of highlightClonotypes. Volcano plot of GEG were generated by comparing cells which cloneTypes frequency <= 5 and cloneTypes frequency > 5 by the function of FindAllMarkers from Seurat with MAST test. Gene Expression Omnibus accession number of this study is GSE176201.

SARS-CoV-2 gene detection

Total RNA was extracted from whole BAL cells or PBMCs with Total RNA purification kit (Sigma) according to the manufacturer's instructions. cDNA was synthesized using Random primers (Invitrogen) and MMLV reverse transcriptase (Invitrogen). RT-PCR was performed using Fast SYBR Green PCR Master Mix (Applied Biosystems) with primers nCoV_N1 (Fwd, GACCCCAAAATCAGCGAAAT; Rev, TCTGGTTACTGCCAGTTGAATCTG) and human HPRT (Fwd, GACCAGTCAACAGGGGACAT; Rev, CTGCATTGTTTTGCCAGTGT) in QuantStudio3 (Applied Bioscience). Data were generated by the comparative threshold cycle (△ CT) method by normalizing to HPRT. For positive or negative control of SARS-CoV-2 gene detection, RNAs were extracted non-infected healthy human alveolar macrophages (AMs) or SARS-CoV-2 infected AMs (1 MOI).

Statistical analysis

To compare between two sample groups (CON and CVD), Mann-Whitney test were applied for unpaired comparisons. For analysis between several groups, one or two-way ANOVA was performed. Correlations were assessed by the Pearson correlation coefficient running under R in Rstudio (1.4). All statistical tests were performed using GraphPad Prism version 9 (Graphpad Software) or R (version 4.0.3).

SUPPLEMENTARY MATERIALS

www.science.org/doi/10.1126/sciimmunol.abk1741
Figs. S1 to S16
Data files S1 to S4

REFERENCES AND NOTES

1. M. C. Polidori, H. Sies, L. Ferrucci, T. Benzing, COVID-19 mortality as a fingerprint of biological age. *Ageing Res. Rev.* **67**, 101308 (2021). doi:10.1016/j.arr.2021.101308 Medline
2. X. Yang, Y. Yu, J. Xu, H. Shu, J. Xia, H. Liu, Y. Wu, L. Zhang, Z. Yu, M. Fang, T. Yu, Y. Wang, S. Pan, X. Zou, S. Yuan, Y. Shang, Clinical course and outcomes of critically ill patients with SARS-CoV-2 pneumonia in Wuhan, China: A single-centered, retrospective, observational study. *Lancet Respir. Med.* **8**, 475–481 (2020). doi:10.1016/S2213-2600(20)30079-5 Medline
3. X. Wu, X. Liu, Y. Zhou, H. Yu, R. Li, Q. Zhan, F. Ni, S. Fang, Y. Lu, X. Ding, H. Liu, R. M. Ewing, M. G. Jones, Y. Hu, H. Nie, Y. Wang, 3-month, 6-month, 9-month, and 12-month respiratory outcomes in patients following COVID-19-related hospitalisation: A prospective study. *Lancet Respir. Med.* **9**, 747–754 (2021). doi:10.1016/S2213-2600(21)00174-0 Medline
4. A. Nalbandian, K. Sehgal, A. Gupta, M. V. Madhavan, C. McGroder, J. S. Stevens, J. R. Cook, A. S. Nordvig, D. Shalev, T. S. Sehrawat, N. Ahluwalia, B. Bikdeli, D. Dietz, C. Der-Nigoghossian, N. Liyanage-Don, G. F. Rosner, E. J. Bernstein, S. Mohan, A. A. Beckley, D. S. Seres, T. K. Choueiri, N. Uriel, J. C. Ausiello, D. Accili, D. E. Freedberg, M. Baldwin, A. Schwartz, D. Brodie, C. K. Garcia, M. S. V. Elkind, J. M. Connors, J. P. Bilezikian, D. W. Landry, E. Y. Wan, Post-acute COVID-19 syndrome. *Nat. Med.* **27**, 601–615 (2021). doi:10.1038/s41591-021-01283-z Medline
5. Y. H. Xu, J. H. Dong, W. M. An, X. Y. Lv, X. P. Yin, J. Z. Zhang, L. Dong, X. Ma, H. J. Zhang, B. L. Gao, Clinical and computed tomographic imaging features of novel coronavirus pneumonia caused by SARS-CoV-2. *J. Infect.* **80**, 394–400 (2020). doi:10.1016/j.jinf.2020.02.017 Medline
6. X. Mo, W. Jian, Z. Su, M. Chen, H. Peng, P. Peng, C. Lei, R. Chen, N. Zhong, S. Li, Abnormal pulmonary function in COVID-19 patients at time of hospital discharge. *Eur. Respir. J.* **55**, 2001217 (2020). doi:10.1183/13993003.01217-2020 Medline
7. N. N. Jarjour, D. Masopust, S. C. Jameson, T Cell Memory: Understanding COVID-19. *Immunity* **54**, 14–18 (2021). doi:10.1016/j.immuni.2020.12.009 Medline
8. S. P. Weisberg, B. B. Ural, D. L. Farber, Tissue-specific immunity for a changing world. *Cell* **184**, 1517–1529 (2021). doi:10.1016/j.cell.2021.01.042 Medline
9. S. C. Sasson, C. L. Gordon, S. N. Christo, P. Klenerman, L. K. Mackay, Local heroes or villains: Tissue-resident memory T cells in human health and disease. *Cell. Mol. Immunol.* **17**, 113–122 (2020). doi:10.1038/s41423-019-0359-1 Medline
10. Z. Wang, S. Wang, N. P. Goplen, C. Li, I. S. Cheon, Q. Dai, S. Huang, J. Shan, C. Ma, Z. Ye, M. Xiang, A. H. Limper, E. C. Porquera, J. E. Kohlmeier, M. H. Kaplan, N. Zhang, A. J. Johnson, R. Vassallo, J. Sun, PD-1^{hi} CD8^{+} resident memory T cells balance immunity and fibrotic sequelae. *Sci. Immunol.* **4**, eaaw1217 (2019). doi:10.1126/sciimmunol.aaw1217 Medline
11. N. P. Goplen, Y. Wu, Y. M. Son, C. Li, Z. Wang, I. S. Cheon, L. Jiang, B. Zhu, K. Ayasoufi, E. N. Chini, A. J. Johnson, R. Vassallo, A. H. Limper, N. Zhang, J. Sun, Tissue-resident CD8^{+} T cells drive age-associated chronic lung sequelae after viral pneumonia. *Sci. Immunol.* **5**, eabc4557 (2020). doi:10.1126/sciimmunol.abc4557 Medline
12. J. M. Dan, J. Mateus, Y. Kato, K. M. Hastie, E. D. Yu, C. E. Faliti, A. Grifoni, S. I. Ramirez, S. Haupt, A. Frazier, C. Nakao, V. Rayaprolu, S. A. Rawlings, B. Peters, F. Krammer, V. Simon, E. O. Saphire, D. M. Smith, D. Weiskopf, A. Sette, S. Crotty, Immunological memory to SARS-CoV-2 assessed for up to 8 months after infection. *Science* **371**, ••• (2021). doi:10.1126/science.abf4063 Medline
13. L. B. Rodda, J. Netland, L. Shehata, K. B. Pruner, P. A. Morawski, C. D. Thouvenel, K. K. Takehara, J. Eggenberger, E. A. Hemann, H. R. Waterman, M. L. Fahning, Y. Chen, M. Hale, J. Rathe, C. Stokes, S. Wrenn, B. Fiala, L. Carter, J. A. Hamerman, N. P. King, M. Gale Jr., D. J. Campbell, D. J. Rawlings, M. Pepper, Functional SARS-CoV-2-Specific Immune Memory Persists after Mild COVID-19. *Cell* **184**, 169–183.e17 (2021). doi:10.1016/j.cell.2020.11.029 Medline
14. J. Jacob, B. J. Bartholmai, S. Rajagopalan, M. Kokosi, A. Nair, R. Karwoski, S. M. Raghunath, S. L. Walsh, A. U. Wells, D. M. Hansell, Automated Quantitative Computed Tomography Versus Visual Computed Tomography Scoring in Idiopathic Pulmonary Fibrosis: Validation Against Pulmonary Function. *J. Thorac. Imaging* **31**, 304–311 (2016). doi:10.1097/RTI.0000000000000220 Medline
15. M. Liao, Y. Liu, J. Yuan, Y. Wen, G. Xu, J. Zhao, L. Cheng, J. Li, X. Wang, F. Wang, L. Liu, I. Amit, S. Zhang, Z. Zhang, Single-cell landscape of bronchoalveolar immune cells in patients with COVID-19. *Nat. Med.* **26**, 842–844 (2020). doi:10.1038/s41591-020-0901-9 Medline
16. P. A. Szabo, P. Dogra, J. I. Gray, S. B. Wells, T. J. Connors, S. P. Weisberg, I. Krupska, R. Matsumoto, M. M. L. Poon, E. Idzikowski, S. E. Morris, C. Pasin, A. J. Yates, A. Ku, M. Chait, J. Davis-Porada, X. V. Guo, J. Zhou, M. Steinle, S. Mackay, A. Saqi, M. R. Baldwin, P. A. Sims, D. L. Farber, Longitudinal profiling of respiratory and systemic immune responses reveals myeloid cell-driven lung inflammation in

severe COVID-19. *Immunity* **54**, 797–814.e6 (2021). doi:10.1016/j.immuni.2021.03.005 Medline
17. A. Sette, S. Crotty, Adaptive immunity to SARS-CoV-2 and COVID-19. *Cell* **184**, 861–880 (2021). doi:10.1016/j.cell.2021.01.007 Medline
18. Y. M. Son, I. S. Cheon, Y. Wu, C. Li, Z. Wang, X. Gao, Y. Chen, Y. Takahashi, Y. X. Fu, A. L. Dent, M. H. Kaplan, J. J. Taylor, W. Cui, J. Sun, Tissue-resident CD4+ T helper cells assist the development of protective respiratory B and CD8+ T cell memory responses. *Sci. Immunol.* **6**, eabb6852 (2021). doi:10.1126/sciimmunol.abb6852 Medline
19. N. Swarnalekha, D. Schreiner, L. C. Litzler, S. Iftikhar, D. Kirchmeier, M. Künzli, Y. M. Son, J. Sun, E. A. Moreira, C. G. King, T resident helper cells promote humoral responses in the lung. *Sci. Immunol.* **6**, eabb6808 (2021). doi:10.1126/sciimmunol.abb6808 Medline
20. A. Grifoni, D. Weiskopf, S. I. Ramirez, J. Mateus, J. M. Dan, C. R. Moderbacher, S. A. Rawlings, A. Sutherland, L. Premkumar, R. S. Jadi, D. Marrama, A. M. de Silva, A. Frazier, A. F. Carlin, J. A. Greenbaum, B. Peters, F. Krammer, D. M. Smith, S. Crotty, A. Sette, Targets of T Cell Responses to SARS-CoV-2 Coronavirus in Humans with COVID-19 Disease and Unexposed Individuals. *Cell* **181**, 1489–1501.e15 (2020). doi:10.1016/j.cell.2020.05.015 Medline
21. Q. Guo, Y. Zhao, J. Li, J. Liu, X. Yang, X. Guo, M. Kuang, H. Xia, Z. Zhang, L. Cao, Y. Luo, L. Bao, X. Wang, X. Wei, W. Deng, N. Wang, L. Chen, J. Chen, H. Zhu, R. Gao, C. Qin, X. Wang, F. You, Induction of alarmin S100A8/A9 mediates activation of aberrant neutrophils in the pathogenesis of COVID-19. *Cell Host Microbe* **29**, 222–235.e4 (2021). doi:10.1016/j.chom.2020.12.016 Medline
22. S. S. Ng, F. De Labastida Rivera, J. Yan, D. Corvino, I. Das, P. Zhang, R. Kuns, S. B. Chauhan, J. Hou, X. Y. Li, T. C. M. Frame, B. A. McEnroe, E. Moore, J. Na, J. A. Engel, M. S. F. Soon, B. Singh, A. J. Kueh, M. J. Herold, M. Montes de Oca, S. S. Singh, P. T. Bunn, A. R. Aguilera, M. Casey, M. Braun, N. Ghazanfari, S. Wani, Y. Wang, F. H. Amante, C. L. Edwards, A. Haque, W. C. Dougall, O. P. Singh, A. G. Baxter, M. W. L. Teng, A. Loukas, N. L. Daly, N. Cloonan, M. A. Degli-Esposti, J. Uzonna, W. R. Heath, T. Bald, S. K. Tey, K. Nakamura, G. R. Hill, R. Kumar, S. Sundar, M. J. Smyth, C. R. Engwerda, The NK cell granule protein NKG7 regulates cytotoxic granule exocytosis and inflammation. *Nat. Immunol.* **21**, 1205–1218 (2020). doi:10.1038/s41590-020-0758-6 Medline
23. D. A. Mogilenko, O. Shpynov, P. S. Andhey, L. Arthur, A. Swain, E. Esaulova, S. Brioschi, I. Shchukina, M. Kerndl, M. Bambouskova, Z. Yao, A. Laha, K. Zaitsev, S. Burdess, S. Gillfilan, S. A. Stewart, M. Colonna, M. N. Artyomov, Comprehensive Profiling of an Aging Immune System Reveals Clonal GZMK+ CD8+ T Cells as Conserved Hallmark of Inflammaging. *Immunity* **54**, 99–115.e12 (2021). doi:10.1016/j.immuni.2020.11.005 Medline
24. M. Dudek, D. Pfister, S. Donakonda, P. Filpe, A. Schneider, M. Laschinger, D. Hartmann, N. Hüser, P. Meiser, F. Bayerl, D. Inverso, J. Wigger, M. Sebode, R. Öllinger, R. Rad, S. Hegenbarth, M. Anton, A. Guillot, A. Bowman, D. Heide, F. Müller, P. Ramadori, V. Leone, C. Garcia-Caceres, T. Gruber, G. Seifert, A. M. Kabat, J. P. Mallm, S. Reider, M. Effenberger, S. Roth, A. T. Billeter, B. Müller-Stich, E. J. Pearce, F. Koch-Nolte, R. Käser, H. Tilg, R. Thimme, T. Boettler, F. Tacke, J. F. Dufour, D. Haller, P. J. Murray, R. Heeren, D. Zehn, J. P. Böttcher, M. Heikenwälder, P. A. Knolle, Auto-aggressive CXCR6+ CD8 T cells cause liver immune pathology in NASH. *Nature* **592**, 444–449 (2021). doi:10.1038/s41586-021-03233-8 Medline
25. C. Huang, L. Huang, Y. Wang, X. Li, L. Ren, X. Gu, L. Kang, L. Guo, M. Liu, X. Zhou, J. Luo, Z. Huang, S. Tu, Y. Zhao, L. Chen, D. Xu, Y. Li, C. Li, L. Peng, Y. Li, W. Xie, D. Cui, L. Shang, G. Fan, J. Xu, G. Wang, Y. Wang, J. Zhong, C. Wang, J. Wang, D. Zhang, B. Cao, 6-month consequences of COVID-19 in patients discharged from hospital: A cohort study. *Lancet* **397**, 220–232 (2021). doi:10.1016/S0140-6736(20)32656-8 Medline
26. N. P. Goplen, I. S. Cheon, J. Sun, Age-Related Dynamics of Lung-Resident Memory CD8+ T Cells in the Age of COVID-19. *Front. Immunol.* **12**, 636118 (2021). doi:10.3389/fimmu.2021.636118 Medline
27. P. Bastard, L. B. Rosen, Q. Zhang, E. Michailidis, H. H. Hoffmann, Y. Zhang, K. Dorgham, Q. Philippot, J. Rosain, V. Béziat, J. Manry, E. Shaw, L. Haljasmägi, P. Peterson, L. Lorenzo, L. Bizien, S. Trouillet-Assant, K. Dobbs, A. A. de Jesus, A. Belot, A. Kallaste, E. Catherinot, Y. Tandjaoui-Lambiotte, J. Le Pen, G. Kerner, B. Bigio, Y. Seeleuthner, R. Yang, A. Bolze, A. N. Spaan, O. M. Delmonte, M. S. Abers, A. Aiuti, G. Casari, V. Lampasona, L. Piemonti, F. Ciceri, K. Bilguvar, R. P. Lifton, M. Vasse, D. M. Smadja, M. Migaud, J. Hadjadj, B. Terrier, D. Duffy, L. Quintana-Murci, D. van de Beek, L. Roussel, D. C. Vinh, S. G. Tangye, F. Haerynck, D. Dalmau, J. Martinez-Picado, P. Brodin, M. C. Nussenzweig, S. Boisson-Dupuis, C. Rodríguez-Gallego, G. Vogt, T. H. Mogensen, A. J. Oler, J. Gu, P. D. Burbelo, J. I. Cohen, A. Biondi, L. R. Bettini, M. D'Angio, P. Bonfanti, P. Rossignol, J. Mayaux, F. Rieux-Laucat, E. S. Husebye, F. Fusco, M. V. Ursini, L. Imberti, A. Sottini, S. Paghera, E. Quiros-Roldan, C. Rossi, R. Castagnoli, D. Montagna, A. Licari, G. L. Marseglia, X. Duval, J. Ghosn, J. S. Tsang, R. Goldbach-Mansky, K. Kisand, M. S. Lionakis, A. Puel, S. Y. Zhang, S. M. Holland, G. Gorochov, E. Jouanguy, C. M. Rice, A. Cobat, L. D. Notarangelo, L. Abel, H. C. Su, J. L. Casanova; HGID Lab; NIAID-USUHS Immune Response to COVID Group; COVID Clinicians; COVID-STORM Clinicians; Imagine COVID Group; French COVID Cohort Study Group; Milieu Intérieur Consortium; CoV-Contact Cohort; Amsterdam UMC Covid-19 Biobank; COVID Human Genetic Effort, Autoantibodies against type I IFNs in patients with life-threatening COVID-19. *Science* **370**, eabd4585 (2020). doi:10.1126/science.abd4585 Medline
28. A. J. Combes, T. Courau, N. F. Kuhn, K. H. Hu, A. Ray, W. S. Chen, N. W. Chew, S. J. Cleary, D. Kushnoor, G. C. Reeder, A. Shen, J. Tsui, K. J. Hiam-Galvez, P. Muñoz-Sandoval, W. S. Zhu, D. S. Lee, Y. Sun, R. You, M. Magnen, L. Rodriguez, K. W. Im, N. K. Serwas, A. Leligdowicz, C. R. Zamecnik, R. P. Loudermilk, M. R. Wilson, C. J. Ye, G. K. Fragiadakis, M. R. Looney, V. Chan, A. Ward, S. Carrillo, M. Matthay, D. J. Erle, P. G. Woodruff, C. Langelier, K. Kangelaris, C. M. Hendrickson, C. Calfee, A. A. Rao, M. F. Krummel; UCSF COMET Consortium, Global absence and targeting of protective immune states in severe COVID-19. *Nature* **591**, 124–130 (2021). doi:10.1038/s41586-021-03234-7 Medline
29. E. Y. Wang, T. Mao, J. Klein, Y. Dai, J. D. Huck, F. Liu, N. S. Zheng, T. Zhou, B. Israelow, P. Wong, C. Lucas, J. Silva, J. E. Oh, E. Song, E. S. Perotti, S. Fischer, M. Campbell, J. B. Fournier, A. L. Wyllie, C. B. F. Vogels, I. M. Ott, C. C. Kalinich, M. E. Petrone, A. E. Watkins, C. D. Cruz, S. F. Farhadian, W. L. Schulz, N. D. Grubaugh, A. I. Ko, A. Iwasaki, A. M. Ring, Diverse Functional Autoantibodies in Patients with COVID-19. *medRxiv*, (2020).
30. J. Neidleman, X. Luo, A. F. George, M. McGregor, J. Yang, C. Yun, V. Murray, G. Gill, W. C. Greene, J. Vasquez, S. A. Lee, E. Ghosn, K. L. Lynch, N. R. Roan, Distinctive features of SARS-CoV-2-specific T cells predict recovery from severe COVID-19. *Cell Rep.* **36**, 109414 (2021). doi:10.1016/j.celrep.2021.109414 Medline
31. M. Lipsitch, Y. H. Grad, A. Sette, S. Crotty, Cross-reactive memory T cells and herd immunity to SARS-CoV-2. *Nat. Rev. Immunol.* **20**, 709–713 (2020). doi:10.1038/s41577-020-00460-4 Medline
32. V. Mallajosyula, C. Ganjavi, S. Chakraborty, A. M. McSween, A. J. Pavlovitch-Bedzyk, J. Wilhelmy, A. Nau, M. Manohar, K. C. Nadeau, M. M. Davis, CD8+ T cells specific for conserved coronavirus epitopes correlate with milder disease in COVID-19 patients. *Sci. Immunol.* **6**, eabg5669 (2021). doi:10.1126/sciimmunol.abg5669 Medline
33. K. J. Mould, C. M. Moore, S. A. McManus, A. L. McCubbrey, J. D. McClendon, C. L. Griesmer, P. M. Henson, W. J. Janssen, Airspace Macrophages and Monocytes Exist in Transcriptionally Distinct Subsets in Healthy Adults. *Am. J. Respir. Crit. Care Med.* **203**, 946–956 (2021). doi:10.1164/rccm.202005-1989OC Medline
34. F. Maldonado, T. Moua, S. Rajagopalan, R. A. Karwoski, S. Raghunath, P. A. Decker, T. E. Hartman, B. J. Bartholmai, R. A. Robb, J. H. Ryu, Automated quantification of radiological patterns predicts survival in idiopathic pulmonary fibrosis. *Eur. Respir. J.* **43**, 204–212 (2014). doi:10.1183/09031936.00071812 Medline
35. B. L. Graham, I. Steenbruggen, M. R. Miller, I. Z. Barjaktarevic, B. G. Cooper, G. L. Hall, T. S. Hallstrand, D. A. Kaminsky, K. McCarthy, M. C. McCormack, C. E. Oropez, M. Rosenfeld, S. Stanojevic, M. P. Swanney, B. R. Thompson, Standardization of Spirometry 2019 Update. An Official American Thoracic Society and European Respiratory Society Technical Statement. *Am. J. Respir. Crit. Care Med.* **200**, e70–e88 (2019). doi:10.1164/rccm.201908-1590ST Medline
36. N. Goplen, M. Z. Karim, Q. Liang, M. M. Gorska, S. Rozario, L. Guo, R. Alam, Combined sensitization of mice to extracts of dust mite, ragweed, and Aspergillus species breaks through tolerance and establishes chronic features of asthma. *J. Allergy Clin. Immunol.* **123**, 925–32.e11 (2009). doi:10.1016/j.jaci.2009.02.009 Medline
37. J. H. Bates, K. R. Lutchen, The interface between measurement and modeling of peripheral lung mechanics. *Respir. Physiol. Neurobiol.* **148**, 153–164 (2005). doi:10.1016/j.resp.2005.04.021 Medline
38. Z. Hantos, F. Peták, A. Adamicza, T. Asztalos, J. Tolnai, J. J. Fredberg, Mechanical

impedance of the lung periphery. *J. Appl. Physiol.* **83**, 1595–1601 (1997). doi:10.1152/jappl.1997.83.5.1595 Medline
39. C. Rydyznski Moderbacher, S. I. Ramirez, J. M. Dan, A. Grifoni, K. M. Hastie, D. Weiskopf, S. Belanger, R. K. Abbott, C. Kim, J. Choi, Y. Kato, E. G. Crotty, C. Kim, S. A. Rawlings, J. Mateus, L. P. V. Tse, A. Frazier, R. Baric, B. Peters, J. Greenbaum, E. Ollmann Saphire, D. M. Smith, A. Sette, S. Crotty, Antigen-Specific Adaptive Immunity to SARS-CoV-2 in Acute COVID-19 and Associations with Age and Disease Severity. *Cell* **183**, 996–1012.e19 (2020). doi:10.1016/j.cell.2020.09.038 Medline
40. G. Yu, L. G. Wang, Y. Han, Q. Y. He, clusterProfiler: An R package for comparing biological themes among gene clusters. *OMICS* **16**, 284–287 (2012). doi:10.1089/omi.2011.0118 Medline
41. J. J. Milner, C. Toma, B. Yu, K. Zhang, K. Omilusik, A. T. Phan, D. Wang, A. J. Getzler, T. Nguyen, S. Crotty, W. Wang, M. E. Pipkin, A. W. Goldrath, Runx3 programs CD8^{+} T cell residency in non-lymphoid tissues and tumours. *Nature* **552**, 253–257 (2017). doi:10.1038/nature24993 Medline
42. J. J. Milner, C. Toma, Z. He, N. S. Kurd, Q. P. Nguyen, B. McDonald, L. Quezada, C. E. Widjaja, D. A. Witherden, J. T. Crowl, L. A. Shaw, G. W. Yeo, J. T. Chang, K. D. Omilusik, A. W. Goldrath, Heterogenous Populations of Tissue-Resident CD8^{+} T Cells Are Generated in Response to Infection and Malignancy. *Immunity* **52**, 808–824.e7 (2020). doi:10.1016/j.immuni.2020.04.007 Medline
43. M. Dudek, D. Pfister, S. Donakonda, P. Filpe, A. Schneider, M. Laschinger, D. Hartmann, N. Hüser, P. Meiser, F. Bayerl, D. Inverso, J. Wigger, M. Sebode, R. Öllinger, R. Rad, S. Hegenbarth, M. Anton, A. Guillot, A. Bowman, D. Heide, F. Müller, P. Ramadori, V. Leone, C. Garcia-Caceres, T. Gruber, G. Seifert, A. M. Kabat, J. P. Mallm, S. Reider, M. Effenberger, S. Roth, A. T. Billeter, B. Müller-Stich, E. J. Pearce, F. Koch-Nolte, R. Käser, H. Tilg, R. Thimme, T. Boettler, F. Tacke, J. F. Dufour, D. Haller, P. J. Murray, R. Heeren, D. Zehn, J. P. Böttcher, M. Heikenwälder, P. A. Knolle, Auto-aggressive CXCR6^{+} CD8 T cells cause liver immune pathology in NASH. *Nature* **592**, 444–449 (2021). doi:10.1038/s41586-021-03233-8 Medline
44. H. Koch, D. Starenki, S. J. Cooper, R. M. Myers, Q. Li, powerTCR: A model-based approach to comparative analysis of the clone size distribution of the T cell receptor repertoire. *PLOS Comput. Biol.* **14**, e1006571 (2018). doi:10.1371/journal.pcbi.1006571 Medline

Acknowledgments: We thank Mayo genomic core for technical assistance. We thank T. Braciale for critical reading the manuscript. Cartoon in Fig. S16 was created with BioRender.com. **Funding:** This study was funded by the US National Institutes of Health grants AI147394, AG047156 and AG069264 and Mayo Clinic CBD Research Fund to J.S.; AI147394S1 to J.S. and R.V.; 1U01AI131566-0 to R.C.; R01 AI057459 to M.K.; R01 NS103212 and RF1 NS122174 to A.J. and NIH contract 75N9301900065 to A.S. and D.W. **Author contributions**: Conception and design, I.C., C.L., Y.S., R.V. & J.S.; Acquisition of data, I.C., C.L., Y.S., N.G., Y.W., T.C., Z.W., T.J., Y.L., S.H., T.C, L.H.,B.B., R.K., R.V.; Analysis and interpretation of data, I.C., C.L., Y.S., R.V. & J.S. ; Writing of the manuscript, I.C., C.L., Y.S. & J.S.; Critical reagents and manuscript editing, E.G., K.A., D.W., J.B., R.C., A.J., E.E., H.D., H.L., J.T., M.K., A.S., B.B., R.K. Funding acquisition, R.V. & J.S. Competing Interests: J.S. receives research grants from Humanigen. R.V. receives research grants from Hurvis Foundation, Pfizer, Bristol Myers Squibb and Sun Pharma. B.B. has served as a scientific advisor to AstraZeneca and Promedior and receives royalties from license of CALIPER software to Imbio, LLC. A.S. is currently a consultant for Gritstone, Flow Pharma, Arcturus, Epitogenesis, Oxfordimmunotech, Caprion and Avalia. LJI has filed for patent protection for various aspects of T cell epitope and vaccine design work. **Data and materials availability:** scRNA-seq data are available from the Gene Expression Omnibus under accession number GSE176201. All other data needed to evaluate the conclusions of the paper are present in the paper or the Supplementary Materials. This work is licensed under a Creative Commons Attribution 4.0 International (CC BY 4.0) license, which permits unrestricted use, distribution, and reproduction in any medium, provided the original work is properly cited. To view a copy of this license, visit https://creativecommons.org/licenses/by/4.0/. This license does not apply to figures/photos/artwork or other content included in the article that is credited to a third party; obtain authorization from the rights holder before using such material.

Submitted 25 June 2021
Accepted 24 September 2021
Published First Release 30 September 2021
10.1126/sciimmunol.abk1741

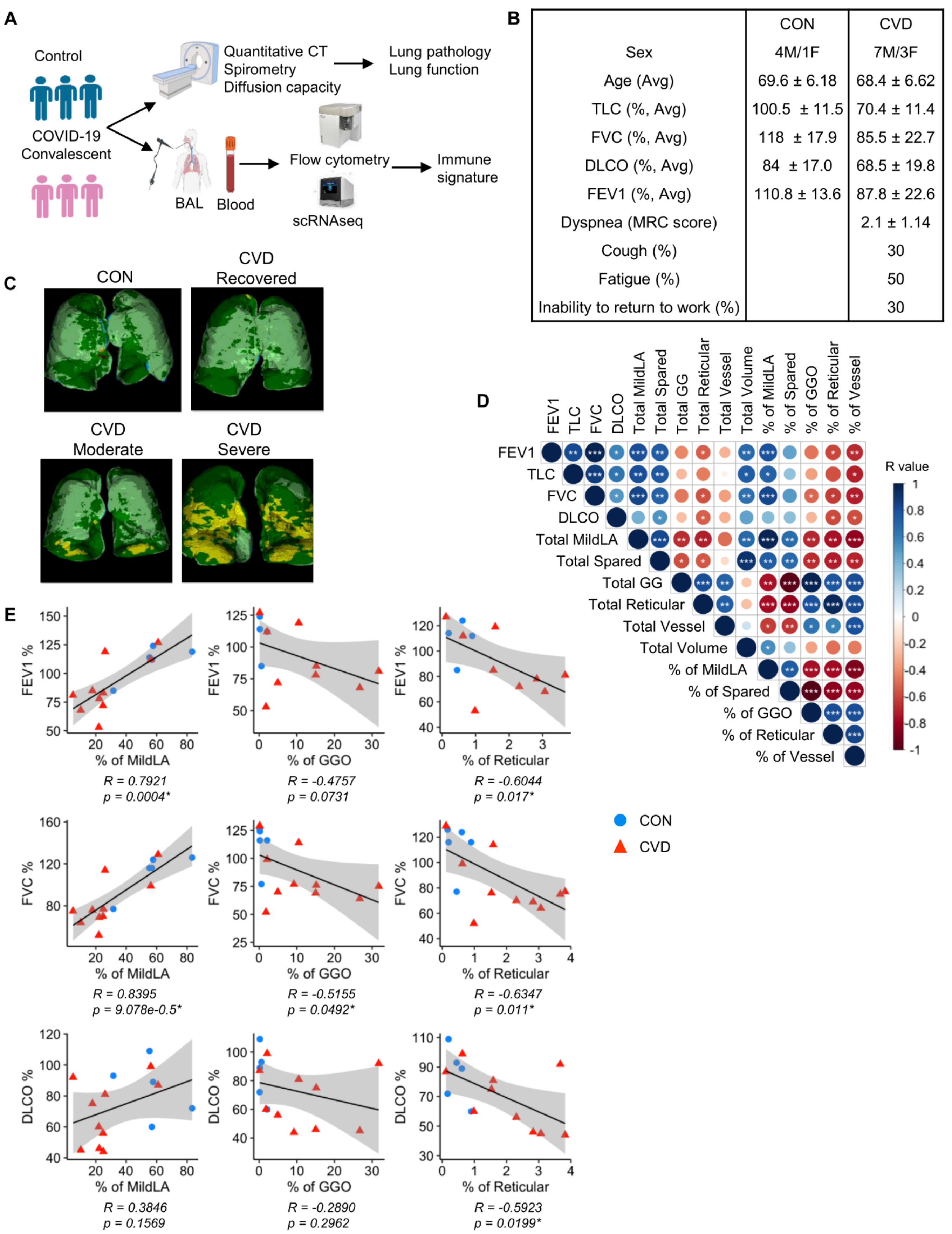

	CON	CVD
Sex	4M/1F	7M/3F
Age (Avg)	69.6 ± 6.18	68.4 ± 6.62
TLC (%, Avg)	100.5 ± 11.5	70.4 ± 11.4
FVC (%, Avg)	118 ± 17.9	85.5 ± 22.7
DLCO (%, Avg)	84 ± 17.0	68.5 ± 19.8
FEV1 (%, Avg)	110.8 ± 13.6	87.8 ± 22.6
Dyspnea (MRC score)		2.1 ± 1.14
Cough (%)		30
Fatigue (%)		50
Inability to return to work (%)		30

Fig. 1. Functional and pathological characterization of lung sequelae in aged COVID-19 convalescents. A. Schematics of experimental procedure. **B.** Summary of control and COVID-19 convalescent age, sex, lung function and condition data. **C.** Lung regions of pathology revealed by quantitative CT analysis by CALIPER (Green/Dark Green, "spared/relatively spared"; Yellow, "GGO"; Orange, "Reticular/Consolidation"). **D.** Correlation of lung functional parameters with lung pathological features. FEV1 (forced expiratory volume in 1 s), TLC (total lung capacity), FVC (forced vital capacity) and DLCO (diffusion capacity for carbon monoxide). Significant correlations were indicated by white asterisks. **E.** Correlation of FEV1, FVC and DLCO with % of mild lung area, % of GGO region and % of Reticular regions in the lung. Significant correlations were indicated by asterisks. * $p < 0.05$.

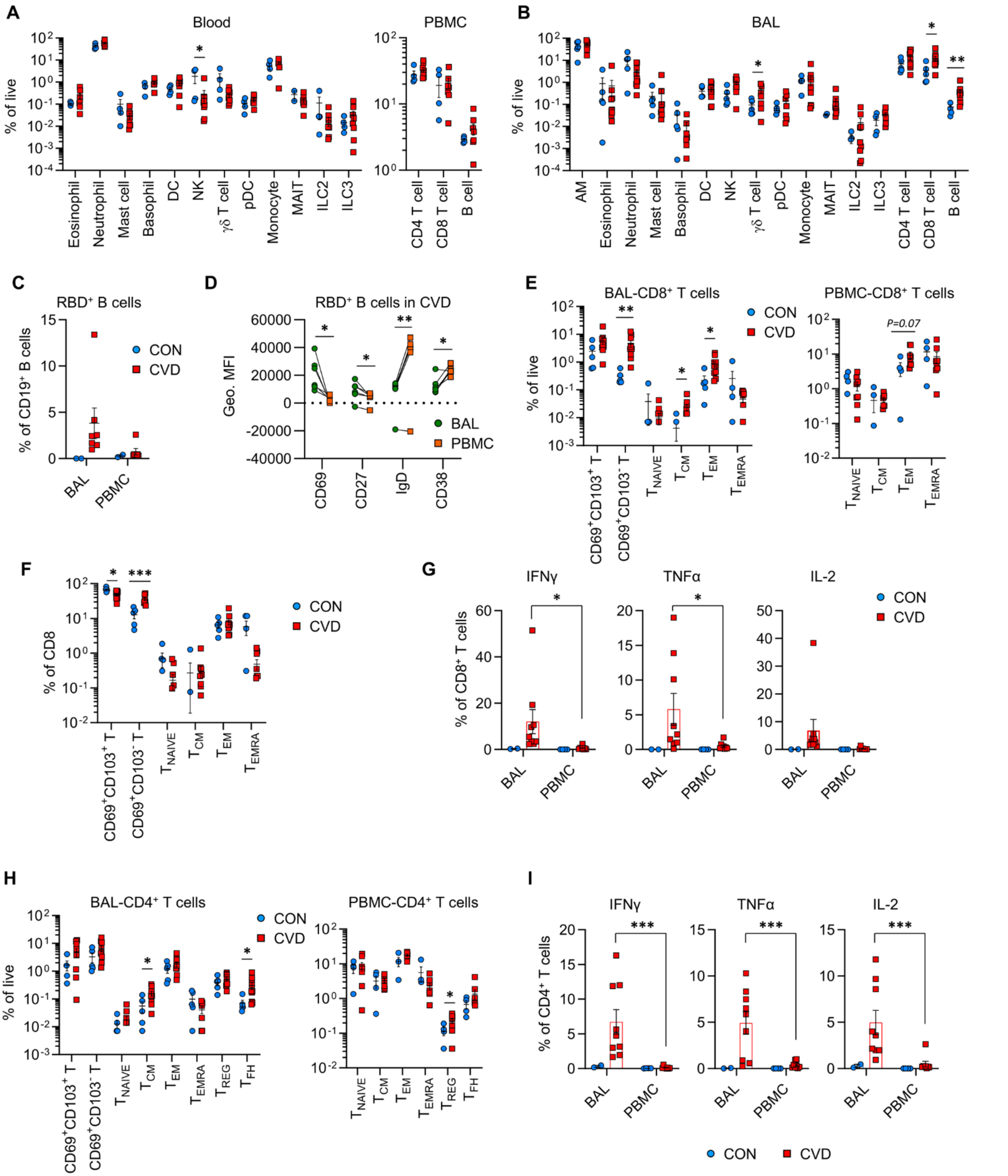

A
Blood
PBMC
% of live
Eosinophil
Neutrophil
Mast cell
Basophil
DC
NK
γδ T cell
pDC
Monocyte
MAIT
ILC2
ILC3
CD4 T cell
CD8 T cell
B cell
B
BAL
AM
C
RBD⁺ B cells
% of CD19⁺ B cells
CON
CVD
BAL
PBMC
D
RBD⁺ B cells in CVD
Geo. MFI
CD69
CD27
IgD
CD38
E
BAL-CD8⁺ T cells
PBMC-CD8⁺ T cells
P=0.07
CD69⁺CD103⁺ T
CD69⁺CD103⁻ T
T NAIVE
T CM
T EM
T EMRA
F
% of CD8
G
IFNγ
TNFα
IL-2
% of CD8⁺ T cells
H
BAL-CD4⁺ T cells
PBMC-CD4⁺ T cells
T REG
T FH
I
% of CD4⁺ T cells

Fig. 2. Characterization of respiratory and circulating immune memory in COVID-19 convalescents. A. Percentage of indicated innate immune cell types in the whole blood (left panel) or adaptive immune cells in PBMCs (right panel). **B.** Percentage of indicated immune cell types in the BAL. **C**. Percentage of RBD-specific B cells within B cell compartment of BAL or PBMC. **D**. CD69, CD27, IgD and CD38 expression levels of RBD-specific B cells in BAL or PBMC. **E**. Percentage of indicated $CD8^+$ T cell subsets based on live cells in the BAL or PBMC. **F**. Percentage of indicated $CD8^+$ T cell subsets in total $CD8^+$ T cells. **G**. Percentage of IFN-γ, TNF or $IL\text{-}2^+$ $CD8^+$ T cells in the BAL or PBMC following stimulation with SARS-CoV-2 peptide pools. **H**. Percentage of indicated $CD4^+$ T cell subsets based on live cells in the BAL or PBMC. **I.** Percentage of IFN-γ, TNF or $IL\text{-}2^+$ $CD4^+$ T cells in the BAL or PBMC following stimulation with SARS-CoV-2 peptide pools. **A**, **B, E, F** and **H**. Statistical significance was calculated using Mann-Whitney test. * $p < 0.05$. **C**. Statistical difference was performed using two-way ANOVA. **D**. Statistical significance was calculated using paired *t* test. **G** and **I**. Statistical significance was calculated using two-way ANOVA following Fisher's LSD test. * $p < 0.05$.

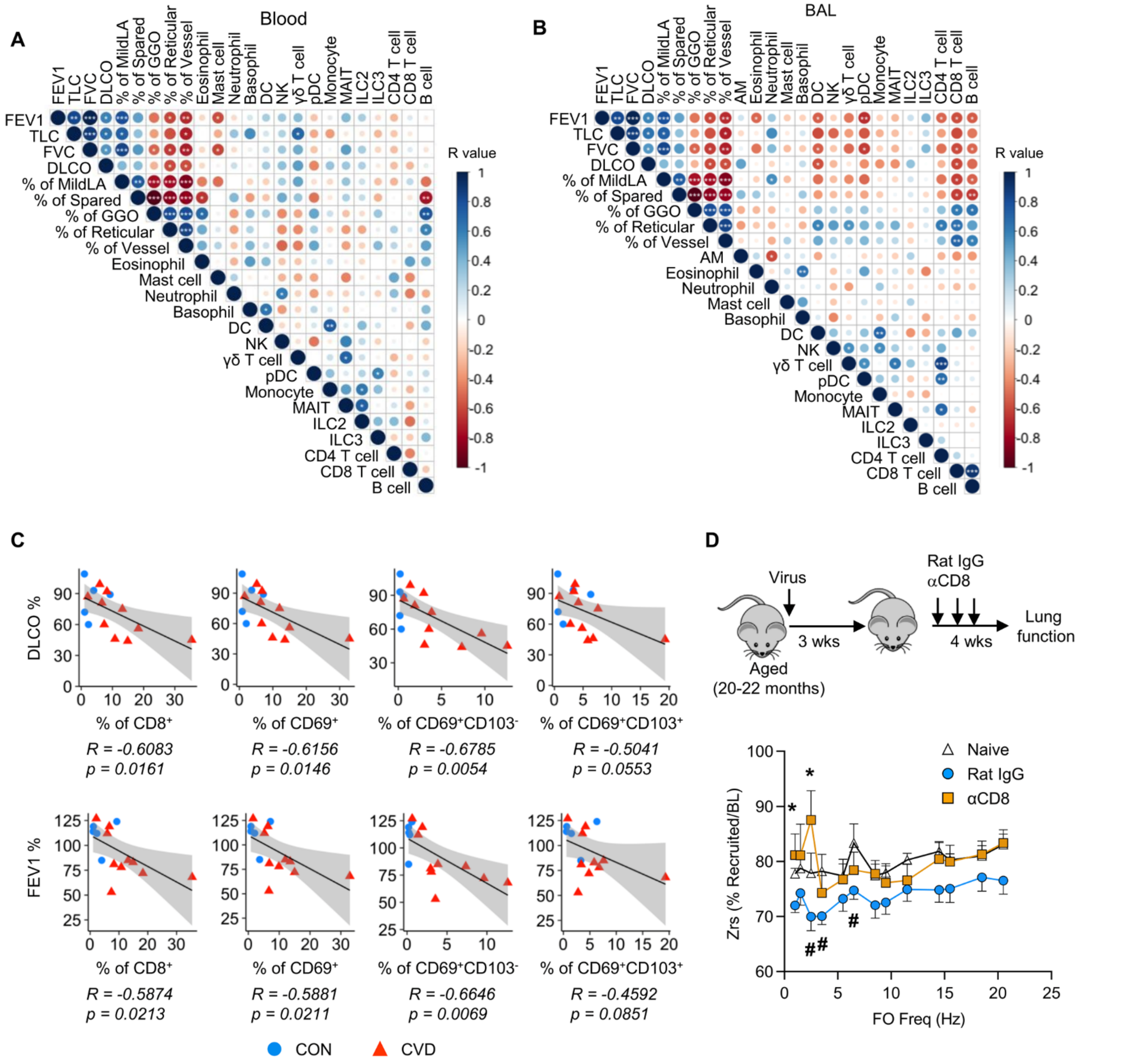

Fig. 3. Dysregulated CD8⁺ T cell responses as a potential driver of chronic lung sequelae. **A.** Correlation of blood immune cell types with lung functional parameters and quantitative lung pathology parameters. **B.** Correlation of BAL immune cell types with lung functional parameters and quantitative lung pathology parameters. **C.** Correlation of DLCO and FEV1 with % of total $CD8^+$, $CD69^+$ $CD8^+$, $CD69^+CD103^-$ $CD8^+$ or $CD69^+CD103^+$ $CD8^+$ T cells in the total BAL cells of healthy control and COVID-19 convalescents. **D.** Aged mice were infected or not (Naïve) with influenza virus. Mice receiving $CD8^+$ T cell depleting Ab (αCD8) or control Ab (Rat IgG) starting at 21 days post infection. Lung function was measured at 50 days post infection. Input impedance (Zrs) was measured during tidal breathing and following recruitment of closed airways and displayed as the percent utilization at baseline for each frequency in the repeated forced oscillation (FO) waveform. **A, B.** r values are indicated by color and circle size. Significant correlations were indicated by white asterisks. **D.** Lung function data were cumulative from 3 independent experiments and raw data can be found in Fig. S9. *$p < 0.05$ for Rat IgG (n=16) versus CD8-depleted group (n=8) or Naïve (n=7) versus Rat IgG group (#) for two-way ANOVA following Fisher's LSD test.

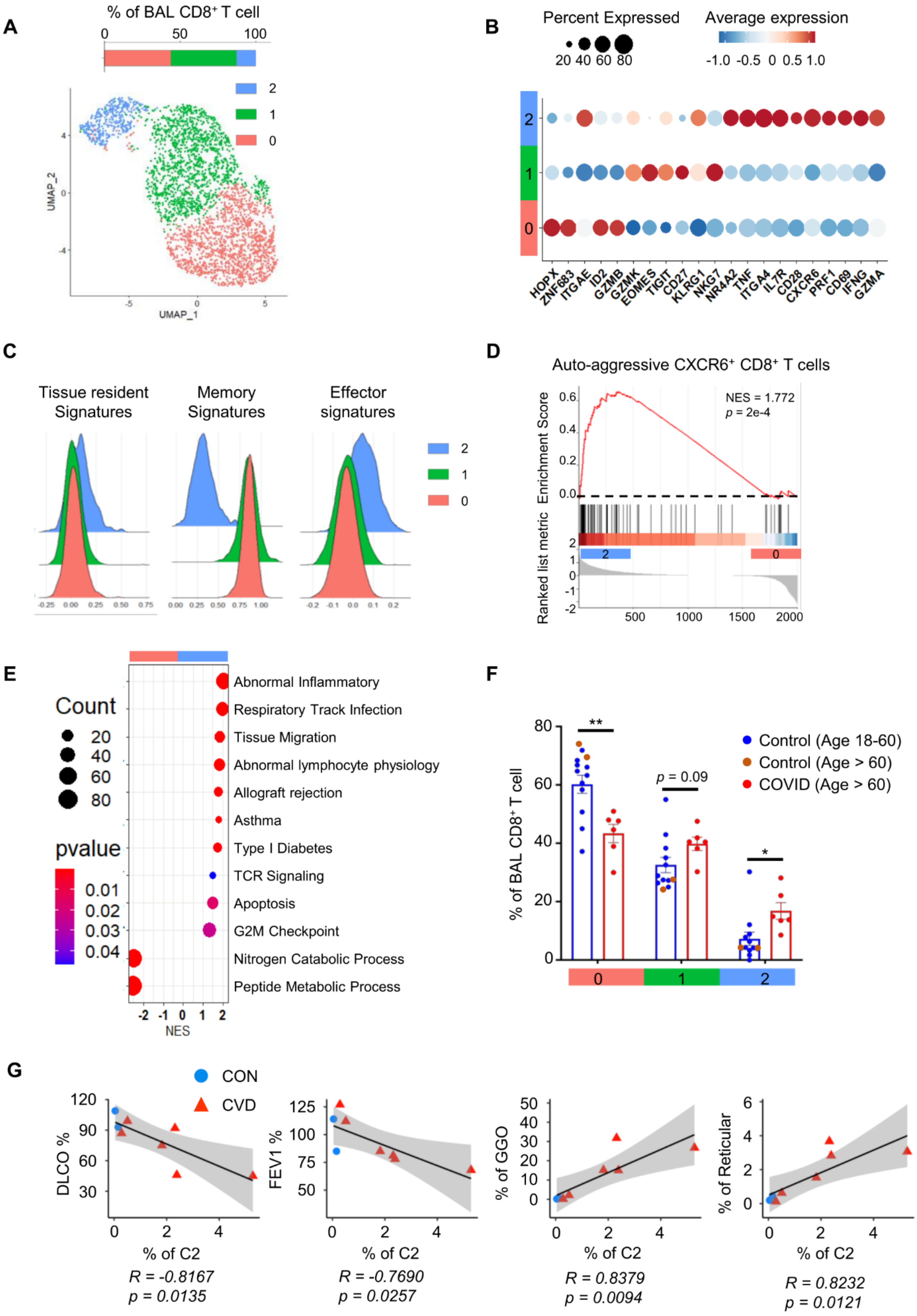
A
% of BAL CD8⁺ T cell
0 50 100
2
1
0
UMAP_2
UMAP_1
B
Percent Expressed
20 40 60 80
Average expression
-1.0 -0.5 0 0.5 1.0
HOPX ZNF683 ITGAE ID2 GZMB GZMK EOMES TIGIT CD27 KLRG1 NKG7 NR4A2 TNF ITGA4 IL7R CD28 CXCR6 PRF1 CD69 IFNG GZMA
C
Tissue resident Signatures
Memory Signatures
Effector signatures
D
Auto-aggressive CXCR6⁺ CD8⁺ T cells
NES = 1.772
p = 2e-4
Enrichment Score
Ranked list metric
E
Count
20
40
60
80
pvalue
0.01
0.02
0.03
0.04
Abnormal Inflammatory
Respiratory Track Infection
Tissue Migration
Abnormal lymphocyte physiology
Allograft rejection
Asthma
Type I Diabetes
TCR Signaling
Apoptosis
G2M Checkpoint
Nitrogen Catabolic Process
Peptide Metabolic Process
NES
F
% of BAL CD8⁺ T cell
**
p = 0.09
*
Control (Age 18-60)
Control (Age > 60)
COVID (Age > 60)
G
CON
CVD
DLCO %
FEV1 %
% of GGO
% of Reticular
% of C2
R = -0.8167
p = 0.0135
R = -0.7690
p = 0.0257
R = 0.8379
p = 0.0094
R = 0.8232
p = 0.0121

Fig. 4. scRNAseq analysis of respiratory CD8⁺ T cells from COVID-19 convalescents. **A.** BAL $CD8^+$ T cell subclusters and relative abundance revealed by scRNAseq. **B.** Signature genes expression by different BAL $CD8^+$ T cell clusters. **C.** Relative levels of tissue residency, memory and effector gene signatures in BAL $CD8^+$ T cell clusters. **D.** GSEA of auto-aggressive $CXCR6^+$ $CD8^+$ T cells between cluster 0 and 2. **E**. Different pathways enriched in cluster 2 compared to cluster 0 of BAL $CD8^+$ T cells. **F**. Relative abundance of BAL $CD8^+$ T cell subclusters from control (aged-matched control recruited in this study and age-unmatched control from GSE151928 (*33*)) or COVID-19 convalescent donors. **G.** Correlation of DLCO, FEV1, GGO and Reticular region with % of cluster 2 $CXCR6^{hi}$ $CD8^+$ T cells in the BAL cells of age-matched control and COVID-19 convalescents.
