# Supplementary material for: Immune signatures underlying post-acute COVID-19 lung sequelae
Source: Sci Immunol. 2021 Nov 12;6(65):eabk1741. doi: 10.1126/sciimmunol.abk1741 (PMC8763087; doi:10.1126/sciimmunol.abk1741)
Supplement: Supplementary file 2 — Figs. S1 to S16 [file sciimmunol.abk1741_sm.pdf]

Science Immunology
AAAS

Supplementary Materials for

Immune signatures underlying post-acute COVID-19 lung sequelae

I. S. Cheon *et al.*

Corresponding author: J. Sun, sun.jie@mayo.edu, js6re@virginia.edu

Sci. Immunol. **6**, eabk1741 (2021)
DOI: 10.1126/sciimmunol.abk1741

The PDF file includes:

Figs. S1 to S16

Other Supplementary Material for this manuscript includes the following:

Data files S1 to S4

Figure S1

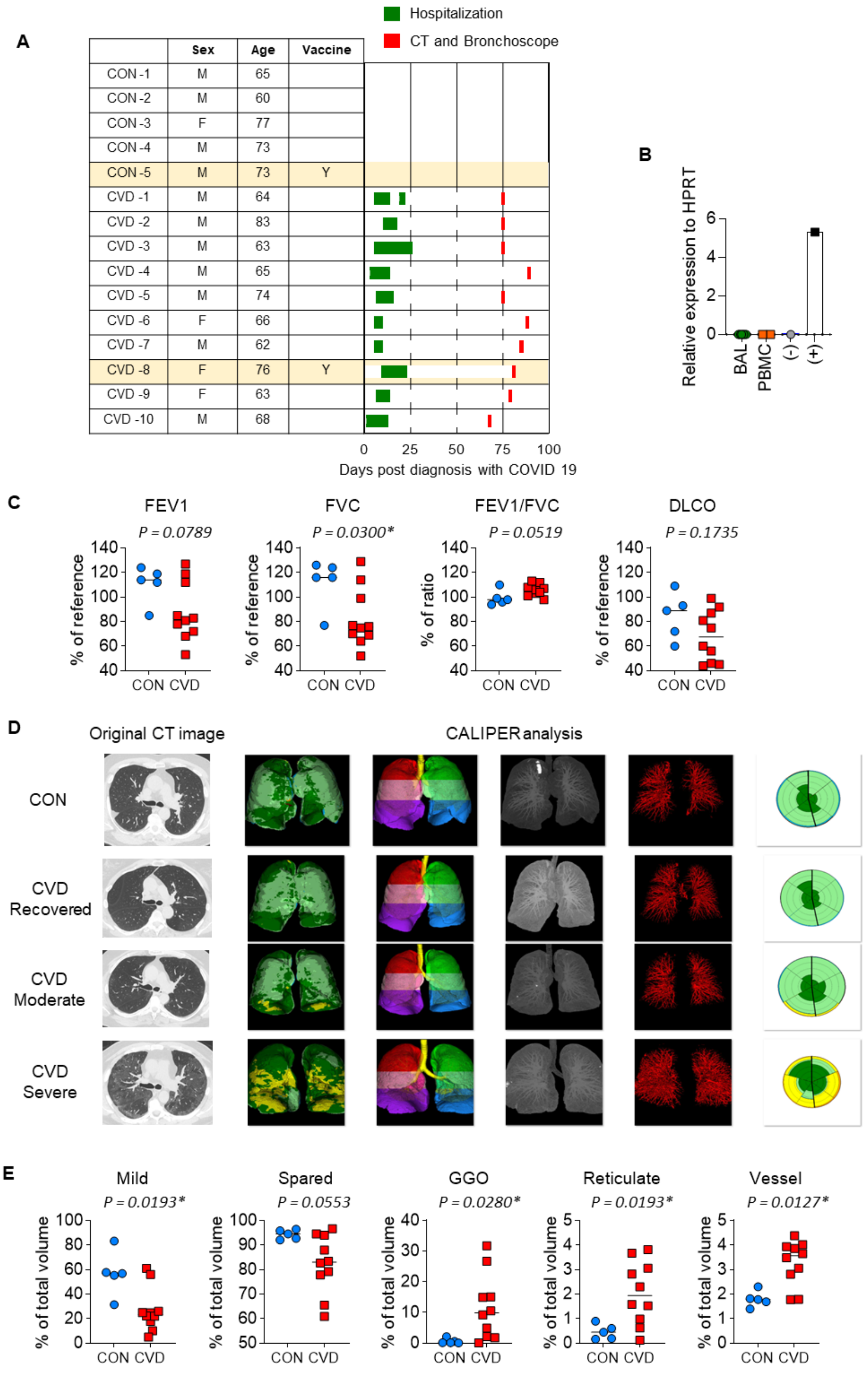

	Sex	Age	Vaccine
CON -1	M	65	
CON -2	M	60	
CON -3	F	77	
CON -4	M	73	
CON -5	M	73	Y
CVD -1	M	64	
CVD -2	M	83	
CVD -3	M	63	
CVD -4	M	65	
CVD -5	M	74	
CVD -6	F	66	
CVD -7	M	62	
CVD -8	F	76	Y
CVD -9	F	63	
CVD -10	M	68	

Fig. S1. Functional and pathological characterization of lung sequelae in aged COVID-19 convalescents. A. Cohort information of aged healthy control and COVID-19 convalescents. **B.** RT-PCR of SARS-CoV-2 N1 gene in the BAL or PBMC of COVID-19 convalescents. (-), negative control of uninfected human alveolar macrophages (AMs). (+), positive control of SARS-CoV-2 infected AMs. **C.** Pulmonary function parameters of control or COVID-19 convalescents. **D.** Representative lung CT image and CALIPER analysis (Green/Dark Green, "spared/relatively spared"; Yellow, "GGO"; Orange, "Reticular/Consolidation"). **E.** Lung pathological parameters of control or COVID-19 convalescents. **C and E.** Statistical significance was calculated using Mann-Whitney test. * $p < 0.05$.

Figure S2

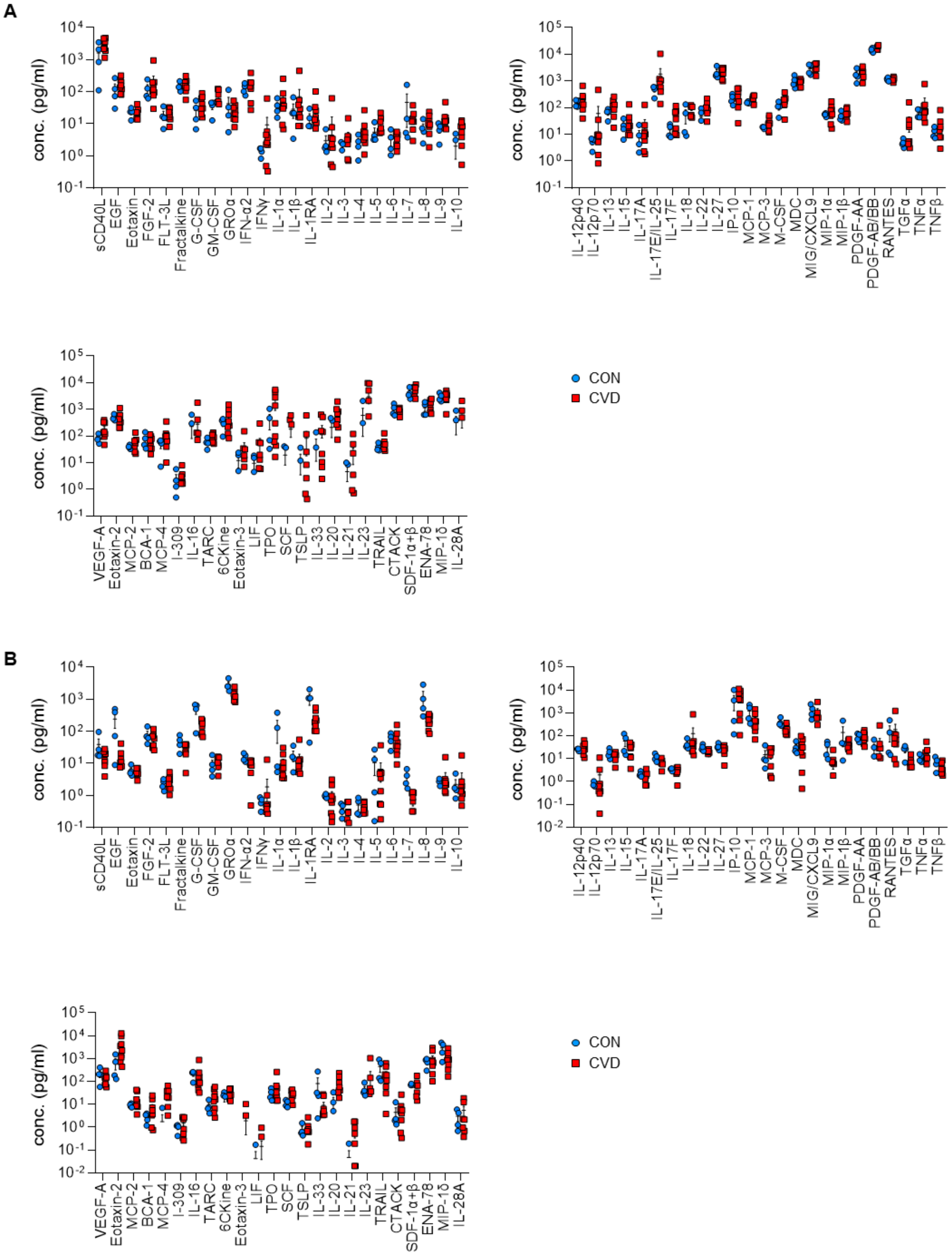
A
conc. (pg/ml)
10^{4} 10^{3} 10^{2} 10^{1} 10^{0} 10^{-1}
sCD40L EGF Eotaxin FGF-2 FLT-3L Fractalkine G-CSF GM-CSF GROα IFN-α2 IFNγ IL-1α IL-1β IL-1RA IL-2 IL-3 IL-4 IL-5 IL-6 IL-7 IL-8 IL-9 IL-10
conc. (pg/ml)
10^{5} 10^{4} 10^{3} 10^{2} 10^{1} 10^{0} 10^{-1}
IL-12p40 IL-12p70 IL-13 IL-15 IL-17A IL-17E/IL-25 IL-17F IL-18 IL-22 IL-27 IP-10 MCP-1 MCP-3 M-CSF MDC MIG/CXCL9 MIP-1α MIP-1β PDGF-AA PDGF-AB/BB RANTES TGFα TNFα TNFβ
conc. (pg/ml)
10^{5} 10^{4} 10^{3} 10^{2} 10^{1} 10^{0} 10^{-1}
VEGF-A Eotaxin-2 MCP-2 BCA-1 MCP-4 I-309 IL-16 TARC 6CKine Eotaxin-3 LIF TPO SCF TSLP IL-33 IL-20 IL-21 IL-23 TRAIL CTACK SDF-1α+β ENA-78 MIP-1δ IL-28A
CON
CVD
B
conc. (pg/ml)
10^{4} 10^{3} 10^{2} 10^{1} 10^{0} 10^{-1}
sCD40L EGF Eotaxin FGF-2 FLT-3L Fractalkine G-CSF GM-CSF GROα IFN-α2 IFNγ IL-1α IL-1β IL-1RA IL-2 IL-3 IL-4 IL-5 IL-6 IL-7 IL-8 IL-9 IL-10
conc. (pg/ml)
10^{5} 10^{4} 10^{3} 10^{2} 10^{1} 10^{0} 10^{-1} 10^{-2}
IL-12p40 IL-12p70 IL-13 IL-15 IL-17A IL-17E/IL-25 IL-17F IL-18 IL-22 IL-27 IP-10 MCP-1 MCP-3 M-CSF MDC MIG/CXCL9 MIP-1α MIP-1β PDGF-AA PDGF-AB/BB RANTES TGFα TNFα TNFβ
conc. (pg/ml)
10^{5} 10^{4} 10^{3} 10^{2} 10^{1} 10^{0} 10^{-1} 10^{-2}
VEGF-A Eotaxin-2 MCP-2 BCA-1 MCP-4 I-309 IL-16 TARC 6CKine Eotaxin-3 LIF TPO SCF TSLP IL-33 IL-20 IL-21 IL-23 TRAIL CTACK SDF-1α+β ENA-78 MIP-1δ IL-28A
CON
CVD

Fig. S2. Inflammatory cytokine levels in the blood and BAL of COVID-19 convalescents. A. Cytokines were detected by multiplex cytokine assay in plasma. **B.** Cytokines were detected by multiplex assay in concentrated BAL. Statistical significance was calculated using Mann-Whitney test.

Figure S3

A BAL

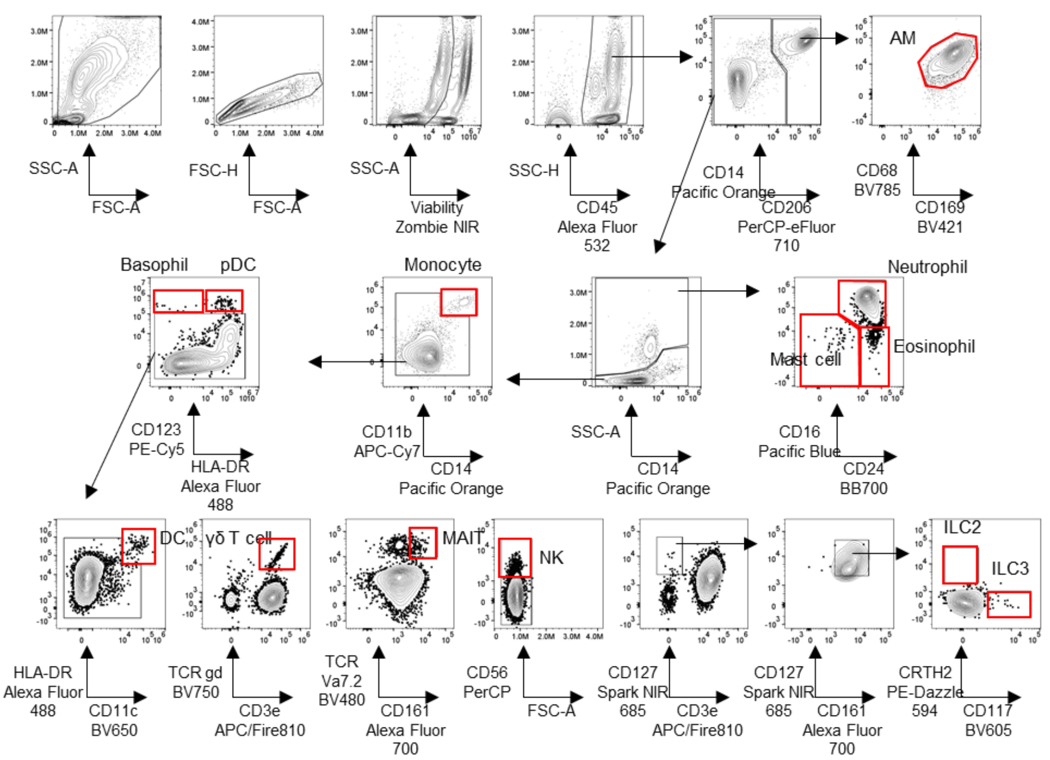

B Blood

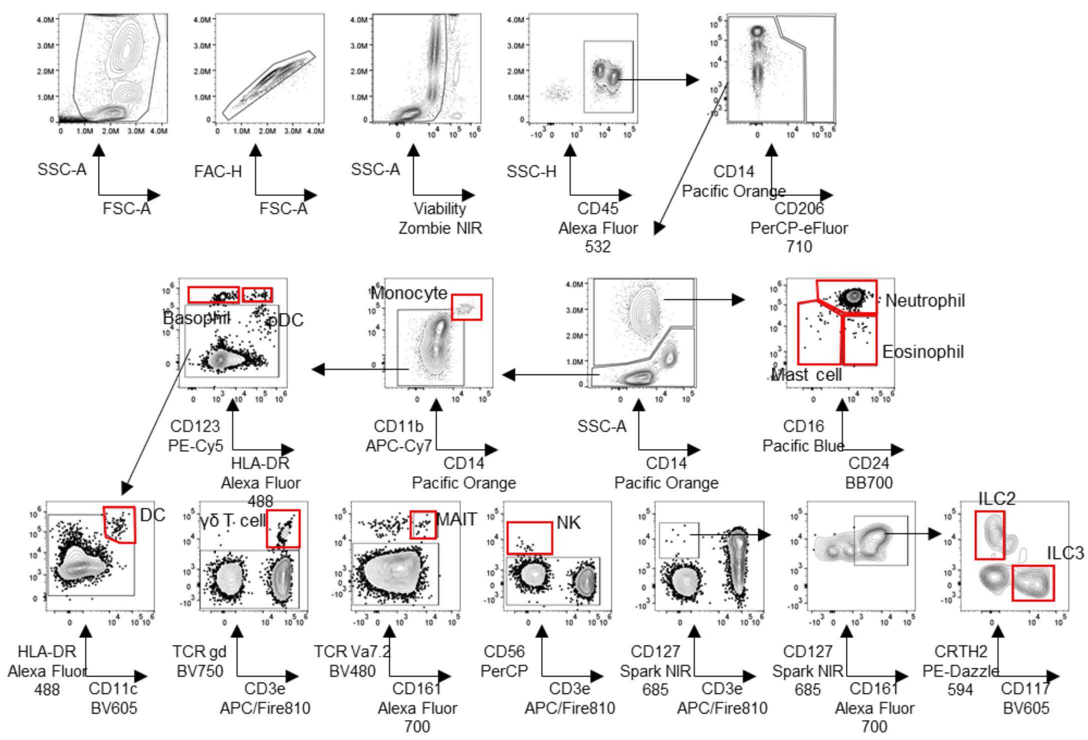

Fig. S3. Flow cytometric analysis and gating strategy of innate cells. A. Gating strategy of innate cells in human BAL. Flow cytometry was performed on BAL cells. **B.** Gating strategy of innate cells in human blood.

Figure S4

A BAL

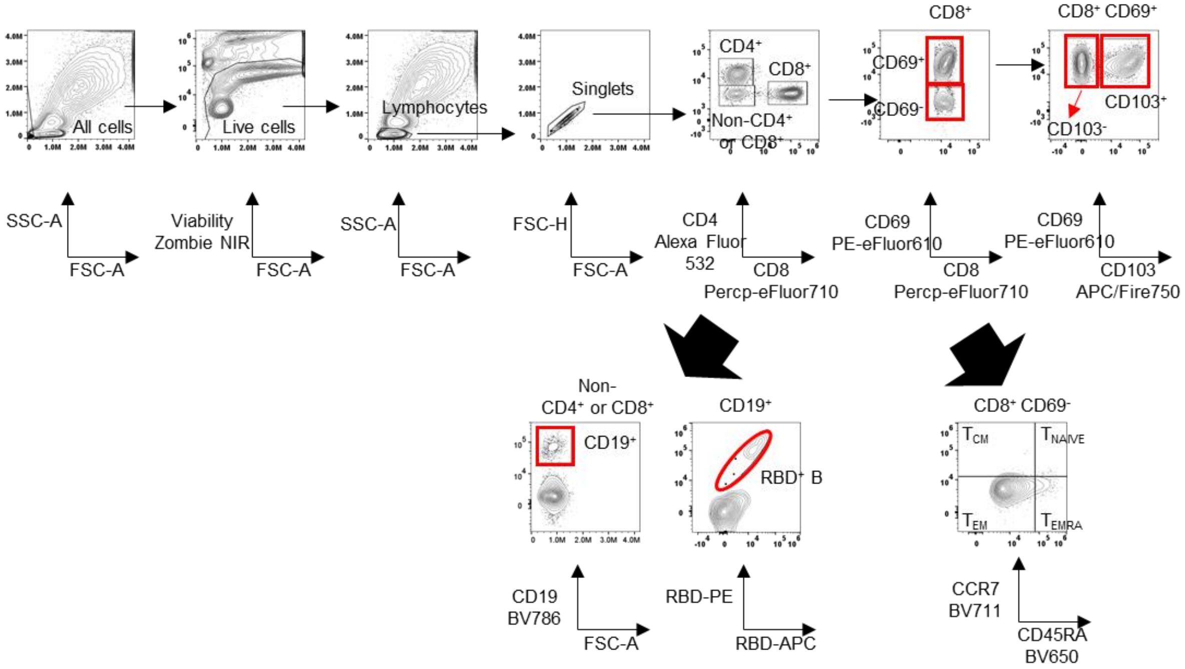

B Blood

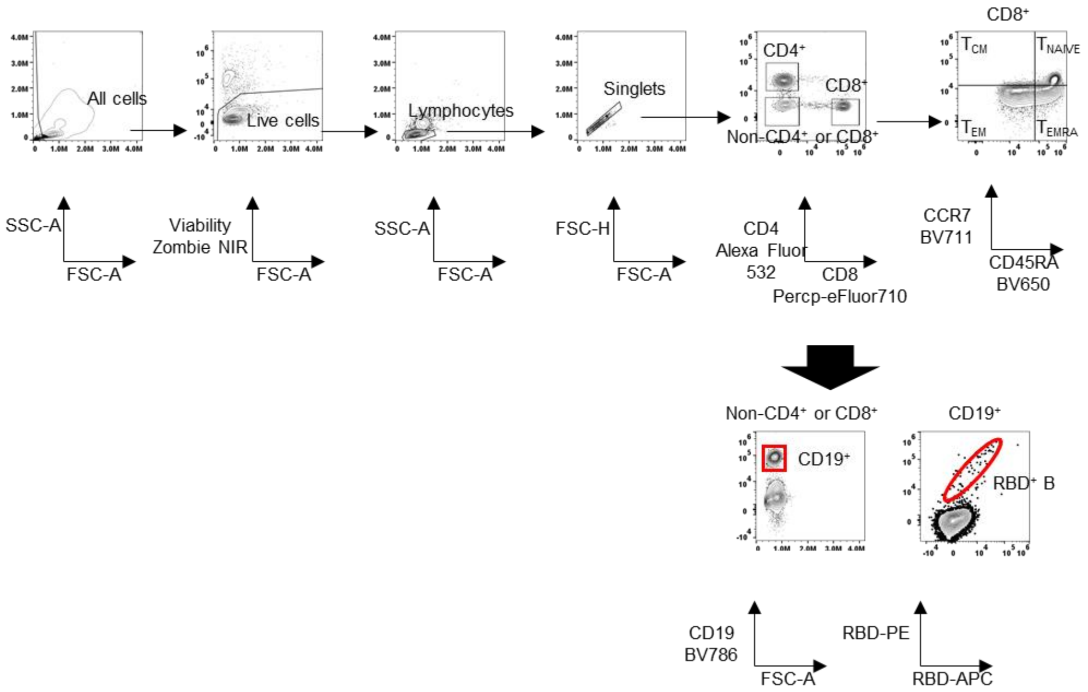

Fig. S4. Flow cytometric analysis and gating strategy of lymphocytes. A. Gating strategy of adaptive cells in human BAL. Flow cytometry was performed on BAL cells. **B.** Gating strategy of adaptive cells in human PBMC.

Figure S5

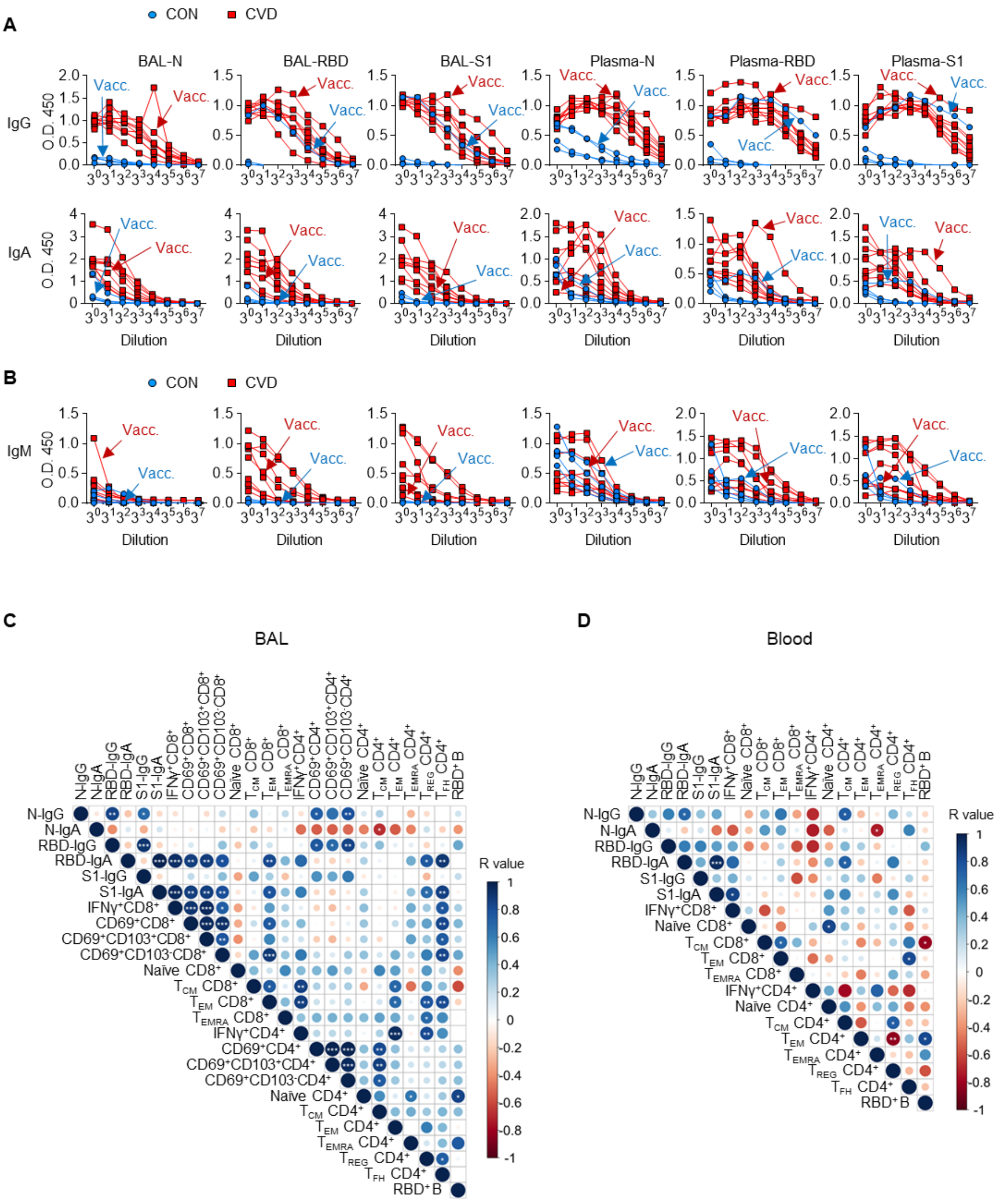

A
CON
CVD
BAL-N
BAL-RBD
BAL-S1
Plasma-N
Plasma-RBD
Plasma-S1
IgG
IgA
O.D. 450
Vacc.
Dilution
B
CON
CVD
IgM
O.D. 450
Vacc.
Dilution
C
BAL
D
Blood
R value
N-IgG
N-IgA
RBD-IgG
RBD-IgA
S1-IgG
S1-IgA
IFNγ⁺CD8⁺
CD69⁺CD8⁺
CD69⁺CD103⁺CD8⁺
CD69⁺CD103⁻CD8⁺
Naïve CD8⁺
T_CM CD8⁺
T_EM CD8⁺
T_EMRA CD8⁺
IFNγ⁺CD4⁺
CD69⁺CD4⁺
CD69⁺CD103⁺CD4⁺
CD69⁺CD103⁻CD4⁺
Naïve CD4⁺
T_CM CD4⁺
T_EM CD4⁺
T_EMRA CD4⁺
T_REG CD4⁺
T_FH CD4⁺
RBD⁺B

Fig. S5. Characterization of respiratory or blood humoral responses at the convalescent stage. A. SARS-CoV-2 (N, RBD or S1) specific IgG (top row), IgA (bottom row) responses in BAL or plasma. **B.** SARS-CoV-2 (N, RBD or S1) specific IgM responses in BAL or plasma. Blue arrow indicates vaccinated control subject and red arrow indicates vaccinate COVID-19 convalescent. **C, D.** Correlation matrix between viral specific antibodies and lymphocyte subsets in BAL (**C**) or blood (**D**). **C, D.** Pearson correlation, r values are indicated by color and circle size. Significant correlations were indicated by white asterisks.

Figure S6

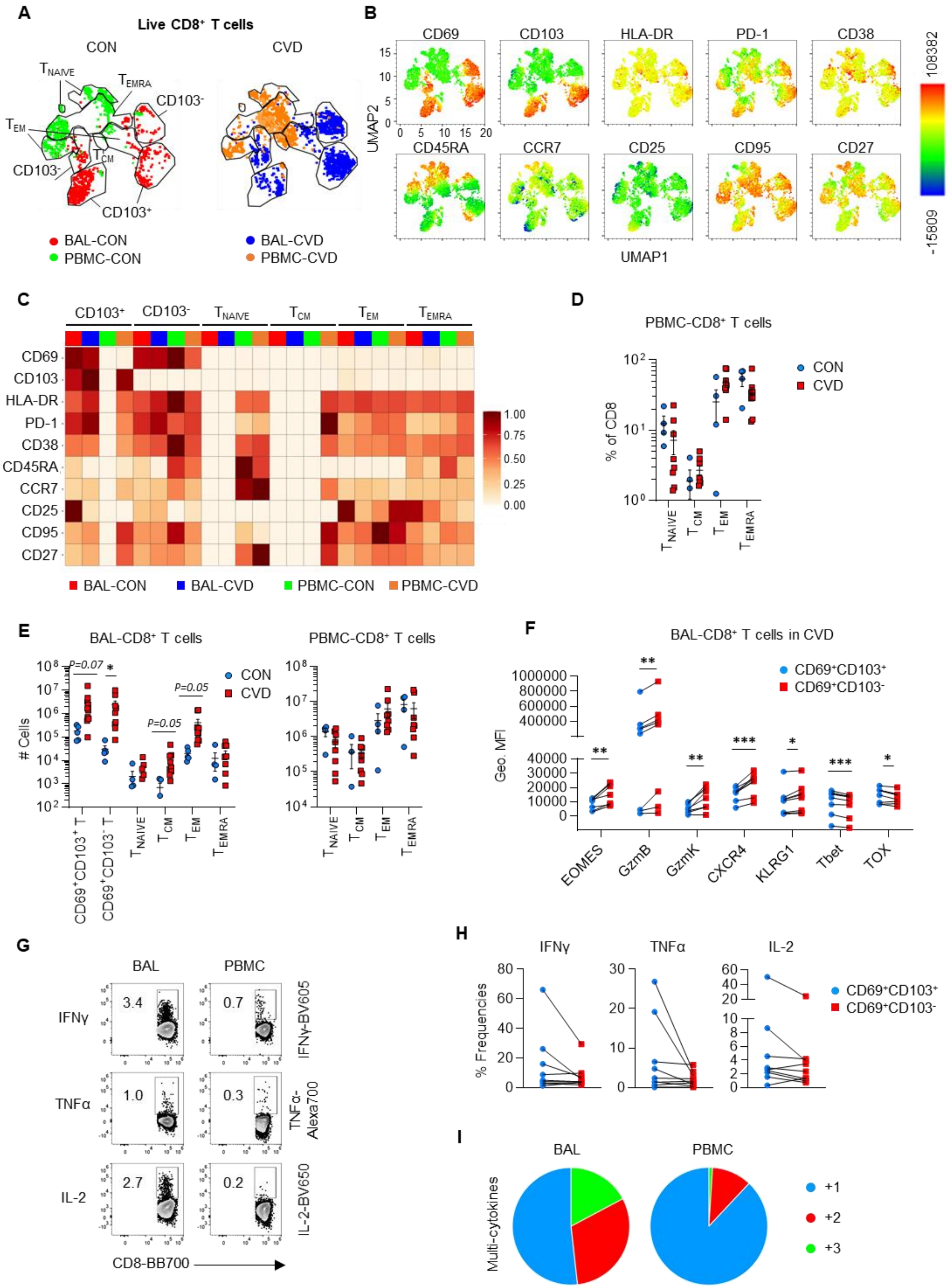
A
Live CD8+ T cells
CON
CVD
BAL-CON
PBMC-CON
BAL-CVD
PBMC-CVD
B
CD69
CD103
HLA-DR
PD-1
CD38
CD45RA
CCR7
CD25
CD95
CD27
UMAP2
UMAP1
108382
-15809
C
D
PBMC-CD8+ T cells
% of CD8
E
BAL-CD8+ T cells
PBMC-CD8+ T cells
Cells
F
BAL-CD8+ T cells in CVD
CD69+CD103+
CD69+CD103-
Geo. MFI
EOMES
GzmB
GzmK
CXCR4
KLRG1
Tbet
TOX
G
BAL
PBMC
IFNγ
TNFα
IL-2
IFNγ-BV605
TNFα-Alexa700
IL-2-BV650
CD8-BB700
H
% Frequencies
I
Multi-cytokines
+1
+2
+3

Fig. S6. Phenotypes of $CD8^+$ T cell in control and COVID-19 convalescents. A. UMAP analysis of the different sub-populations of $CD8^+$ T cells based on BAL control subjects (CON), BAL COVID-19 convalescents (CVD), PBMC CON and PBMC CVD groups. **B.** Surface marker expression of merged $CD8^+$ T cells. **C.** Heat map of different marker expression in $CD8^+$ T cell subsets. **D.** % of indicated populations in PBMC $CD8^+$ T cells. **E.** Cell numbers of BAL (left) and PBMC (right) $CD8^+$ T cell subtypes. **F.** Expression of indicated molecules on BAL $CD8^+$ T_{RMs} including ($CD103^+$ and $CD103^-$) in CVD groups. **G.** Representative flow cytometry plots on cytokine expression by $CD8^+$ cells stimulated by SARS-CoV-2 peptide pools for 6 hrs in CVD groups. **H.** Expression of cytokines on BAL $CD8^+$ T_{RMs} stimulated by SARS-CoV-2 peptide pools. **I.** Pie chart graph on % of single, double or triple $cytokine^+$ $CD8^+$ T cells in the BAL or PBMC Statistical significance was calculated using Mann-Whitney test (**D** and **E**) or paired t-test (**F** and **H**). * < 0.05, ** < 0.01 and *** < 0.001.

Figure S7

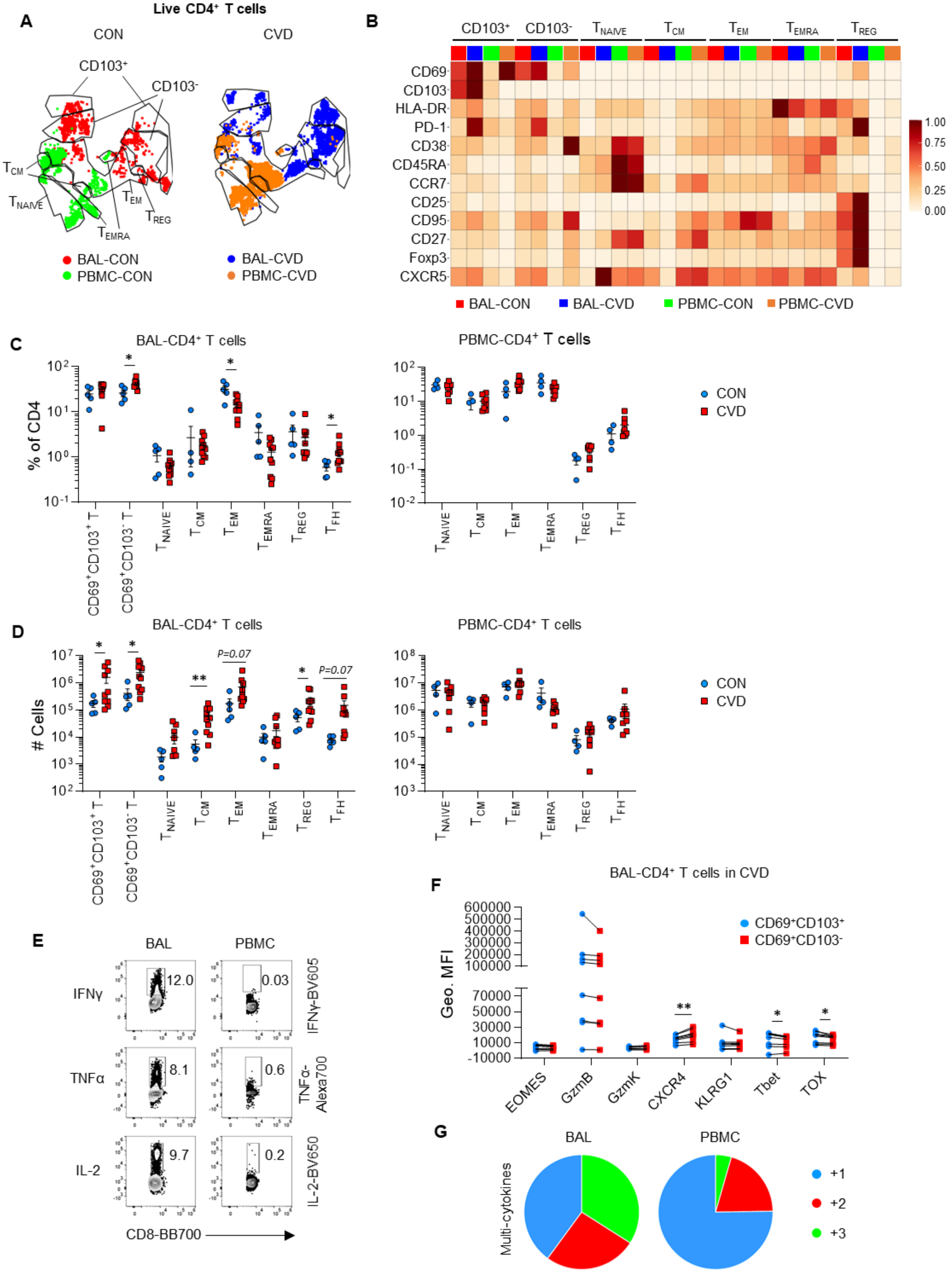

A
Live CD4+ T cells
CON
CVD
CD103+
CD103-
TCM
TNAIVE
TEM
TREG
TEMRA
BAL-CON
PBMC-CON
BAL-CVD
PBMC-CVD
B
CD103+
CD103-
TNAIVE
TCM
TEM
TEMRA
TREG
CD69
CD103
HLA-DR
PD-1
CD38
CD45RA
CCR7
CD25
CD95
CD27
Foxp3
CXCR5
1.00
0.75
0.50
0.25
0.00
BAL-CON
BAL-CVD
PBMC-CON
PBMC-CVD
C
BAL-CD4+ T cells
PBMC-CD4+ T cells
% of CD4
CON
CVD
D
BAL-CD4+ T cells
PBMC-CD4+ T cells
# Cells
P=0.07
P=0.07
E
BAL
PBMC
IFNγ
TNFα
IL-2
12.0
0.03
8.1
0.6
9.7
0.2
IFNγ-BV605
TNFα-Alexa700
IL-2-BV650
CD8-BB700
F
BAL-CD4+ T cells in CVD
Geo. MFI
CD69+CD103+
CD69+CD103-
EOMES
GzmB
GzmK
CXCR4
KLRG1
Tbet
TOX
G
BAL
PBMC
Multi-cytokines
+1
+2
+3

Fig. S7. Phenotypes of $CD4^+$ T cell in control and COVID-19 convalescents. A. UMAP analysis of the different sub-populations of $CD4^+$ T cells based on BAL CON, BAL CVD, PBMC CON and PBMC CVD groups. **B.** Heat map of different marker expression in $CD4^+$ T cell subsets. **C, D.** Frequencies (**C**) or cell numbers (**D**) of indicated $CD4^+$ T cells in BAL or PBMC. **E.** Representative flow cytometry plots on cytokine expression by $CD4^+$ cells stimulated by SARS-CoV-2 peptide pools for 6 in CVD groups**. F.** Expression of indicated molecules in BAL $CD4^+$ T_{RM} including ($CD103^+$ and $CD103^-$) in CVD groups. **G.** Pie chart graph on % of single, double or triple $cytokine^+$ $CD4^+$ T cells in the BAL or PBMC. Statistical significance was calculated using Mann-Whitney test (**C** and **D**) or paired t-test (**F**). * < 0.05 and ** < 0.01.

Figure S8

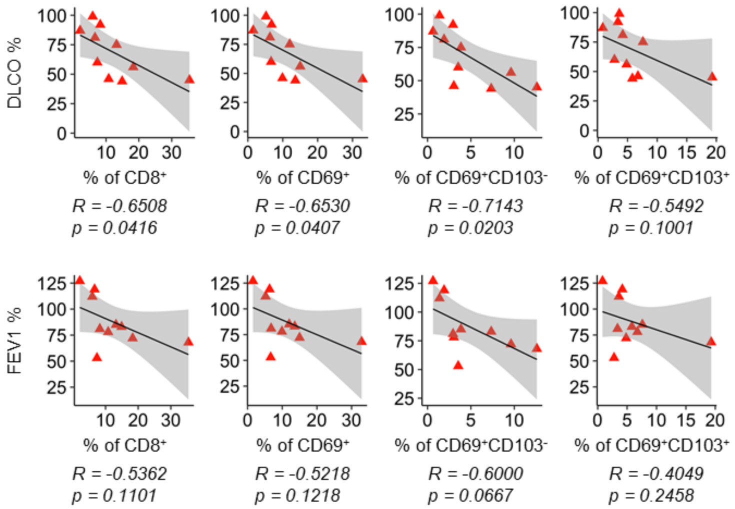

Fig. S8. Correlation between lung function parameters with BAL $CD8^+$ T cell subsets. Correlation of DLCO and FEV1 with % of total $CD8^+$, $CD69^+$ $CD8^+$, $CD69^+CD103^-$ $CD8^+$ or $CD69^+CD103^+$ $CD8^+$ T cells in the BAL of COVID-19 convalescents.

Figure S9

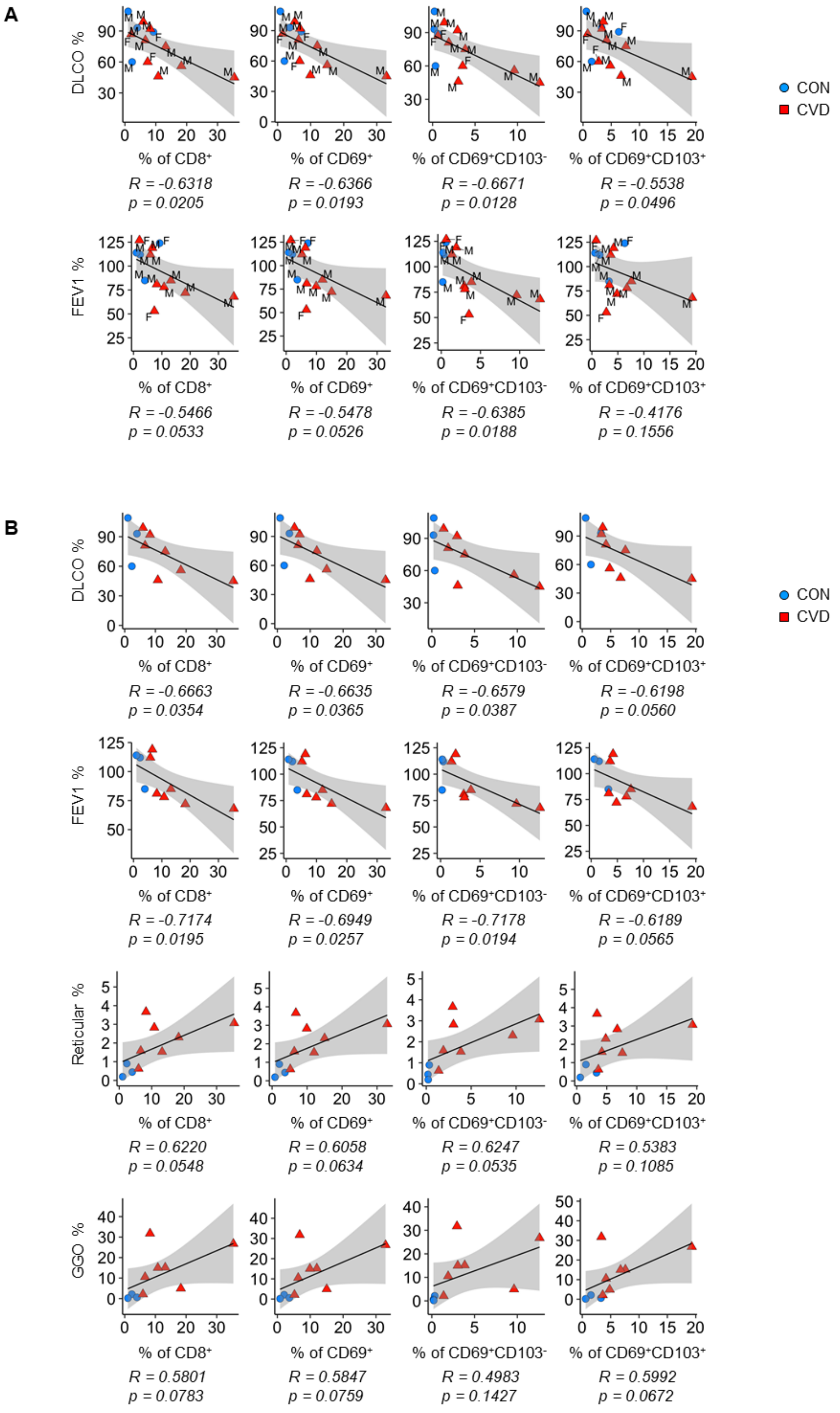

A
DLCO %
FEV1 %
% of CD8⁺
% of CD69⁺
% of CD69⁺CD103⁻
% of CD69⁺CD103⁺
CON
CVD
R = -0.6318
p = 0.0205
R = -0.6366
p = 0.0193
R = -0.6671
p = 0.0128
R = -0.5538
p = 0.0496
R = -0.5466
p = 0.0533
R = -0.5478
p = 0.0526
R = -0.6385
p = 0.0188
R = -0.4176
p = 0.1556
B
DLCO %
FEV1 %
Reticular %
GGO %
CON
CVD
R = -0.6663
p = 0.0354
R = -0.6635
p = 0.0365
R = -0.6579
p = 0.0387
R = -0.6198
p = 0.0560
R = -0.7174
p = 0.0195
R = -0.6949
p = 0.0257
R = -0.7178
p = 0.0194
R = -0.6189
p = 0.0565
R = 0.6220
p = 0.0548
R = 0.6058
p = 0.0634
R = 0.6247
p = 0.0535
R = 0.5383
p = 0.1085
R = 0.5801
p = 0.0783
R = 0.5847
p = 0.0759
R = 0.4983
p = 0.1427
R = 0.5992
p = 0.0672

Fig. S9. Correlation between lung function parameters with BAL lymphocytes in non-vaccinated subjects. A. Correlation of DLCO and FEV1 with % of total $CD8^+$, $CD69^+$ $CD8^+$, $CD69^+CD103^-$ $CD8^+$ or $CD69^+CD103^+$ $CD8^+$ T cells in the BAL of non-vaccinated control and COVID-19 convalescents. M: male, F: female. **B.** Correlation of DLCO, FEV1, Reticular and GGO with % of total $CD8^+$, $CD69^+$ $CD8^+$, $CD69^+CD103^-$ $CD8^+$ or $CD69^+CD103^+$ $CD8^+$ T cells in the BAL of non-vaccinated male control and COVID-19 convalescents.

Figure S10

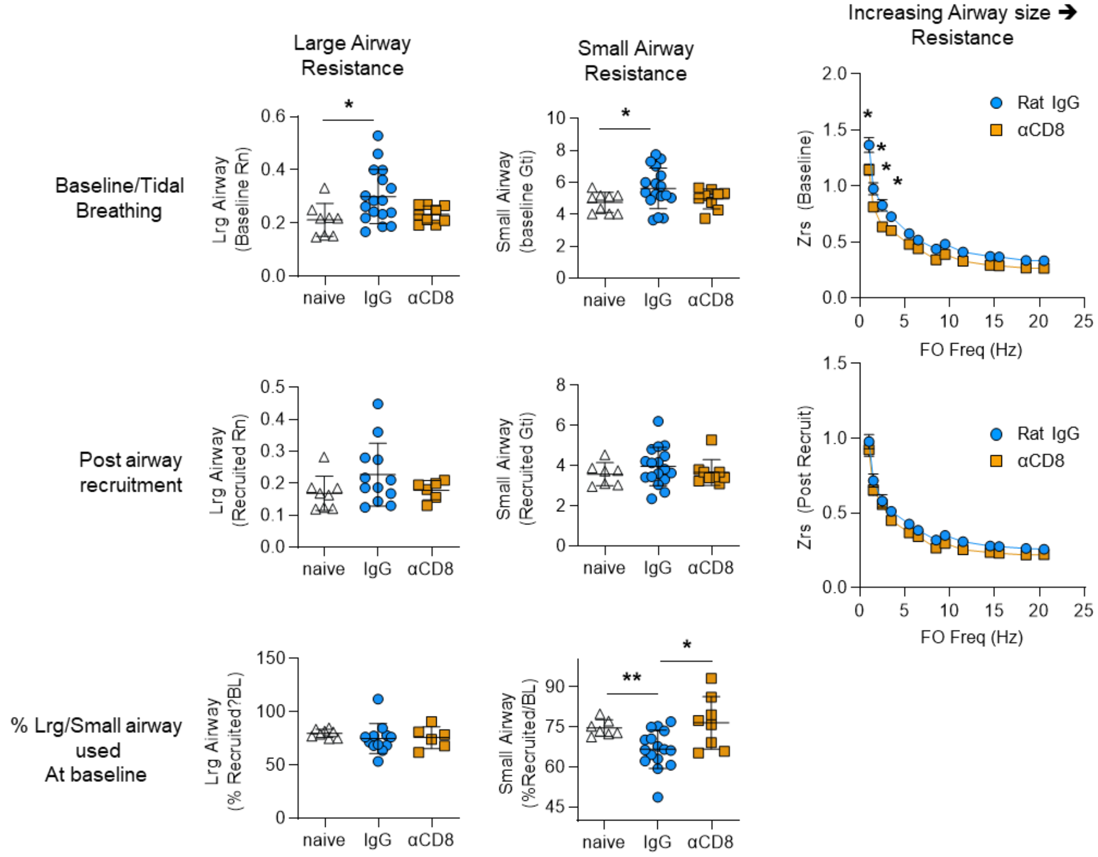

Fig. S10. CD8+ T cells are a driver of chronic lung impairment following viral pneumonia during aging. Aged mice were infected or not (Naive) as in Figure 3. 3 weeks later, mice were treated with weekly with CD8-depleting antibody (αCD8) or isotope control (Rat IgG) until 50 days post-infection. Lung function was measured via forced oscillation technique. Parameters from the constant-phase model (Rn left column, Gti middle column, Zrs right column) were measured prior (top row) to and after inflation (middle row) of the lungs to total lung capacity. The bottom row is normalized data from the top and middle rows indicating % of the total lung capacity used at baseline for each parameter by each mouse. 3 independent pooled experiments * $p < 0.05$ and ** $p < 0.01$ for each indicated multiple comparison by one-way (left and middle columns) or two-way (right column) ANOVA following Fisher's LSD test.

Figure S11

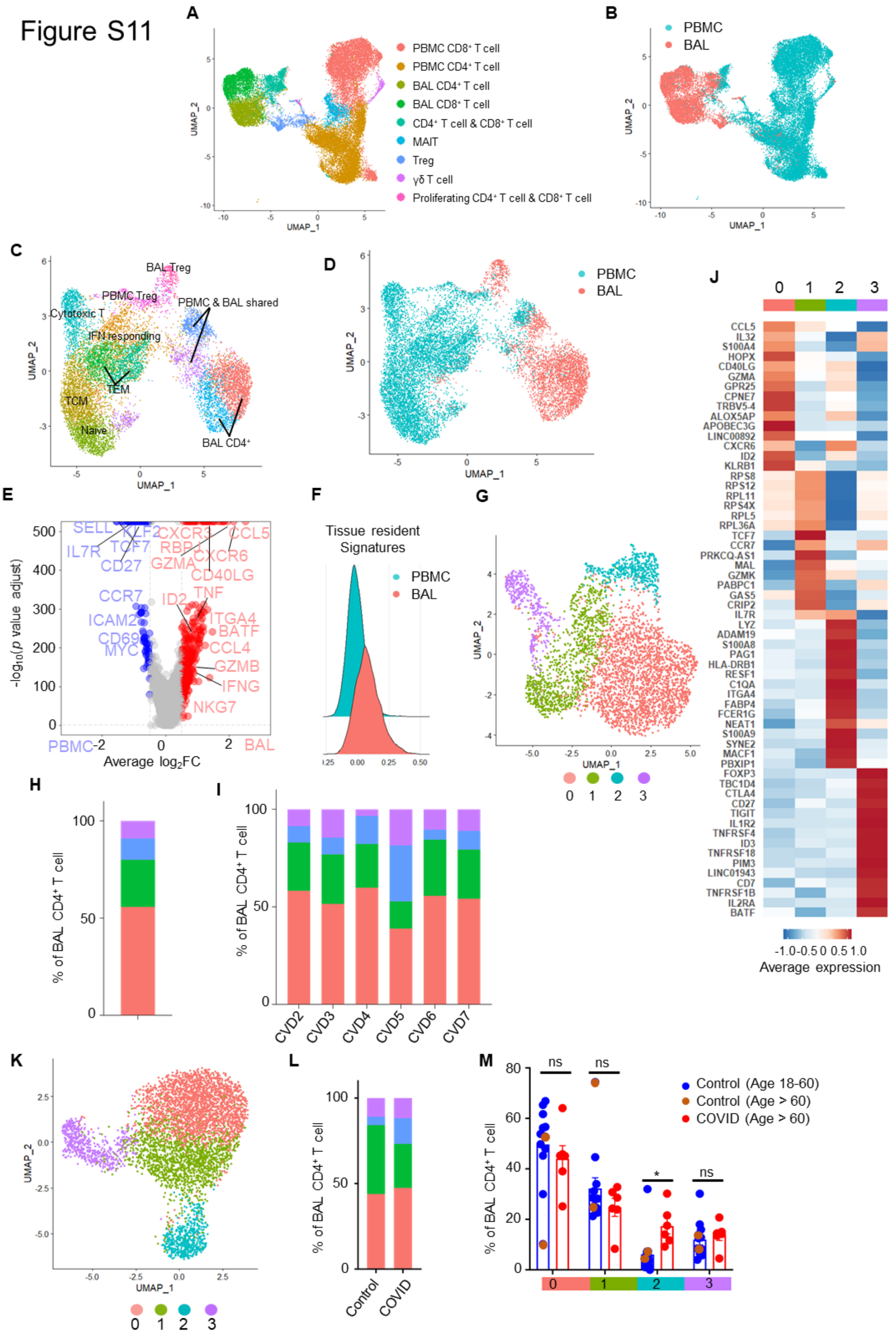

A
PBMC CD8⁺ T cell
PBMC CD4⁺ T cell
BAL CD4⁺ T cell
BAL CD8⁺ T cell
CD4⁺ T cell & CD8⁺ T cell
MAIT
Treg
γδ T cell
Proliferating CD4⁺ T cell & CD8⁺ T cell
UMAP_1
UMAP_2
B
PBMC
BAL
C
BAL Treg
PBMC Treg
PBMC & BAL shared
Cytotoxic T
IFN responding
TEM
TCM
Naive
BAL CD4⁺
D
PBMC
BAL
E
SELL
KLF2
CXCR3
CCL5
IL7R
TCF7
RBPJ
CXCR6
CD27
GZMA
CD40LG
CCR7
ID2
TNF
ICAM2
ITGA4
CD69
BATF
MYC
CCL4
GZMB
IFNG
NKG7
PBMC
BAL
Average log2FC
-log10(p value adjust)
F
Tissue resident Signatures
PBMC
BAL
G
H
% of BAL CD4⁺ T cell
I
CVD2
CVD3
CVD4
CVD5
CVD6
CVD7
J
CCL5
IL32
S100A4
HOPX
CD40LG
GZMA
GPR25
CPNE7
TRBV5-4
ALOX5AP
APOBEC3G
LINC00892
CXCR6
ID2
KLRB1
RPS8
RPS12
RPL11
RPS4X
RPL5
RPL36A
TCF7
CCR7
PRKCQ-AS1
MAL
GZMK
PABPC1
GAS5
CRIP2
IL7R
LYZ
ADAM19
S100A8
PAG1
HLA-DRB1
RESF1
C1QA
ITGA4
FABP4
FCER1G
NEAT1
S100A9
SYNE2
MACF1
PBXIP1
FOXP3
TBC1D4
CTLA4
CD27
TIGIT
IL1R2
TNFRSF4
ID3
TNFRSF18
PIM3
LINC01943
CD7
TNFRSF1B
IL2RA
BATF
-1.0-0.5 0 0.5 1.0
Average expression
K
L
Control
COVID
M
ns
ns
*
ns
Control (Age 18-60)
Control (Age > 60)
COVID (Age > 60)

Fig. S11. scRNA-seq of respiratory and circulating T cells. $CD3^+$ T cells from BAL or PBMC were enriched with magnetic beads and analyzed with scRNA-seq. **A.** UMAP plot visualization of T cell clusters from BAL and PBMC of COVID-19 convalescents (n=6). **B.** UMAP representation from (**A**) colored based on T cells source. **C.** UMAP plot visualization of BAL and PBMC $CD4^+$ T cell clusters (n=6). **D.** UMAP representation from (**C**) colored based on T cells source. **E.** Volcano plot showing the differentially expressed genes between BAL and PBMC $CD4^+$ T cells. **F.** Expression of the indicated gene signature is elevated between BAL and PBMC $CD4^+$ T cells. **G.** UMAP plot showing BAL $CD4^+$ T cell clusters. **H.** Cell frequency of each cluster in total BAL $CD4^+$ T cells. **I.** The percentage of each cluster in total BAL $CD4^+$ T cells divided by individual donor. **J.** Heatmap shows the relative expression of the 15 most differentially expressed genes for each cluster of BAL $CD4^+$ T cells. **K.** BAL $CD4^+$ T cell subclusters from control and COVID-19 convalescent donors revealed by scRNA-seq. **L** and **M,** Average (**L**) and individually (**M**) relative abundance of BAL $CD4^+$ T cell subclusters from control or COVID-19 convalescent donors.

Figure S12

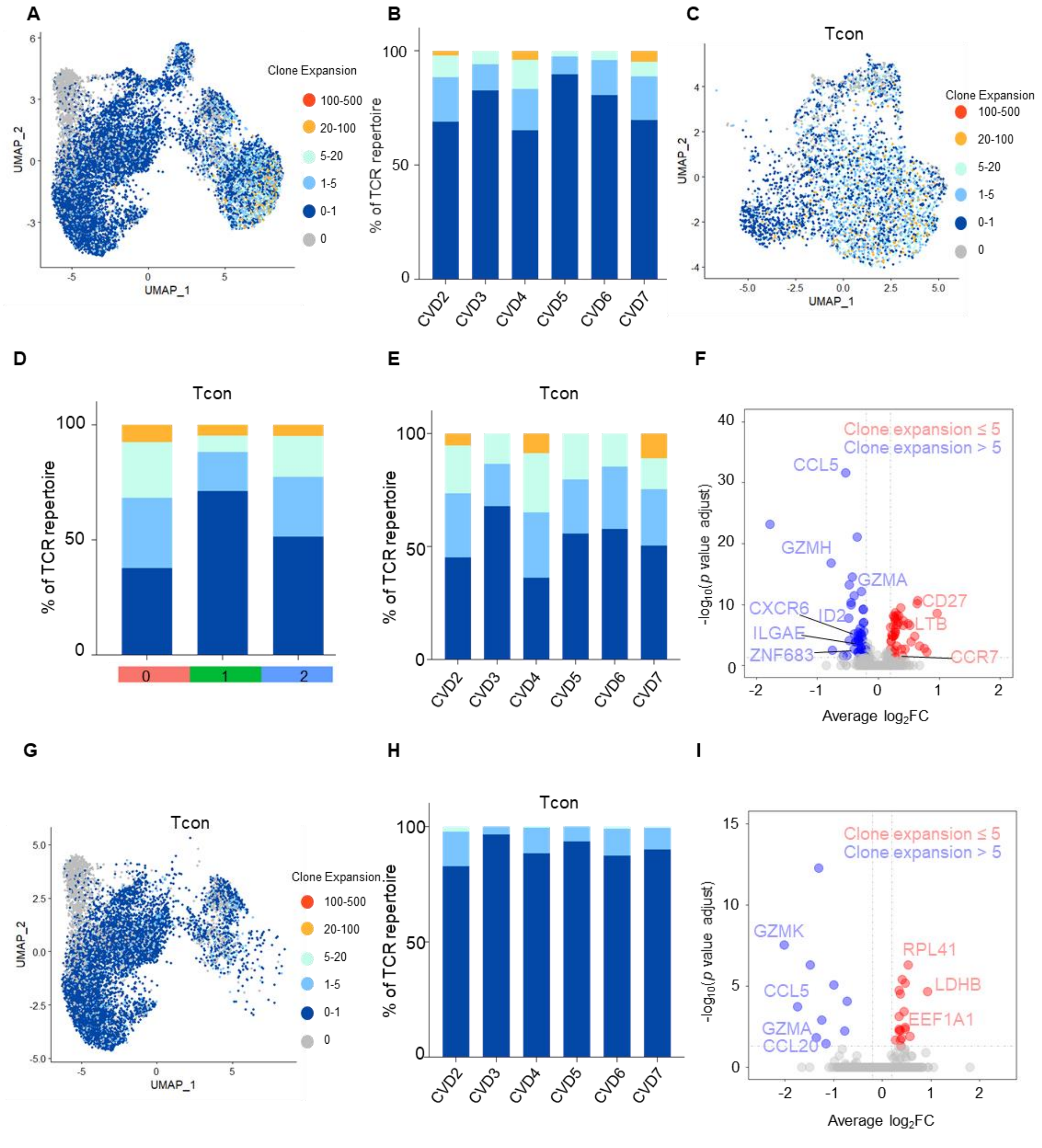
A
B
C
D
E
F
G
H
I
Clone Expansion
100-500
20-100
5-20
1-5
0-1
0
UMAP_1
UMAP_2
% of TCR repertoire
CVD2
CVD3
CVD4
CVD5
CVD6
CVD7
Tcon
Clone expansion ≤ 5
Clone expansion > 5
CCL5
GZMH
GZMA
CXCR6
ID2
ILGAE
ZNF683
CD27
LTB
CCR7
GZMK
RPL41
LDHB
EEF1A1
CCL20
Average log2FC
-log10(p value adjust)

Fig. S12. scTCR-seq of respiratory and circulating $CD4^+$ T cells in aged COVID-19 convalescents. $CD3^+$ T cells from BAL or PBMC were enriched with magnificent beads, and analyzed with scTCR-seq. **A.** UMAP plot visualization of TCR clone expansion level from BAL and PBMC $CD4^+$ T cells (n=6). **B.** The percentage of each level of TCR clone expansion in total $CD4^+$ T cells from both PBMC and BAL divided by individual donor. **C.** UMAP plot visualization of TCR clone expansion level from BAL $CD4^+$ conventional T cells (Tcon cells). **D.** The level of TCR clone expansion percentage in individual clusters of BAL $CD4^+$ Tcon cells. **E.** The level of TCR clone expansion percentage in BAL $CD4^+$ Tcon cells divided by individual donor. **F.** Volcano plot showing the differentially expressed genes between TCR clone high expanded (clone > 5) and low expanded (clone ≤ 5) BAL $CD4^+$ Tcon cells. **G.** UMAP plot visualization of TCR clone expansion level from PBMC $CD4^+$ Tcon cells. **H.** The level of TCR clone expansion percentage in PBMC $CD4^+$ Tcon cells divided by the individual donor. **I.** Volcano plot showing the differentially expressed genes between TCR clone high expanded (clone > 5) and low expanded (clone ≤ 5) PBMC $CD4^+$ Tcon cells.

Figure S13

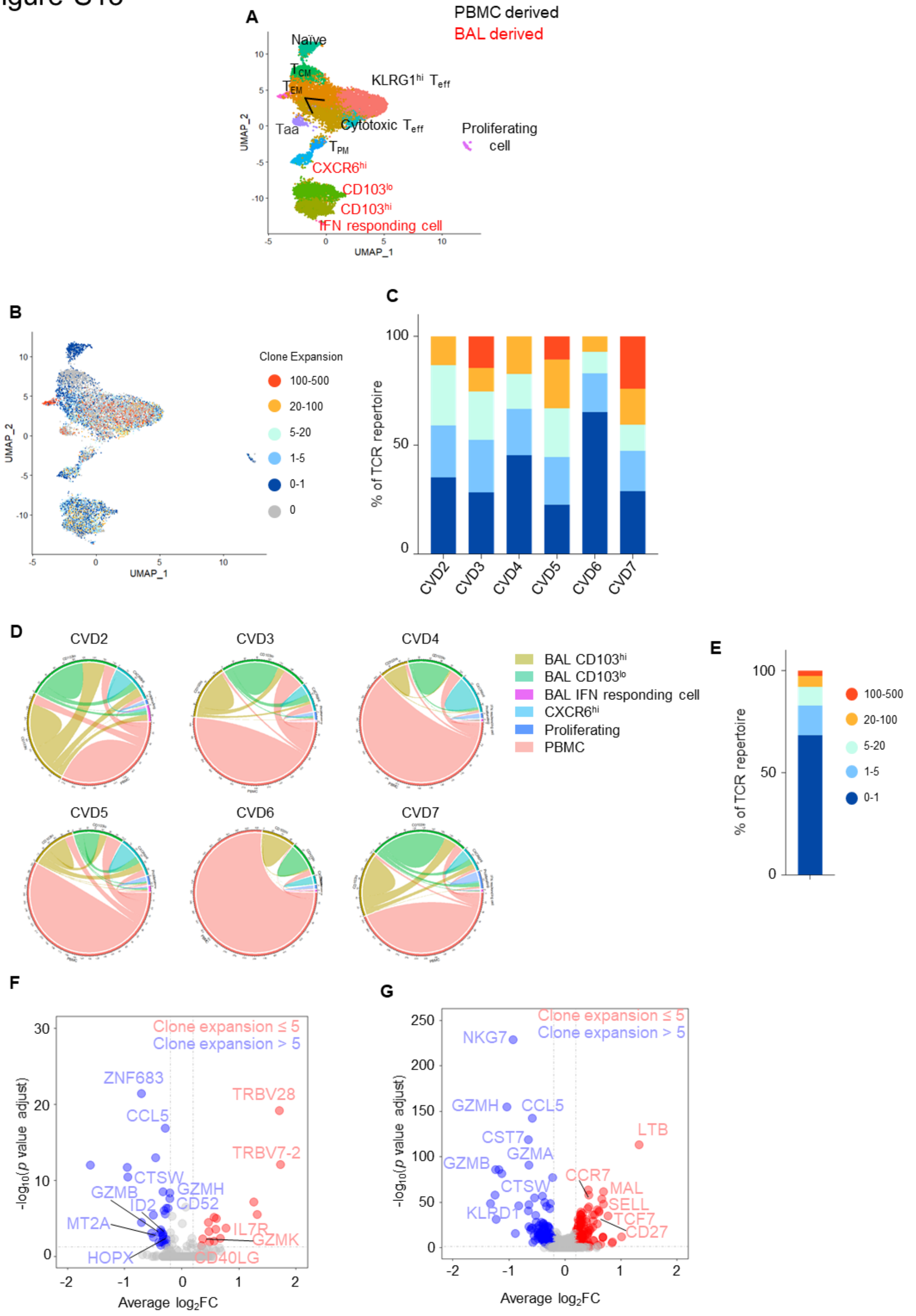

A
PBMC derived
BAL derived
Naïve
T_CM
T_EM
KLRG1hi Teff
Taa
Cytotoxic Teff
Proliferating cell
TPM
CXCR6hi
CD103lo
CD103hi
IFN responding cell
UMAP_1
UMAP_2
B
Clone Expansion
100-500
20-100
5-20
1-5
0-1
0
C
% of TCR repertoire
CVD2
CVD3
CVD4
CVD5
CVD6
CVD7
D
BAL CD103hi
BAL CD103lo
BAL IFN responding cell
CXCR6hi
Proliferating
PBMC
E
F
Clone expansion ≤ 5
Clone expansion > 5
ZNF683
CCL5
TRBV28
TRBV7-2
CTSW
GZMH
GZMB
ID2
CD52
MT2A
IL7R
GZMK
HOPX
CD40LG
-log10(p value adjust)
Average log2FC
G
NKG7
GZMH
CCL5
CST7
GZMA
GZMB
CTSW
KLRD1
LTB
CCR7
MAL
SELL
TCF7
CD27

Fig. S13. scTCR-seq of respiratory and circulating $CD8^+$ T cells in aged COVID-19 convalescents. A. Blood and BAL $CD8^+$ T cell subsets in COVID-19 convalescents revealed by scRNA-seq. T_{eff}, effector T cells; Taa, aged-associated T cells; T_{PM}, peripheral memory T cells. **B.** UMAP plot visualization of TCR clone expansion level from BAL and PBMC $CD8^+$ T cells. **C.** The percentage of each level of TCR clone expansion in total $CD8^+$ T cells from both PBMC and BAL divided by the individual donor. **D.** Chord diagram of shared TCR sequences among BAL $CD8^+$ T cell subclusters and PBMC $CD8^+$ T cells in individual subject. **E.** The percentage of each level of TCR clone expansion in proliferating $CD8^+$ T cells. **F.** Volcano plot showing the differentially expressed genes between TCR clone high expanded (clone > 5) and low expanded (clone ≤ 5) BAL total $CD8^+$ T cells. **G.** Volcano plot showing the differentially expressed genes between TCR clone high expanded (clone > 5) and low expanded (clone ≤ 5) PBMC total $CD8^+$ T cells.

Figure S14

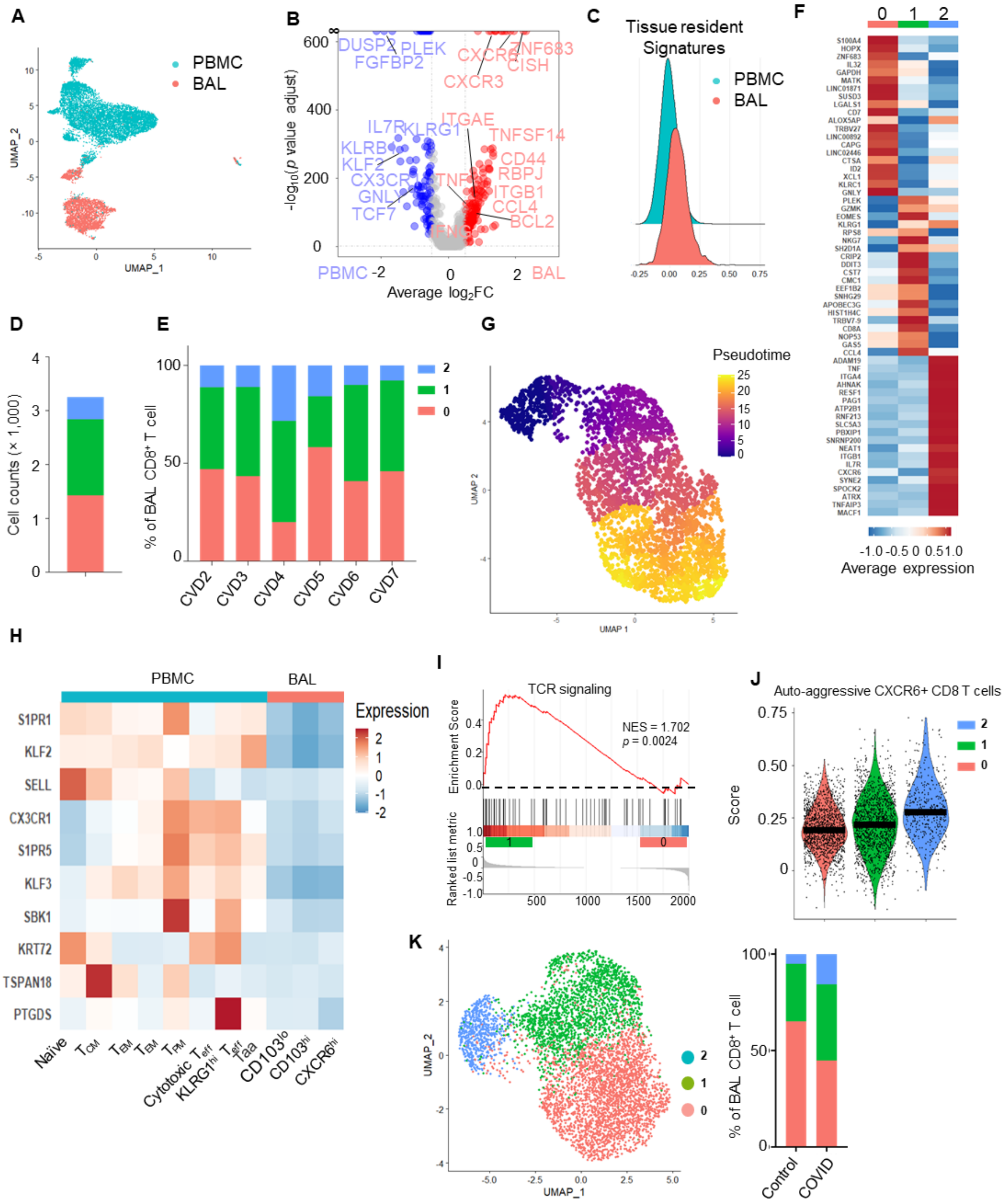
A
PBMC
BAL
UMAP_1
UMAP_2
B
PBMC
BAL
Average log2FC
-log10(p value adjust)
C
Tissue resident Signatures
PBMC
BAL
D
Cell counts (× 1,000)
E
% of BAL CD8+ T cell
CVD2
CVD3
CVD4
CVD5
CVD6
CVD7
F
Average expression
G
Pseudotime
H
PBMC
BAL
Expression
S1PR1
KLF2
SELL
CX3CR1
S1PR5
KLF3
SBK1
KRT72
TSPAN18
PTGDS
Naive
I
TCR signaling
NES = 1.702
p = 0.0024
Enrichment Score
Ranked list metric
J
Auto-aggressive CXCR6+ CD8 T cells
Score
K
UMAP_1
UMAP_2
% of BAL CD8+ T cell
Control
COVID

Fig. S14. scRNA-seq of respiratory and circulating CD8$^+$ T cells in aged COVID-19 convalescents. A. UMAP of CD8$^+$ T cells grouped by the location of the cell source.
B. Volcano plot showing the differentially expressed genes between BAL and PBMC CD8$^+$ T cells. **C.** Expression of the indicated gene signature is elevated between BAL and PBMC CD8$^+$ T cells. **D.** Cell number of each cluster in total tissue-resident CD8$^+$ T cells. **E.** Percentage of each cluster in total BAL CD8$^+$ T cells divided by individual donor. **F.** Heatmap showing the relative expression of the 20 most differentially expressed genes for each cluster of BAL CD8$^+$ T cells. **G.** Pseudotime analysis of BAL CD8$^+$ T cell development. **H.** Heatmap showing relative expression of genes associated with circulation in PBMC and BAL CD8$^+$ T cell clusters. **I.** TCR signaling gene set enrichment between cluster 0 and cluster 1 of respiratory CD8$^+$ T cells. **J.** Expression of the auto-aggressive CXCR6$^+$ CD8$^+$ T cell gene signature (*24*) was elevated in their respective subclusters of BAL CD8$^+$ T cells. **K.** BAL CD8$^+$ T cell subclusters (left) and relative abundance (right) from age-matched control (2), age-unmatched control from GSM4593888 (*33*) and COVID-19 convalescent donors (6) revealed by scRNA-seq.

Figure S15

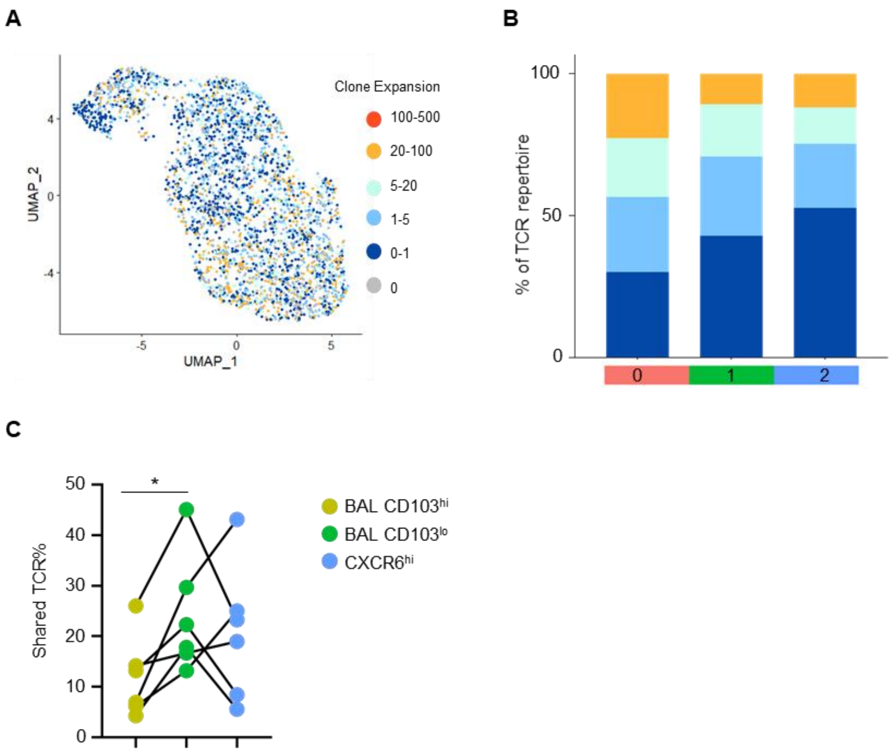

Fig. S15. Shared TCR usage between BAL and PBMC CD8^{+} T cells in aged COVID-19 convalescents. A. UMAP plot visualization of TCR clone expansion level from BAL CD8^{+} T cells. **B.** The level of TCR clone expansion percentage in individual clusters of BAL CD8^{+} T cells. **C.** Percentages of TCR clones shared with PBMC CD8^{+} T cells within the individual BAL CD8^{+} T cell clusters. Paired T test.

Figure S16

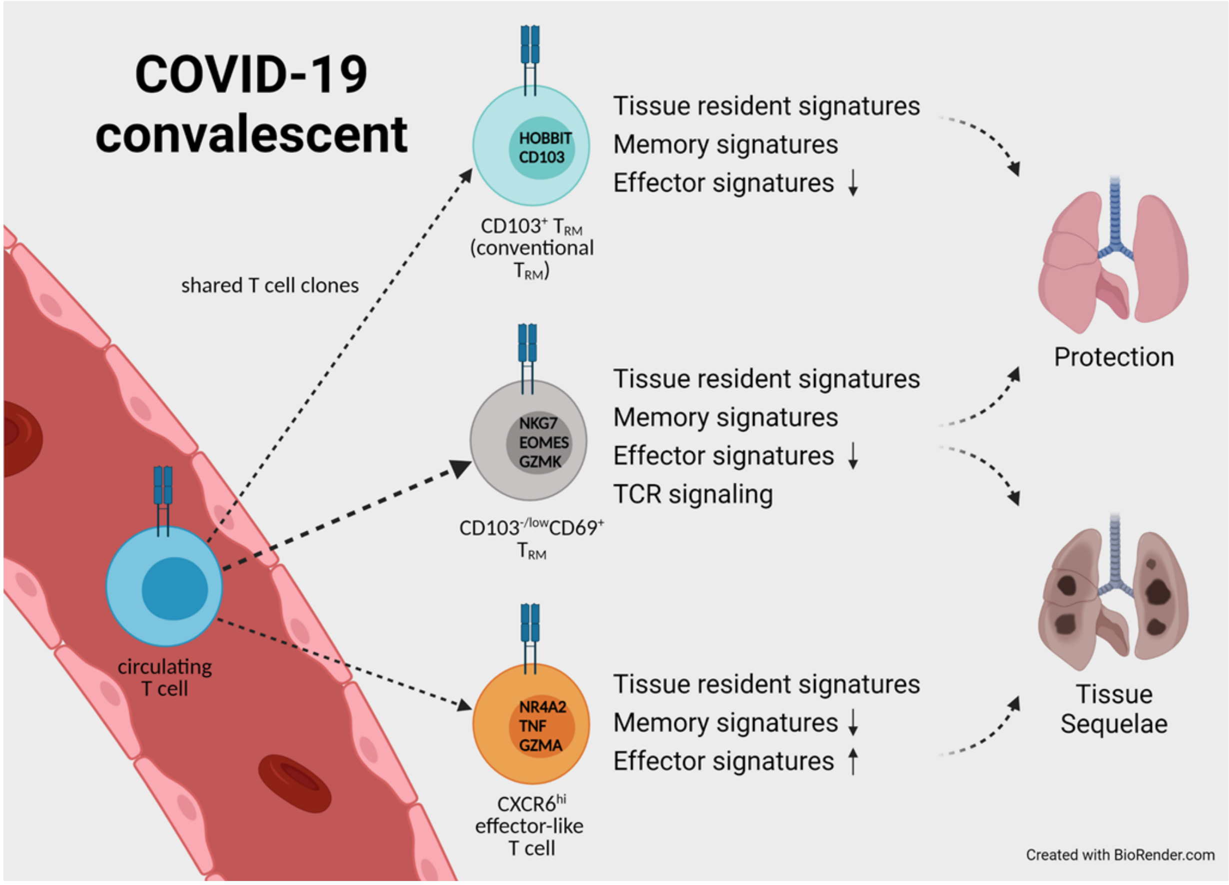

Fig. S16. Model on the potential roles of different respiratory CD8 T cell subsets in the development of chronic tissue sequelae.
